# Integrating genetic mutations and expression profiles for survival prediction of lung adenocarcinoma

**DOI:** 10.1111/1759-7714.13072

**Published:** 2019-04-16

**Authors:** Yueqiang Song, Donglai Chen, Xi Zhang, Yuping Luo, Siguang Li

**Affiliations:** ^1^ Stem Cell Translational Research Center, Tongji Hospital Tongji University School of Medicine Shanghai China; ^2^ Department of Thoracic Surgery, Shanghai Pulmonary Hospital Tongji University School of Medicine Shanghai China

**Keywords:** Driver mutation, gene expression, histologic subtype, lung adenocarcinoma, prognosis

## Abstract

**Background:**

Lung adenocarcinoma (LUAD) is a set of heterogeneous diseases with distinct genetic and transcriptomic characteristics. Since the introduction of the 2011 International Association for the Study of Lung Cancer/American Thoracic Society/European Respiratory Society histologic classification, increasing evidence has provided insights into genomic mutations and rearrangements among individual histologic subtypes of LUAD. However, how genotypic and phenotypic features of LUAD are interconnected is not well understood.

**Methods:**

We obtained the genomic, transcriptomic, and clinical data sets of 488 LUAD patients from The Cancer Genome Atlas database. Advanced statistical models were used to disentangle the interactions between genetic mutations and expression profiles, and to assess the alterations and changes in expression of each histologic subtype. The prognostic impacts of genetic mutations, expression profiles, and clinicopathological features were integrated to predict the outcomes of LUAD patients.

**Results:**

From our data, one or more genetic mutations correlate with expression levels of 6054/18175 (33.3%) genes and explain 8–40% of observed variability in LUAD. The genetic mutations and expression profiles varied remarkably among the histologic subtypes of LUAD, which helped to explain the different prognostic impact based on subtype classification. Genomic, transcriptomic, and clinical data were all shown to have utility for predicting overall and recurrence‐free survival, with the largest contribution from the transcriptome.

**Conclusion:**

Our prediction model integrating genetic mutations, expression profiles, and clinicopathological features exhibited superior accuracy over the current tumor node metastasis staging system to prognosticate outcomes of patients with LUAD (overall survival 67% vs. 55%, recurrence‐free survival 57% vs. 49%; *P* < 0.01).

## Introduction

Non‐small cell lung cancer (NSCLC) is the most common cause of cancer‐related mortality worldwide, leading to over a million deaths each year.^1^, ^2^, ^3^ Recent advances in treating thoracic malignancies have focused on research and development of molecular‐targeted therapy in NSCLC patients, especially driver mutations.^4^ Lung adenocarcinoma (LUAD) is the most common histologic type of NSCLC, with cigarette smoking as the major cause,^5^, ^6^, ^7^ and exhibits high rates of somatic mutation and genomic rearrangement.^1^ Over the past decade, molecular‐targeted therapies have dramatically improved the treatment options for LUAD patients with tumors harboring somatically activated mutations.^8^, ^9^, ^10^, ^11^ A series of driver genes, including *TP53*, *EGFR*, *KRAS*, *ALK*, *BRAF*, *MET*, *RET*, and *ROS1*, are the most frequently mutated genes in LUAD.^1^, ^12^, ^13^ The genomic characterization of LUAD, especially the mutation signatures suggesting potential diverse carcinogenesis pathways, may offer clues for more effective treatments. The high heterogeneity and complexity of LUAD emphasizes the significance of associating genomic findings with clinical outcomes for deeper analysis.^12^, ^14^

Genetic mutations can affect gene expression by means of aberrant transcription, epigenetic regulation, and cell signaling and gene dosage effects.^15^ Gene expression profiles have been shown to exhibit the underlying characteristics of cancer, and are typically used to provide prognostic information for LUAD patients^16^, ^17^, ^18^ and design new drug targets^19^ however, incorporating gene expression‐based methods into clinical practice has been met with difficulty, including overfitting, interpatient histologic heterogeneity, intratumoral heterogeneity, and a lack of accounting for existing clinical variables.^20^, ^21^ Moreover, the limitations of individual biomarkers mean that they cannot be used as reliable classifiers.^16^, ^18^ Genes tend to interact with each other to constitute regulatory networks rather than functioning in isolation.^22^ Therefore, understanding the interactions between gene mutations and expression profiles is critical to unraveling the molecular basis of LUAD.

Currently, the tumor node metastasis (TNM) staging system is used worldwide to predict NSCLC prognosis in clinical practice. In 2011, the International Association for the Study of Lung Cancer/American Thoracic Society/European Respiratory Society (IASLC/ATS/ERS) introduced a new LUAD classification in which invasive adenocarcinomas were mainly categorized into lepidic, acinar, papillary, solid, and micropapillary predominant, or invasive mucinous adenocarcinomas.^7^, ^23^ Since the introduction of this classification, many studies have demonstrated the different prognostic implications among the histologic subtypes of LUAD.^24^, ^25^ Although the genetic features of individual LUAD subtypes have been investigated, the interactive genetic mutations and expression profiles of these histologic subtypes have not been elucidated.^23^, ^26^, ^27^

In the current study, genomic and transcriptomic data from The Cancer Genome Atlas (TCGA) were obtained and integrated to predict the clinical outcomes of LUAD patients. The clinicopathological variables, including the histologic subtypes and the TNM staging system, were also incorporated as predictors. The relationships between genotypes comprising genetic mutations and expression profiles, and phenotypes including histologic subtypes and disease survival were analyzed in LUAD patients. We believe that making genotypic and phenotypic characteristics available will help to improve the molecular diagnosis and personalized precision therapy for LUAD.

## Methods

### Materials

#### The Cancer Genome Atlas data

Genomic and transcriptomic data were collected from TCGA. A total of 517 primary LUAD samples were initially included in our study. Combined expression profiles and driver mutations, as well as survival data, were available for 488/517 LUAD patients. Somatic non‐silent mutation data and clinical variables of the LUAD samples were retrieved using the UCSC xenabrowser (https://xena.ucsc.edu). The clinical annotations included overall survival (OS), age at diagnosis, pathologic findings, and smoking history. The histologic subtypes of LUAD in TCGA (http://www.nature.com/nature/journal/v511/n7511/full/nature13385.html#supplementary-information) cohort were obtained from the supplementary material of previously published studies, and were available for 200/488 patients.^26^, ^28^

#### Selection of recurrent mutant genes and expression data

Fifteen recurrently mutated genes identified in LUAD were included in our mutation screen based on previous studies (Table 1).^1^, ^7^, ^12^, ^13^, ^14^, ^28^, ^29^, ^30^, ^31^ Mutations of each gene were identified in > 10 LUAD samples according to the obtained genomic data. The expression data of 22 435 genes were initially retrieved. After excluding anonymous genes or genes in which the level of expression was unavailable, a total of 18 175 genes were included in the analysis.

**Table 1:** Distribution of demographic and clinical variables of 488 lung adenocarcinoma patients

Characteristics	Number (range)
Age at first diagnosis (median, range)	67 (38–88)
Gender	
Male	226
Female	262
Pathology (histologic subtypes)	
Lepidic predominant	12
Acinar predominant	74
Papillary predominant	23
Micropapillary predominant	23
Solid predominant	58
Invasive mucinous	10
Pathological stage	
I (IA, IB)	257 (121136)
II (IIA, IIB)	113 (46, 67)
III (IIIA, IIIB)	82 (71, 11)
IV	24
Smoking history	
Smoker	73
Non‐smoker	402
Genetic mutations	
*EGFR*	68
*KRAS*	152
*ROS1*	18
*ALK*	29
*BRAF*	40
*MET*	19
*RET*	17
*TP53*	262
*NF1*	53
*ERBB2*	11
*ERBB4*	41
*PIK3CA*	29
*MAP2*	40
*MAP2K1*	10
*CTNNB1*	18

#### Mathematical models

The mathematical models for computing genomic and transcriptomic data and survival can be found in a previous study with a detailed report of all analysis steps.^15^

### Statistical analysis

The primary outcome was OS and the secondary outcome was recurrence‐free survival (RFS). OS was defined as the interval between the diagnosis of cancer and death or the last follow‐up. RFS was measured from the date of initial treatment (after which LUAD patients achieved complete remission/response) to LUAD recurrence. The Kaplan–Meier method was used to compare different categories of prognostic variables. Survival models were fitted using the Cox proportional hazards model. The prognostic accuracy of survival models was evaluated using Harrel's C. To reduce bias on the estimated risk, we used a five‐fold cross‐validating scheme, in which the data was split into five parts of approximately equal size.^15^ One quintile of the data was initially used for training the model and the C statistic was evaluated based on the set of data aside from the others, which was repeated five times. The average C statistic among quintiles as the conclusive estimates was reported.^15^ A value of C = 50% is equivalent to a random guess, while a value of 100% indicates that the survival of the testing cohort was ranked properly.

All statistical analyses were performed using R version 3.4.4. A two‐tailed *P* value of < 0.01 was considered statistically significant.

## Results

A number of confounding factors may lead to variation in interpatient expression profiles in a certain histologic subtype of LUAD. In our study, we focused on decomposing and interpreting the aggregate effects of genetic mutations on gene transcription, given that age, gender, smoking history, and other host factors can possibly confound our analysis.^15^ The clinical characteristics and treatment information of LUAD patients in our cohort is shown in Table 1 and the Supplementary Tables.

### Genetic mutations correlate with transcriptomic expression profiles

**Figure 1:**
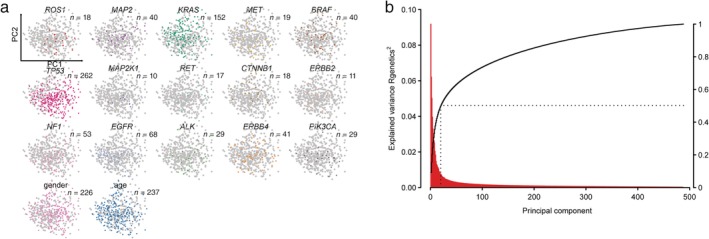
Correlation between genetic mutations and expression profiles. (**a**) Scatter plot of the 15 recurrent mutations, as well as gender and age above or below the median (67 years), overlaying the first two principal components (PCs) of gene expression data of 488 lung adenocarcinomas. (**b**) Explained variance (red, left axis) as a function of PCs 1–488. The cumulative value is shown by the black curve (right axis). The first 20 PCs accounted for 50.2% of the total variation. (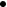) Mutant, (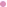) Female, (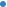) Age>median, (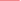) Per PC, (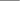) Cumulative.

To map an overview of the main patterns of expression changes, principal component (PC) analysis was performed to reduce multidimensional correlated expression data from 18 175 genes of 488 LUAD patients into a smaller set of mutually‐uncorrelated variables (Fig 1a). We managed to plot the first two PCs to explain the greatest amount of variation in expression data. In our data, 9.2% and 6.2% of the total variability in gene expression was explained by the first two PCs, respectively, while 50.2% of the variance was explained by the first 20 PCs cumulatively (Fig 1b). Genes related to surfactant protein dominated the expression changes associated with PC1, including *SFTPC*, *SCGB1A1*, *SCGB3A1*, *ADH1B*, and *SFTPA1*. Genes that regulate the expression changes associated with PC2 included *PGC*, *SLC14A2*, *DRAIC*, *HMGA2*, and *GPR87*. The observed PCs merely led to a continuum of expression changes rather than clearly separated patient groups. Interestingly, Figure 1a exhibits a correlation between driver mutations and general gene expression profiles by overlaying the status of 15 recurrent (≥ 10 patients) mutations on the first two PCs. For example, *ROS1* and *ALK* mutations tended to have high scores on PC1, whereas *CTNNB1* mutations coincided with high scores on PC2. Indeed, these general associations do not indicate necessary causation but probably explain the correlation between individual mutations and a given PC. In addition, Supplementary Figure 1 shows the Pearson and Spearman correlation coefficients between genetic mutations and gene expression.

### Deconvolution of the interactions between driver mutations and gene expression levels

Because a number of potential variables can affect the expression of genes, a linear modeling approach was applied to measure the association of expression levels on a gene‐by‐gene basis with driver mutations and other confounding factors. Briefly, the model assumes that each mutation leads to a set of expression changes and that the expression profile in each case with a complicated genotype harboring multiple mutations is the sum of the changes resulting from each alteration.

**Figure 2:**
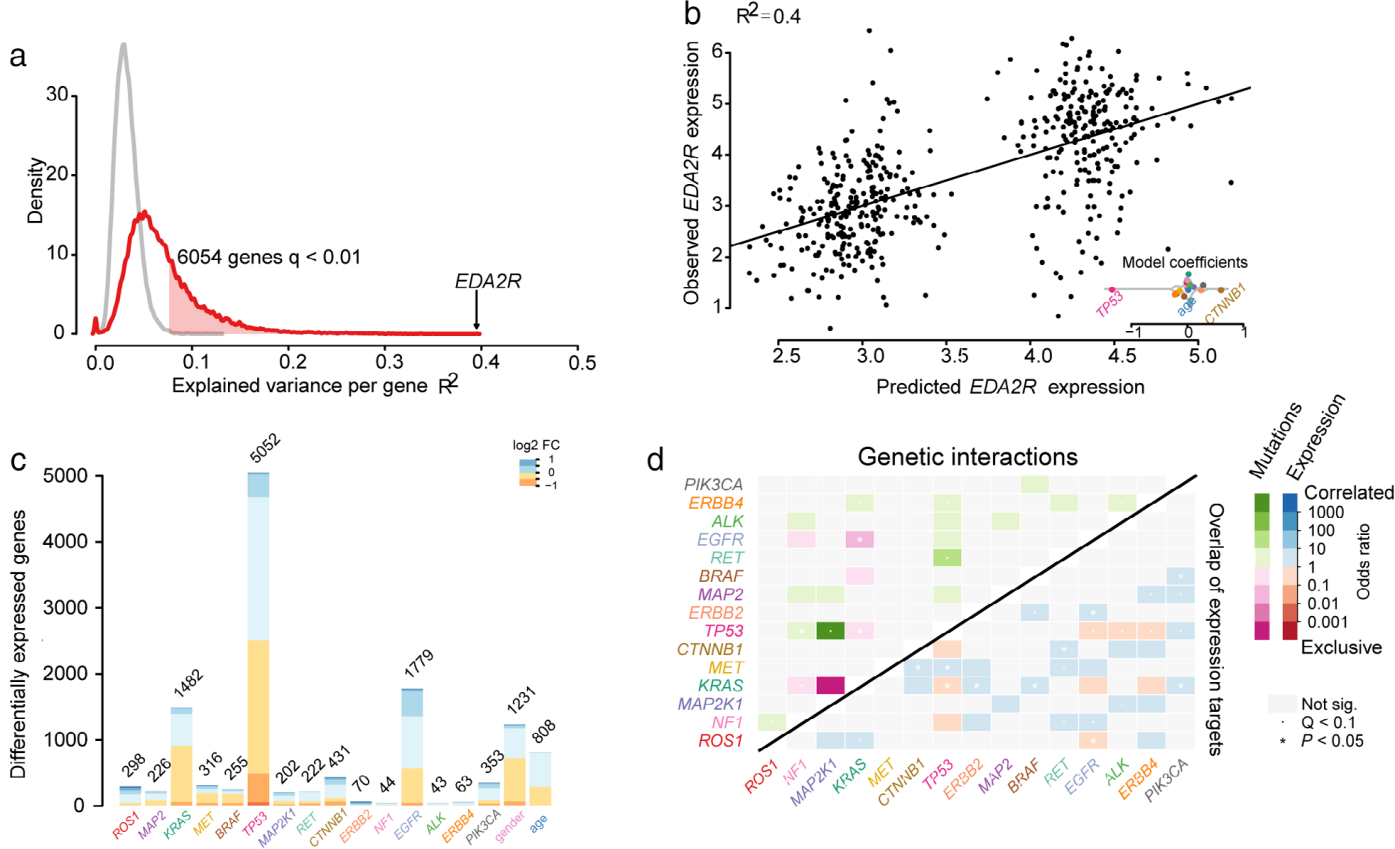
(**a**) Distribution of the variance explained by genetic mutations. (**b**) Scatter plot of the correlation between predicted and observed expression values for the *EDA2R* gene. The model coefficients showed the changed levels of *EDA2R* expression given different alterations. (**c**) Numbers of target genes with statistically significant mutation‐interactive expression (moderated F‐test; false discovery rate < 0.01; *n* = 488) for the 15 recurrent mutations and demographics. The associated logarithmic expression fold change (FC) is indicated by color. (**d**) Heatmap exhibiting pairwise mutations (upper triangle) and overlapped target genes associated with each mutation (lower triangle). Co‐occurrence/high overlap is represented by green/blue, while mutual exclusivity is highlighted by pink/red. (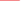) Observed, (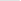) Random.

From our data, the whole transcriptome of LUAD was inevitably affected by genetic driver mutations, with expression changes of 6054/18175 (33.3%) genes significantly associated with at least one mutation (false discovery rate‐adjusted moderated F‐statistic < 0.01) (Fig 2a). Mutations of these genes accounted for at least R^2^ = 8% of the interpatient expression variability. Notably, the strongest association was observed in *EDA2R*, a gene encoding the transmembrane protein of the tumor necrosis factor receptor, which achieved R^2^ = 40% between mutations and expression changes (Fig 2a). The presence of *TP53* and *CTNNB1* mutations can largely account for the observed variability of *EDA2R* expression, which resulted from the strong downregulation and upregulation of EDA2R messenger RNA (mRNA) (Fig 2b).

The linear model enabled us to detect the set of expression changes associated with a given mutation from a mutation‐centric view. As shown in Figure 2c, each mutation targets different numbers of genes, the expression of which is differentially affected. Mutation in *TP53* was the most powerful and frequent, which altered the expression of 5052 genes. Mutations in *EGFR* and *KRAS* were correlated with 1779 and 1482 target genes in LUAD patients, respectively (Fig 2c).

To explore the co‐occurrence or mutual exclusivity between mutant genes, we analyzed the genetic interactions among pairs of driver mutations based on the sets of overlapped target genes. Based on our data, *TP53* and *MAP2K1* shared a highly significant pattern of concurrent mutations, while *EGFR* and *KRAS* exhibited a pattern of mutually exclusive mutations (Fig 2d).

### Identification of genotypic signatures among individual histologic subtypes

**Figure 3:**
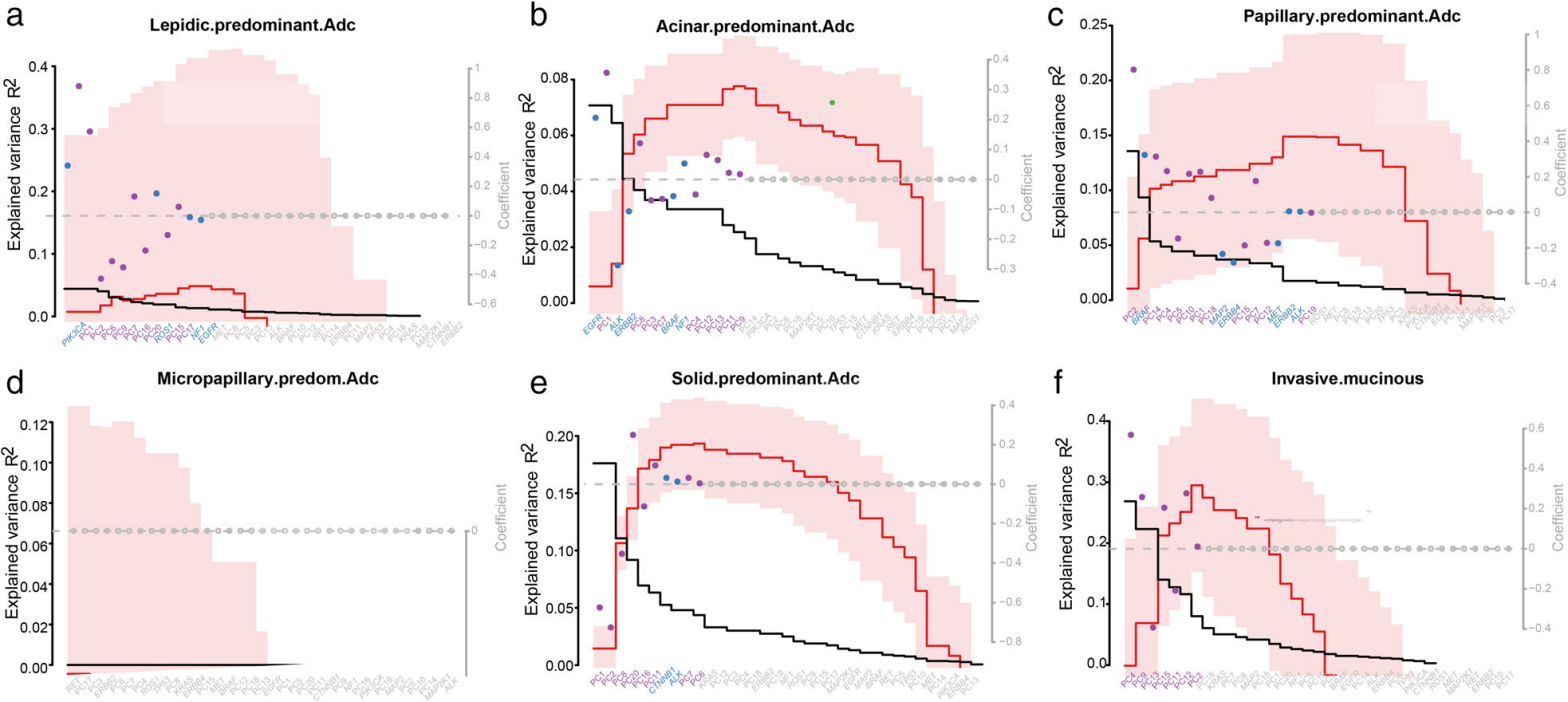
A LASSO penalized model to explain the variance using the 15 recurrent mutations and first 20 transcriptome principal components ordered by the occurrence for each histologic subtype of lung adenocarcinoma: (**a**) lepidic, (**b**) acinar, (**c**) papillary, (**d**) solid, and (**e**) micropapillary predominant, or (**f**) invasive mucinous adenocarcinoma (Adc; red line ± 1 standard deviation; five‐fold cross validation). The explained variance R^2^ of the histologic subtypes is maximized in the optimal model. (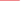) Explained variance Rgenetics^2^, (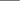) Lasso penalty λ, (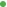) Model coefficient β.

**Figure 4:**
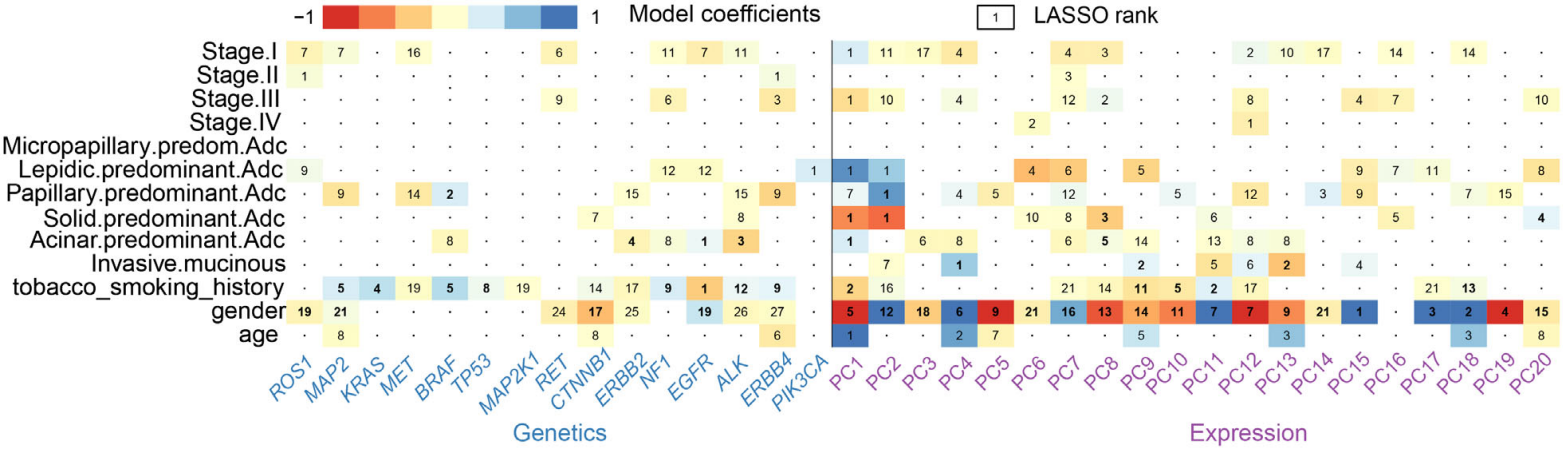
Heatmap of the optimal model coefficients for the histologic subtypes, pathological stages, and demographics. Coefficients based on the LASSO penalized model are in color. The relative importance of variables is numbered in order on each tile. Bold fonts indicate highly significant coefficients.

Histologic subtype is a prognostic variable and significant pathologic phenotype in LUAD, which acts as a determinant for implementing postoperative adjuvant therapy. Generalized linear models were used to associate the common genetic mutations and the first 20 PCs of the transcriptome with histologic subtypes (Fig 3a–f), age, gender, smoking history (Supplementary Fig 2a–c), and pathologic stages (Supplementary Fig 3a–d), which facilitated the identification of the most important genotypic signatures associated with each clinicopathological variable. As shown in Figure 3a and b, the presence of *PIK3CA* mutations and PC1 were the strongest predictors in lepidic‐predominant LUAD. The presence of *EGFR* mutations and PC1 were the most powerful predictors in acinar‐predominant LUAD; however, no actionable mutations were detected as significant predictors in micropapillary and solid predominant or invasive mucinous LUAD (Fig 3d–f). Figure 4 summarizes the associations across driver mutations, expression changes, and clinicopathological variables, including histologic subtypes and pathologic stages.

### Comparison of predictive accuracy between our integrated survival model and the tumor node metastasis staging system

**Figure 5:**
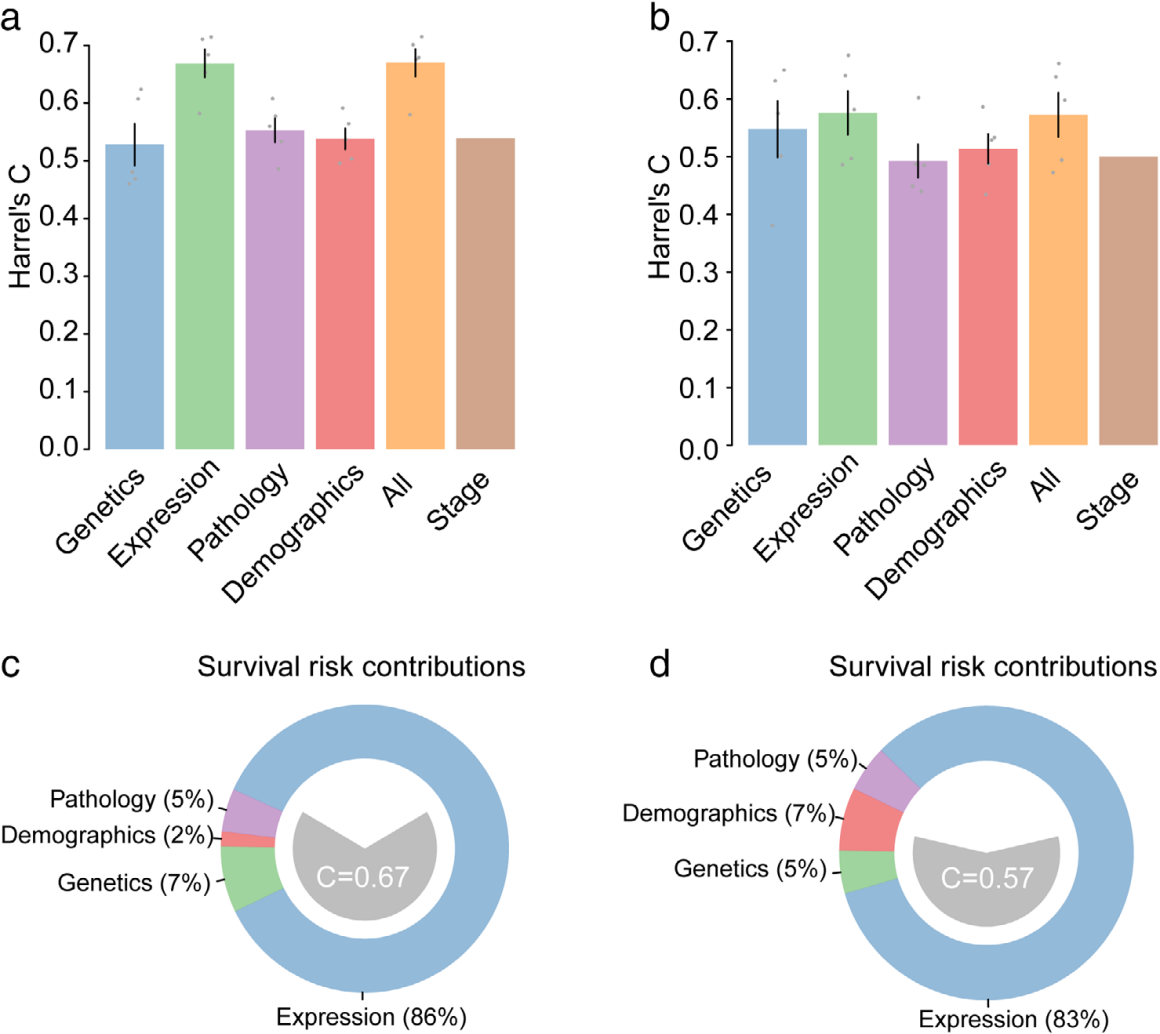
Bar plot of prognostic accuracy in Cox proportional hazards models to predict (**a**) overall survival (OS) and (**b**) recurrence‐free survival (RFS), using genetic expression, histologic subtypes, demographics, and the Tumor Node Metastasis staging system. Bars exhibit the average across five parts in which the data are five‐fold cross validated (grey points). The error bar indicates the standard deviation of the mean. Distribution of risk contributions in the (**c**) OS and (d) RFS prediction model using all covariates.

To accurately prognosticate the outcomes of patients with LUAD, we combined the predictors, including genomic data, transcriptomic data, and clinicopathological variables, which shared a high degree of mutual interdependency, to calculate patient risk. After comparing the prognostic power of different types of data, we observed that our model, which integrated genetic mutations and expression profiles with clinicopathological variables, exhibited superior predictive accuracy over the TNM staging system (OS 67% vs. 55%, RFS 57% vs. 49%; *P* < 0.01) (Fig 5a,b). Integrating different data types elevated the predictive accuracy to an optimum value (C statistic = 67%), similar to the value resulting from expression data alone (Supplementary Figs 4,5). Moreover, decomposing the survival risk contributions by each data type indicated that the predictive accuracy was further improved by contributions from genetics (7%) and pathology (5%) (Fig 5c,d).

## Discussion

LUAD is a set of heterogeneous diseases with distinct genetic and histologic characteristics.^32^ Since the introduction of the 2011 IASLC/ATS/ERS histologic classification, an increasing number of studies have attempted to assess genomic mutations and rearrangements and the lineage relationships among histologic subtypes of LUAD^23^, ^27^, ^32^, ^33^ However, few in‐depth studies have been conducted to analyze the relationships between genetic mutations and expression profiles among the histologic subtypes of LUAD. Moreover, the interrelationship between genotypes and phenotypes of LUAD has not yet been interconnected.

The last decade witnessed the increasing prevalence of multimodal data for survival analysis concerning common diseases.^34^, ^35^ In 2012, Selamat *et al.* took the lead, performing genome‐analysis of DNA methylation integrated with mRNA in LUAD,^36^ followed by comprehensive molecular profiling of LUAD by TCGA Research Network.^1^ Although another study recently focused on the association of omics features with histopathology patterns in LUAD,^2^ none of these studies unraveled the association between genetic mutations and expression profiles. In our study, we managed to disentangle the relationships between genetic mutations, expression profiles, histologic subtypes, and disease survival by bioinformatics. Indeed, this is the first study to provide a comprehensive analysis of associations across driver mutations, expression changes, clinicopathological variables, and outcomes using TCGA data.

Our study revealed not only clarified mechanisms, but also other surprising findings. Generally, we found that genetic mutations are interactive with expression profiles in LUAD, which is consistent with previous findings. Schabath *et al.* indicated that *STK11* and *TP53* mutations are associated with a *KRAS* mutation‐associated gene expression signature, as well as signatures of tumor proliferation and immune surveillance response in LUAD.^37^ Mutations in *TP53* are correlated with lower mRNA expression levels, whereas mutations in *EGFR* and *KRAS* are associated with higher mRNA expression levels in LUAD,^38^ a result supported by Planck *et al.* 39 Mutations in *EGFR* (13.9%) are mutually exclusive with those in *KRAS* (31.4%) and are significantly correlated with smoking status, which is consistent with the results of previous studies.^1^, ^28^ The absence of potentially actionable mutations in micropapillary and solid predominant LUAD may account for the poorest outcomes in patients with these histologic subtypes.^40^, ^41^

A previous study showed that the *TP53* mutation in lung cancer might shorten the survival of patients treated with targeted therapy for oncogenic driver mutations.^29^ *MAP2K1* mutations have been shown to indicate a special subset of LUAD that might potentially benefit from MEK inhibitors.^42^ In our study, *TP53* and *MAP2K1* exhibited a highly significant pattern of concurrent mutations, which indicates that *MAP2K1* is a novel target for LUAD with *TP53* alterations. The identification of major mutations and expression changes in individual histologic subtypes of LUAD might help oncologists with their diagnoses and prescriptions for inoperable LUAD patients whose specimens can be obtained by biopsy rather than complete resection.

Based on our data, predictive accuracy was significantly improved when integrating multiple data types into a prognostic model. Specifically, genetics, expression, demographics (age and gender), and clinicopathological characteristics all add supplementary information into the current TNM staging system for survival prediction. Notably, the expression profiles that reflected the status of the transcriptome highlighted the personalized information and made a tremendous contribution to the survival risk prediction. Our study exhibits superiority over previous studies of survival prediction. Zhao *et al.* developed a TCGA‐based model using 20 filtered genes that showed lower accuracy (61.5% vs. 67%) for predicting OS compared to ours.^43^ Gao *et al.* established a TCGA‐based survival model using methylation‐driven genes that was also inferior to ours (66% vs. 67%).^44^

There were several limitations to our study. First, the investigated cohort was retrieved from TCGA and no external validation cohort was available. Second, because of the retrospective nature of our study, performance and selection bias was inevitable. The highly selected cohort in our study is not representative of daily practice and could skew the conclusions, as only a quarter of the included patients had advanced disease and only 18% were smokers. The histologic subtype was only available in a small number of patients, leading to an inevitably discrepant finding on the driver mutations in lepidic‐predominant LUAD compared to other studies.^27^, ^33^ Third, TCGA does not distinguish between pathologic and clinical staging, and stage IIIB–IV LUAD is a contraindication for radical surgery. Thus, the specimen and staging information of patients with advanced‐stage LUAD probably relied on biopsy findings that could lead to potential bias. More comprehensive sequencing data with larger samples, as well as deeper deciphering of the genotypes, could pave the way for an understanding of unclarified phenotypic variability and to precisely predict clinical outcomes of LUAD patients.

In conclusion, our study disentangled the interrelationships between LUAD genotypes and phenotypes and developed a prediction model integrating genetic mutations, expression profiles, and clinicopathological features, which exhibited superior accuracy over the current TNM staging system for prognosticating the outcomes of LUAD patients.

## Disclosure

No authors report any conflict of interest.

## Supporting information


**Figure S1.** The correlation heat map for (a) Pearson and (b) Spearman coefficients between genetic mutations and gene expression.

**Figure S2.** A LASSO penalized model to explain the variance using the 15 recurrent mutations and first 20 transcriptome principal components ordered by their occurrence for (a) age, (b) gender, and (c) smoking history of lung adenocarcinoma.

**Figure S3.** A LASSO penalized model to explain the variance using the 15 recurrent mutations and first 20 transcriptome principal components ordered by their occurrence for stage (a) I, (b) II, (c) III, and (d) IV lung adenocarcinoma.

**Figure S4.** Overall survival stratified by different prognostic factors in a multivariate survival model using Kaplan–Meier curves.

**Figure S5.** Recurrence‐free survival stratified by different prognostic factors in a multivariate survival model using Kaplan–Meier curves.

**Table S1.** Treatment information of the entire study cohort.
Table S2. Treatment information of the patient cohort with histologic type available.
Table S3. Targeted therapy for patients with actionable mutations (*n* = 32).

**Appendix S1.** A detailed report of the complete code used in the analysis.
