# Supplementary figures and images for: Integrating genetic mutations and expression profiles for survival prediction of lung adenocarcinoma
Source: Thorac Cancer. 2019 Apr 16;10(5):1220–8. doi: 10.1111/1759-7714.13072 (PMC6501026; doi:10.1111/1759-7714.13072)

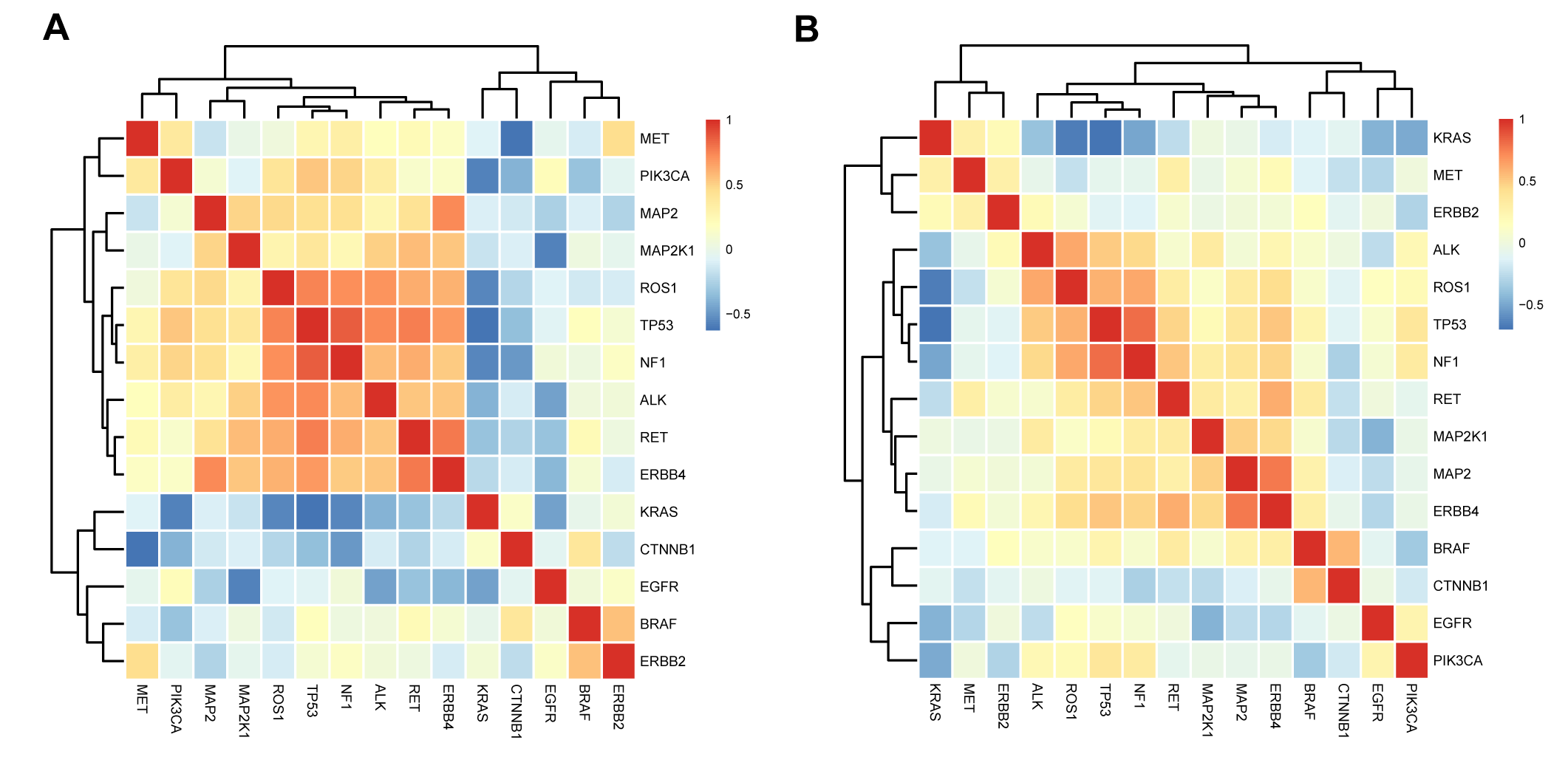

Supplement: Supplementary file 1 — Figure S1. The correlation heat map for (a) Pearson and (b) Spearman coefficients between genetic mutations and gene expression. [file TCA-10-1220-s001.tif]

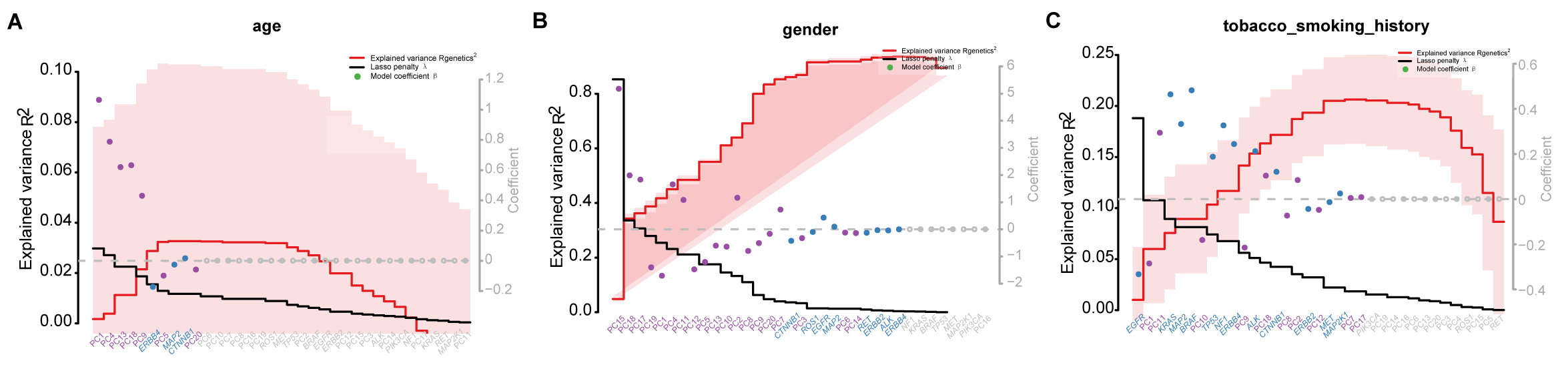

Supplement: Supplementary file 2 — Figure S2. A LASSO penalized model to explain the variance using the 15 recurrent mutations and first 20 transcriptome principal components ordered by their occurrence for (a) age, (b) gender, and (c) smoking history of lung adenocarcinoma. [file TCA-10-1220-s002.tif]

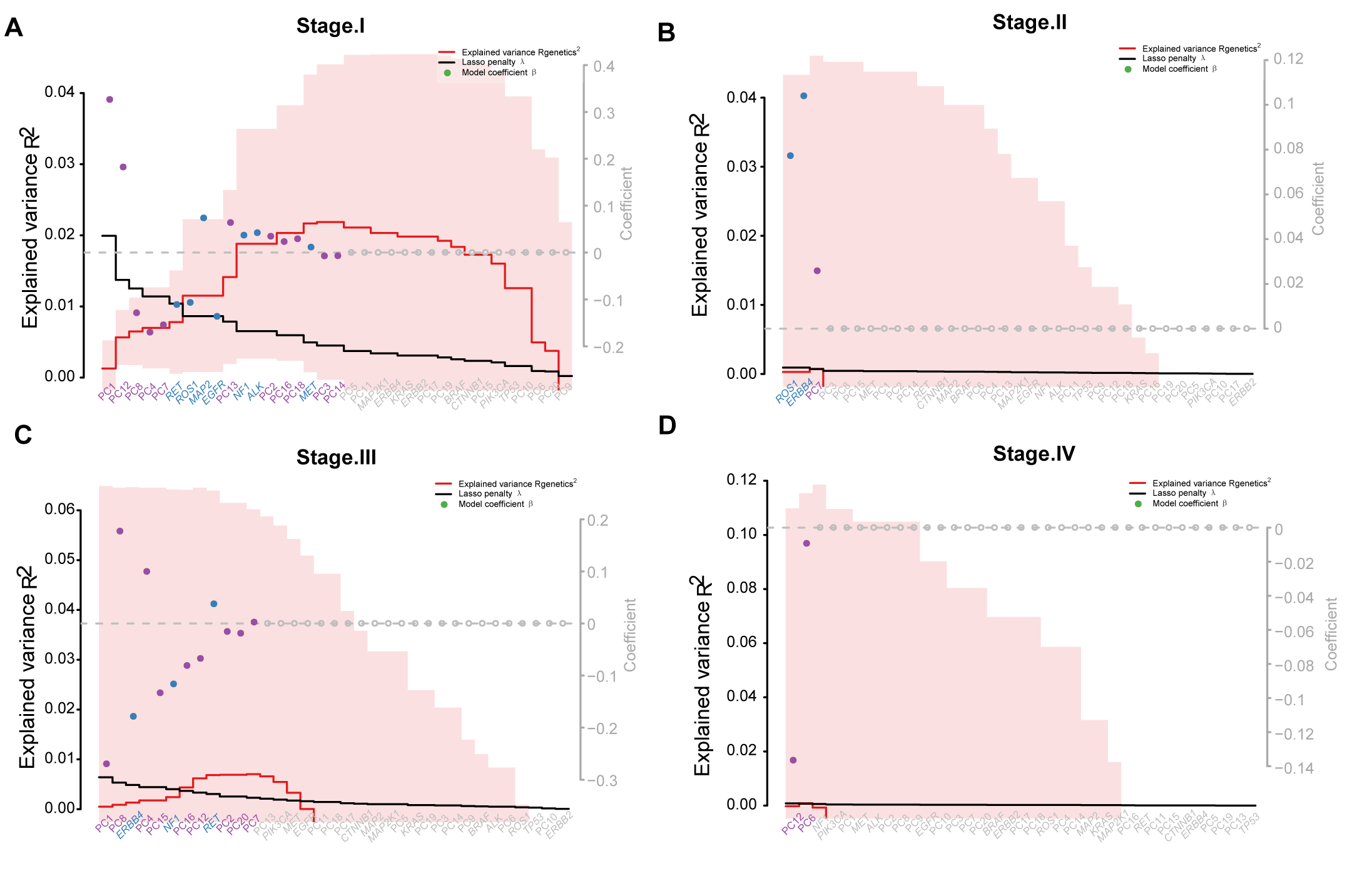

Supplement: Supplementary file 3 — Figure S3. A LASSO penalized model to explain the variance using the 15 recurrent mutations and first 20 transcriptome principal components ordered by their occurrence for stage (a) I, (b) II, (c) III, and (d) IV lung adenocarcinoma. [file TCA-10-1220-s003.tif]

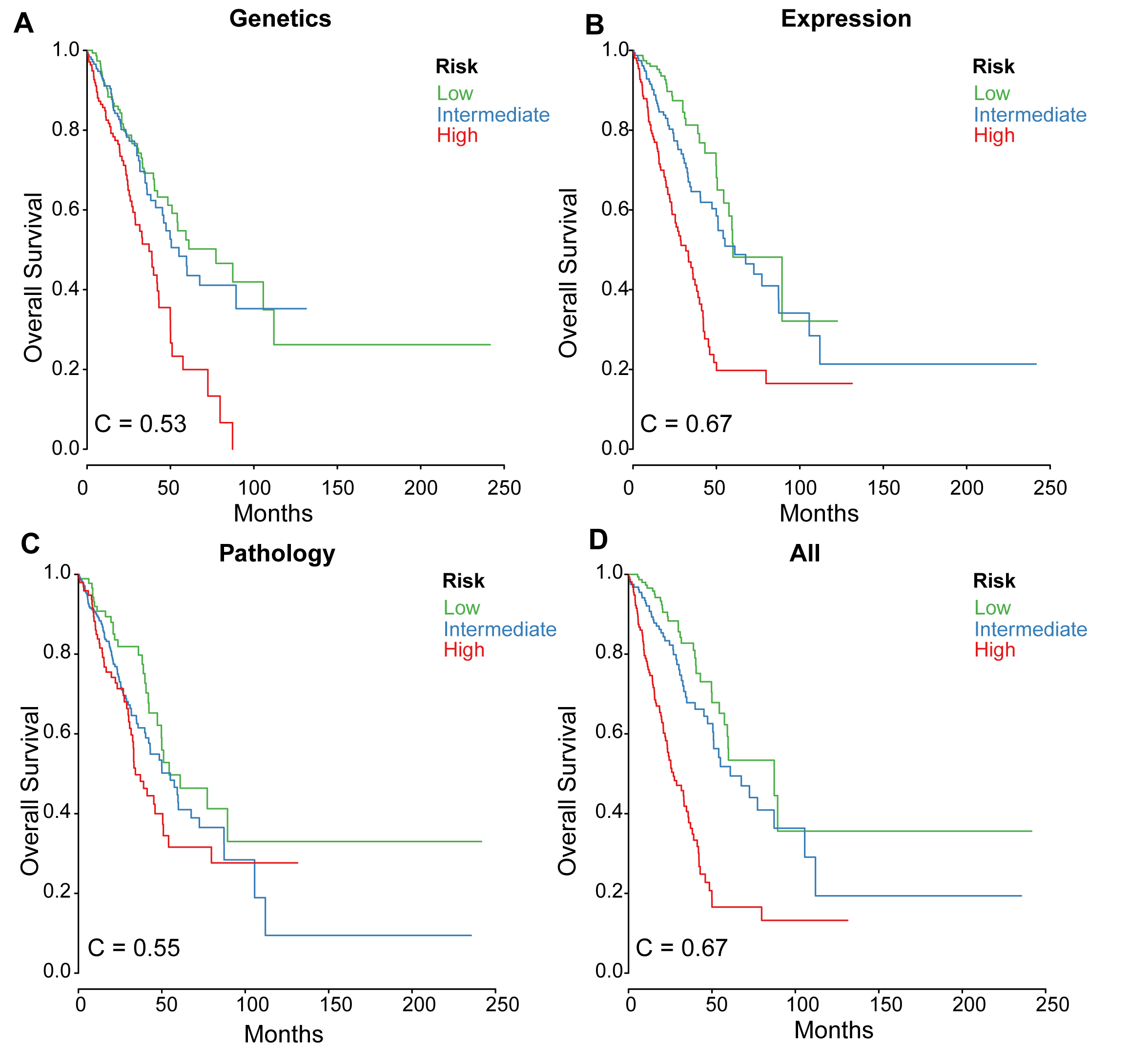

Supplement: Supplementary file 4 — Figure S4. Overall survival stratified by different prognostic factors in a multivariate survival model using Kaplan–Meier curves. [file TCA-10-1220-s004.tif]

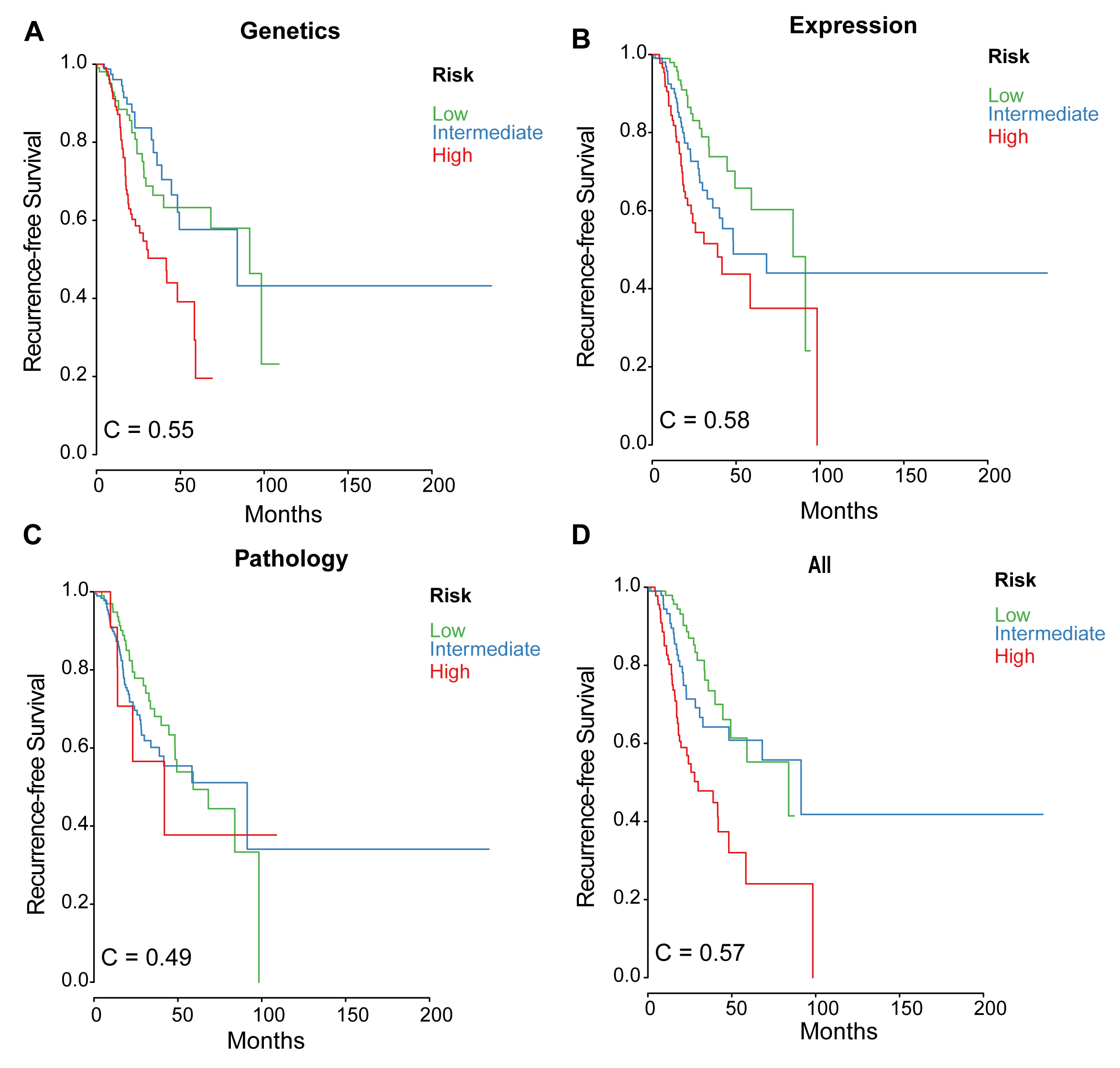

Supplement: Supplementary file 5 — Figure S5. Recurrence‐free survival stratified by different prognostic factors in a multivariate survival model using Kaplan–Meier curves. [file TCA-10-1220-s005.tif]
