# Supplementary material for: Integrating genetic mutations and expression profiles for survival prediction of lung adenocarcinoma
Source: Thorac Cancer. 2019 Apr 16;10(5):1220–8. doi: 10.1111/1759-7714.13072 (PMC6501026; doi:10.1111/1759-7714.13072)
Supplement: Supplementary file 6 — Table S1. Treatment information of the entire study cohort. Table S2. Treatment information of the patient cohort with histologic type available. Table S3. Targeted therapy for patients with actionable mutations (n = 32). [file TCA-10-1220-s006.docx]

| Supplementary Table 1. Treatment information of the entire cohort in our study | | | | | |
| --- | --- | --- | --- | --- | --- |
| TNM stage | Additional pharmaceutical therapy | Radiation therapy | Targeted/molecular therapy | Additional surgery locoregional procedure | Additional surgery metastatic procedure |
| Stage I | 22 | 14 | 23 | 7 | 7 |
| Stage II | 23 | 11 | 57 | 3 | 8 |
| Stage III | 8 | 23 | 47 | 2 | 3 |
| Stage IV | 6 | 6 | 14 | 0 | 3 |
| Total patients (Yes/No) | 60/85 | 55/378 | 144/287 | 12/109 | 21/82 |

| Supplementary Table 2. Treatment information of the patient cohort with histologic type available | | | | | |
| --- | --- | --- | --- | --- | --- |
| TNM stage | Additional pharmaceutical therapy | Radiation therapy | Targeted/molecular therapy | Additional surgery locoregional procedure | Additional surgery metastatic procedure |
| Stage I | 10 | 9 | 10 | 1 | 3 |
| Stage II | 13 | 6 | 24 | 2 | 6 |
| Stage III | 3 | 10 | 20 | 1 | 1 |
| Stage IV | 2 | 1 | 4 | 0 | 0 |
| Total (Yes/No) | 28/42 | 26/146 | 59/113 | 4/55 | 10/36 |

| Supplementary Table 3. Targeted therapy for patients with actionable mutations (n=32) | | | |
| --- | --- | --- | --- |
| TNM stage | ROS1 (n=5) | EGFR (n=22) | ALK (n=5) |
| Stage I | 0 | 3 | 1 |
| Stage II | 3 | 6 | 3 |
| Stage III | 2 | 9 | 1 |
| Stage IV | 0 | 4 | 0 |
