# Supplementary material for: Integrating genetic mutations and expression profiles for survival prediction of lung adenocarcinoma
Source: Thorac Cancer. 2019 Apr 16;10(5):1220–8. doi: 10.1111/1759-7714.13072 (PMC6501026; doi:10.1111/1759-7714.13072)
Supplement: Supplementary file 7 — Appendix S1. A detailed report of the complete code used in the analysis. [file TCA-10-1220-s007.pdf]

Supplementary

Supplementary code for Integrating Genetic Mutations and Expression Profiles for Survival Prediction of Lung Adenocarcinoma

This document contains the complete code used in the analysis. It is purely written in R using a series of R and Bioconductor packages. This report has been generated using the knitr R package .The mathematical models for computing genomic and transcriptomic data and survival can be found in a previous study (Gerstung et al., 2015) with a detailed report and code of the most analysis steps. We do appreciate Gerstung's sharing.

Load necessary libraries

```
library(reshape2)
library(limma)
library(org.Hs.eg.db)
library(hgu133plus2.db )
library(RColorBrewer)
library(cgdsr)
library(CoxHD)
library(mg14) ## From github.com/mg14/mg14
library(xtable)
library(Hmisc)
library(RColorBrewer)
library(AnnotationDbi)
library(affy)
library(gcrma)
library(VennDiagram)
library(GenomicRanges)
library(GenomicFeatures)
library(rtracklayer)
library(biomaRt)
library(glmnet)
library(survival)
library(Hmisc)
library(randomForestSRC)
library(mg14)
set1 <- brewer.pal(9,"Set1")
#source("suppData/mg14.R")
source("C:/Users/ASUS/Documents/R/win-library/3.4/mg14-master/R/mg14.R")
```

1. Data preprocessing

1.1 Download expression data from TCGA by cBioportal

```
entrez <-  unique(AnnotationDbi::select(hgu133plus2.db, keys = keys(hgu133plus2.db),
                                        columns = c("ENTREZID"))$ENTREZID)
#Load expression data from cBio portal
mycgds <-  CGDS("http://www.cbioportal.org/")
all_TCGA_studies <- getCancerStudies(mycgds)
tcgaLUAD <- getCancerStudies(mycgds)[115,1]
casesLUAD <- getCaseLists(mycgds,tcgaLUAD)[9,1]#"luad_tcga_rna_seq_v2_mrna"
#g <-  lapply(split(as.numeric(entrez),seq_along(entrez)%/%500),
```

```
#              function(genes)getProfileData(mycgds,genes,
#              getGeneticProfiles(mycgds,tcgaLUAD)[6,1],casesLUAD))
## load in batches of 500,need a lot of time
#g <- do.call("cbind", g)
#save(g, file="g.RData")
load(file="g.RData")
```

1.2 Normalize expression data and filter the genes

```
tcgaExprN=t(g)
rownames(tcgaExprN) <- sub("\\.","-",sub("^[0-9]+\\.","",rownames(tcgaExprN))) ## Fix rownames
s <- AnnotationDbi::select(org.Hs.eg.db, rownames(tcgaExprN), "ENTREZID", "SYMBOL")
m <- match(rownames(tcgaExprN), s$SYMBOL)
sum(is.na(m))
```

```
## [1] 0
```

```
rownames(tcgaExprN) <- s$ENTREZID[m]# genesymbol to ENTREZID
colnames(tcgaExprN) <- gsub("\\.","-",colnames(tcgaExprN))
tcgaExprN <- tcgaExprN[rowSums(is.na(tcgaExprN))==0 & !is.na(rownames(tcgaExprN)),]
tcgaExpr=log(tcgaExprN+1)
```

1.3 Load mutation and clinical data

Download the files by UCSC xenabrowser (https://xena.ucsc.edu)

```
cli=read.table("LUAD_clinicalMatrix",sep="\t", row.names=1, header=TRUE,
               check.names=FALSE,na.strings ="",stringsAsFactors=F)
Mut_gene=read.table("mutation_broad_gene",sep="\t",header=TRUE,
                    check.names=FALSE,na.strings ="",stringsAsFactors=F)
#choose mutation
Genes_cli=c("AKT1","BRAF","CBL","CTNNB1","EGFR","ERBB2","ERBB4","HRAS",
            "KRAS","MAP2K1","MET","NRAS","PIK3CA","PTPN11")
Genes_cli_Canonical_mut=c("ALK","RET","MAP2","ROS1")
Genes_another=c("FGFR4","NF1","TP53")
Genesum=c(Genes_cli,Genes_cli_Canonical_mut,Genes_another)
Expr=as.data.frame(tcgaExpr)
Mut=Mut_gene[Mut_gene$sample%in% c(Genesum) ,colnames(Mut_gene) %in% c(colnames(Expr),"sample")]
rownames(Mut)=Mut$sample
Mut=Mut[,-1]
Mutt=as.data.frame(t(Mut))
mat=match(rownames(Mutt),colnames(Expr))
Expr=Expr[,mat]
Mutt$gender=cli[colnames(Expr),"gender"]
Mutt$gender[Mutt$gender=="MALE"]=as.numeric(1)
Mutt$gender[Mutt$gender=="FEMALE"]=as.numeric(0)
for (i in 1:ncol(Mutt)) {
  Mutt[,i]=as.integer(Mutt[,i])
}
# at least in 10 samples
minF=10
Mutt = Mutt[,colSums(Mutt)>=minF]
Mutt$age=scale(cli[colnames(Expr),"age_at_initial_pathologic_diagnosis"],center=TRUE, scale=FALSE)
Expr1=Expr[,colnames(Expr)%in%colnames(Mut)]
```

```
geneExpr=Expr1
design = cbind(offset=1,Mutt)
design0 <- design
for(j in 1:ncol(design))
  design[is.na(design[,j]),j] <- mean(design[,j], na.rm=TRUE)
head(design)
```

```
##                  offset ROS1 MAP2 KRAS MET BRAF TP53 MAP2K1 RET CTNNB1
## TCGA-44-4112-01       1    0    0    0   0    0    0      0   0      1
## TCGA-NJ-A4YP-01       1    0    0    1   0    0    0      0   0      0
## TCGA-86-8278-01       1    0    0    0   0    0    0      0   0      0
## TCGA-05-4430-01       1    0    0    0   0    0    1      0   0      0
## TCGA-44-6778-01       1    0    0    0   0    0    1      1   0      0
## TCGA-97-A4M1-01       1    0    0    0   0    0    1      0   0      0
##                  ERBB2 NF1 EGFR ALK ERBB4 PIK3CA gender        age
## TCGA-44-4112-01      0   0    0   1     1      0      0  -5.695096
## TCGA-NJ-A4YP-01      0   0    0   0     0      0      1 -13.695096
## TCGA-86-8278-01      0   0    0   0     0      0      0  -2.695096
## TCGA-05-4430-01      0   0    0   0     0      0      0  -6.695096
## TCGA-44-6778-01      0   0    0   0     1      0      1  -6.695096
## TCGA-97-A4M1-01      0   0    1   0     0      0      0 -13.695096
```

2 Model fitting (from Gerstung et al., 2015)

```
glm = lmFit(geneExpr[,rownames(design)], design = design )
glm = eBayes(glm)
F.stat <- classifyTestsF(glm[,-1],fstat.only=TRUE) # remove offset
glm$F <- as.vector(F.stat)
df1 <- attr(F.stat,"df1")
df2 <- attr(F.stat,"df2")
if(df2[1] > 1e6){ # Work around bug in R 2.1
  glm$F.p.value <- pchisq(df1*glm$F,df1,lower.tail=FALSE)
}else
  glm$F.p.value <- pf(glm$F,df1,df2,lower.tail=FALSE)

set.seed(35)
rlm <- lmFit(geneExpr[,rownames(design)], apply(design, 2, sample))
rlm <- eBayes(rlm)
F.stat <- classifyTestsF(rlm[,-1],fstat.only=TRUE)
rlm$F <- as.vector(F.stat)
df1 <- attr(F.stat,"df1")
df2 <- attr(F.stat,"df2")
if(df2[1] > 1e6){ # Work around bug in R 2.1
  rlm$F.p.value <- pchisq(df1*rlm$F,df1,lower.tail=FALSE)
}else
  rlm$F.p.value <- pf(rlm$F,df1,df2,lower.tail=FALSE)

#F.stat <- classifyTestsF(glm[,2:(ncol(design)-2)],fstat.only=TRUE) ## All genetics & cytogenetics
F.stat <- classifyTestsF(glm[,2:(ncol(design)-2)],fstat.only=TRUE)
df1 <- attr(F.stat,"df1")
df2 <- attr(F.stat,"df2")
F.p.value <- pchisq(df1*F.stat,df1,lower.tail=FALSE)
R.stat <- classifyTestsF(rlm[,2:(ncol(design)-2)],fstat.only=TRUE) ## Random
```

```r
Rall = 1 - 1/(1 + glm$F * (ncol(design)-1)/(nrow(design)-ncol(design)))
Rgenetics = 1 - 1/(1 + F.stat * (ncol(design)-1)/(nrow(design)-ncol(design)))
Pgenetics = 1 - 1/(1 + R.stat * (ncol(design)-1)/(nrow(design)-ncol(design)))
names(Rgenetics) <- names(Pgenetics) <- names(Rall) <-  rownames(geneExpr)
```

Plot the variance explained by genetics

```r
par(bty="n", mgp = c(2,.33,0), mar=c(3,2.5,1,1)+.1, las=1, tcl=-.25, xpd=NA)
d <- density(Pgenetics,bw=1e-3)
f <- 1#  nrow(gexpr)/512
plot(d$x, d$y * f, col='grey', xlab=expression(paste("Explained variance per gene ", R^2)),
     main="", lwd=2, type="l", ylab="", xlim=c(0,0.5))
title(ylab="Density", line=1.5)
d <- density(Rgenetics, bw=1e-3)
r <- min(Rgenetics[p.adjust(F.p.value,"BH")<0.01])
x0 <- which(d$x>r)
polygon(d$x[c(x0[1],x0)], c(0,d$y[x0])* f, col=paste(set1[1],"44",sep=""), border=NA)
lines(d$x, d$y* f, col=set1[1], lwd=2)
#points(d$x[x0[1]], d$y[x0[1]]*f, col=set1[1], pch=16)
text(d$x[x0[1]], d$y[x0[1]]*f, pos=4, paste(sum(Rgenetics > r), "genes q < 0.01"))
genesname=s[s$ENTREZID %in%
              names(Rgenetics[Rgenetics==max(Rgenetics[p.adjust(F.p.value,"BH")<0.05])]),]$SYMBOL
arrows(Rgenetics[Rgenetics==max(Rgenetics[p.adjust(F.p.value,"BH")<0.05])], par("usr")[4]/7,
       Rgenetics[Rgenetics==max(Rgenetics[p.adjust(F.p.value,"BH")<0.05])],
       par("usr")[4]/50, length=0.05)
text(Rgenetics[Rgenetics==max(Rgenetics[p.adjust(F.p.value,"BH")<0.05])], par("usr")[4]/8,
     genesname, font=3, pos=3)
legend("topright", bty="n", col=c(set1[1], "grey"), lty=1, c("Observed","Random"), lwd=2)
```

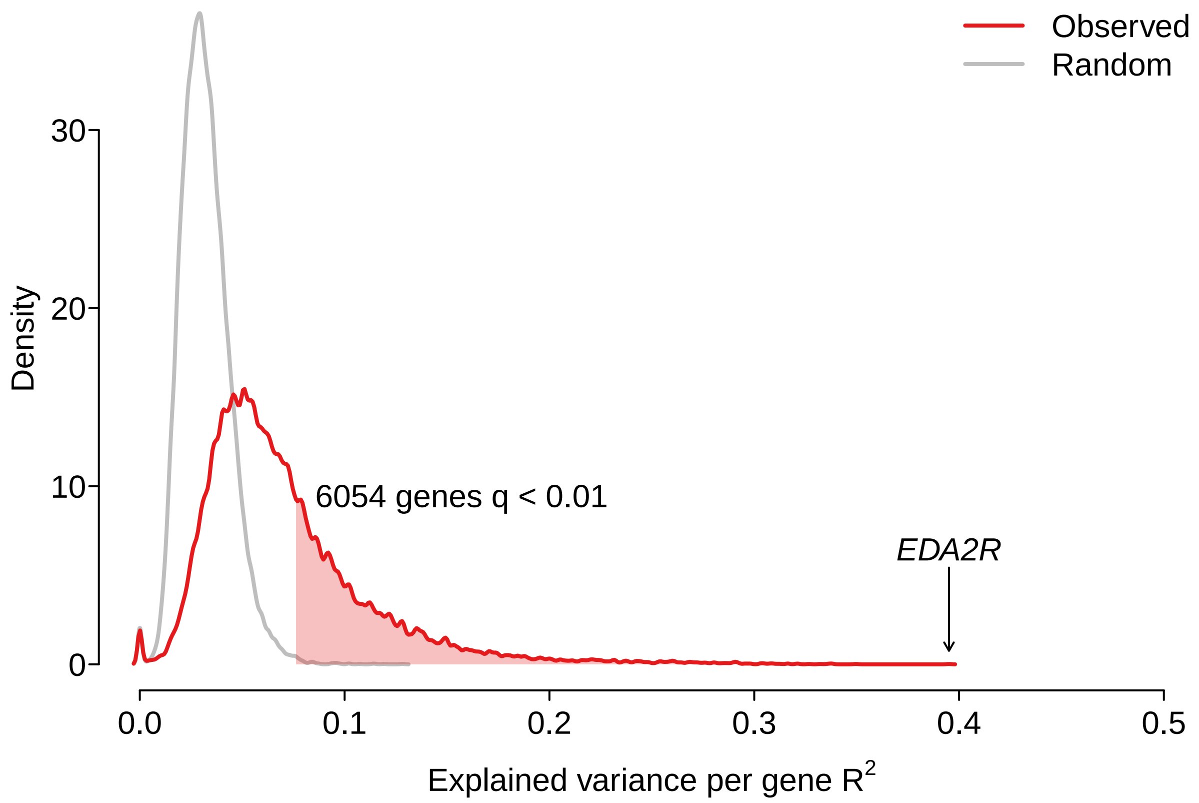


```
Gene=names(Rgenetics[Rgenetics==max(Rgenetics[p.adjust(F.p.value,"BH")<0.01])])
#geneExpr[Gene,]
```

3 Calculate the Coefficient of the Model

```
# define colors
colMutations = c(brewer.pal(8,"Set1")[-6], rev(brewer.pal(8,"Dark2")),
                 brewer.pal(7,"Set2"))[c(1:12,16:19,13:15)]
o <- order(apply(col2rgb(colMutations),2,rgb2hsv)[1,])
colMutations <- colMutations[rev(o)][(4*1:19 +15) %% 19 + 1]
names(colMutations) <- colnames(design)[-1]
#Predictions
glmPrediction <- glm$coefficients %*% t(design)
rlmPrediction <- rlm$coefficients %*% t(design)
par(bty="n", mgp = c(1.5,.33,0), mar=c(2.5,2.5,1,1)+.1, las=1, tcl=-.25)
plot(glmPrediction[Gene,], geneExpr[Gene,rownames(design)],
     ylab=expression(paste("Observed ",italic('EDA2R'), " expression")),
     xlab=expression(paste("Predicted ",italic('EDA2R'), " expression")), pch=16, cex=.8)
abline(0,1)
u <- par("usr")
par(xpd=NA)
y <- glm$coefficients[Gene,-1]+glm$coefficients[Gene,1]
u <- par("usr")
x0 <- rep(u[3]+1,ncol(design)-1)
y0 <- u[4] + 0.05*(u[4]-u[3]) - rank(-y)/length(y) * (u[4]-u[3])/1.2
d <- density(y)
```

```
lines(d$x/3+3.5, d$y/20+1+u[3], col="grey")
lines(d$x/3+3.5,-d$y/20+1+u[3], col="grey")
points(x=y/3+3.5, y=x0+violinJitter(y, magnitude=0.25)$y, col=colMutations, pch=16)
text(x=glm$coefficients[Gene,1]/3+3.5, y= 1.5 +u[3], "Model coefficients", cex=0.8)
w <- glm$p.value[Gene,-1] < 0.01
rotatedLabel(y[w]/3+3.5, x0[w]+0.1, labels=colnames(design)[-1][w],
             font=ifelse(grepl("[[:lower:]]", colnames(design)[-1]),1,3)[w],
             cex=.66, pos=1, col=colMutations[w])
axis(at=(-1:1 + glm$coefficients[Gene,1])/3+3.5, labels=-1:1, side=1,
     cex.axis=.8, line=-1, mgp = c(1.5,.05,0), tcl=-.15)
#mtext(at=l$coefficients[1], line=-2, side=1, "Coefficients", cex=.8)
text(u[1],u[4], substitute(paste(R^2==r),list(r=round(Rgenetics[Gene],2))), pos=4)
```

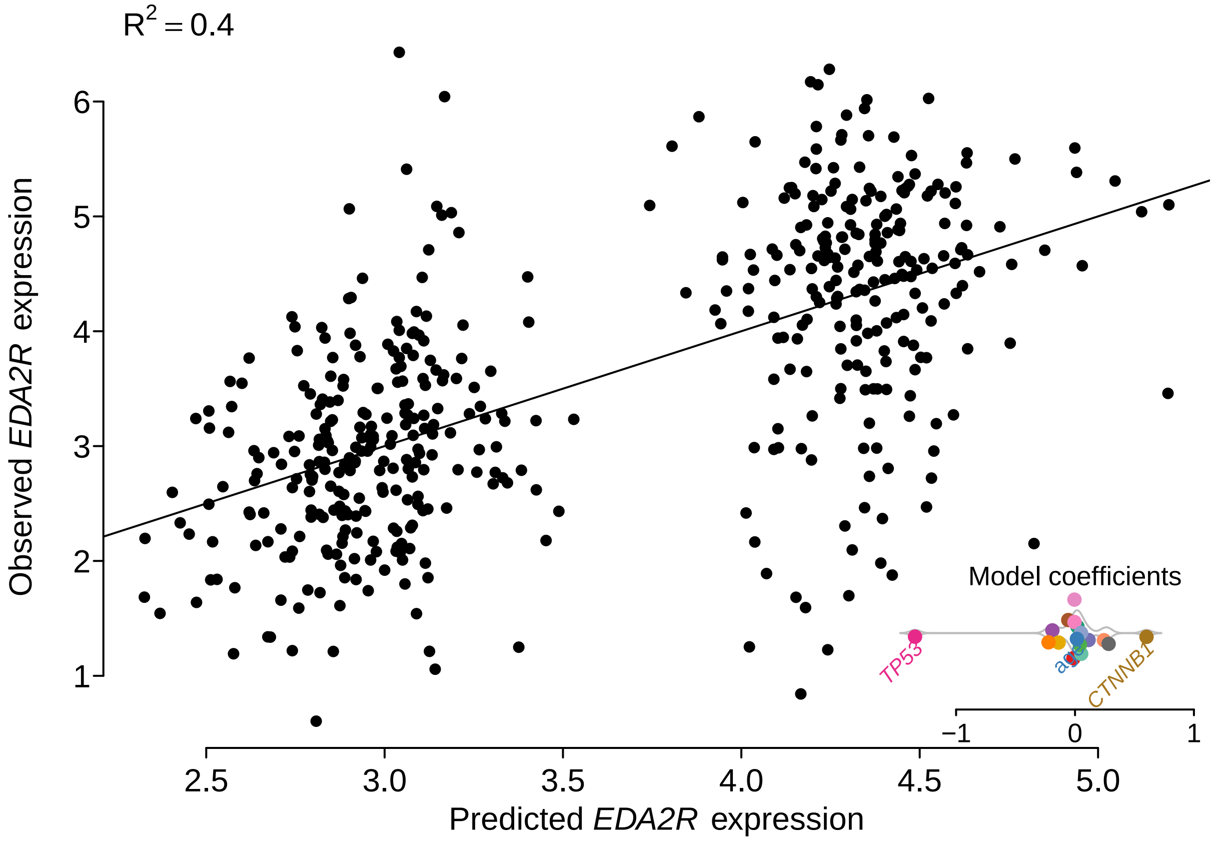

4 Differentially expressed genes

```
testResults <- decideTests(glm, method="hierarchical",adjust.method="BH", p.value=0.05)[,-1]
significantGenes <- sapply(1:ncol(testResults), function(j){
  c <- glm$coefficients[testResults[,j]!=0,j+1]
  table(cut(c, breaks=c(-5,seq(-1.5,1.5,l=7),5)))
})
colnames(significantGenes) <- colnames(testResults)
par(bty="n", mgp = c(2.5,.33,0), mar=c(3,3.3,2,0)+.1, las=2, tcl=-.25)
b <- barplot(significantGenes, las=2, ylab = "Differentially expressed genes",
             col=brewer.pal(8,"RdYlBu"), legend.text=FALSE , border=0, xaxt="n")
##, col = set1[simple.annot[names(n)]], border=NA)
```

```r
rotatedLabel(x0=b, y0=rep(10, ncol(significantGenes)), labels=colnames(significantGenes),
             cex=.7, srt=45, font=ifelse(grepl("[[:lower:]]", colnames(design))[-1], 1,3),
             col=colMutations)
clip(0,50,0,6000)
#text(b+0.2, colSums(n)+50, colSums(n), pos=3, cex=.7, srt=90)
x0 <- 18.5
n=4
image(x=x0+c(0,0.8), y=par("usr")[4]+seq(-100*n,100*n,l=9)[3:7], z=matrix(1:4, ncol=4),
      col=brewer.pal(8,"RdYlBu")[3:7], add=TRUE)
#image(x=x0+c(0,0.8), y=par("usr")[4]+seq(-100,100,l=9), z=matrix(1:8, ncol=8),
#       col=colMutations, add=TRUE)
text(x=x0+1.5, y=par("usr")[4]+seq(-50*n,50*n,l=3), format(seq(-1,1,l=3),2), cex=0.66)
#lines(x=rep(x0+.8,2), y=par("usr")[4]+c(-75*n,75*n))
segments(x0+.8,par("usr")[4]+seq(-75*n,75*n,l=7)[2:6],x0+.9,par("usr")[4]+seq(-75*n,75*n,l=7)[2:6])
text(x0+0.8, par("usr")[4]+100*n, "log2 FC", cex=.66)
rotatedLabel(b-0.1, colSums(significantGenes), colSums(significantGenes), pos=3, cex=, srt=45)
```

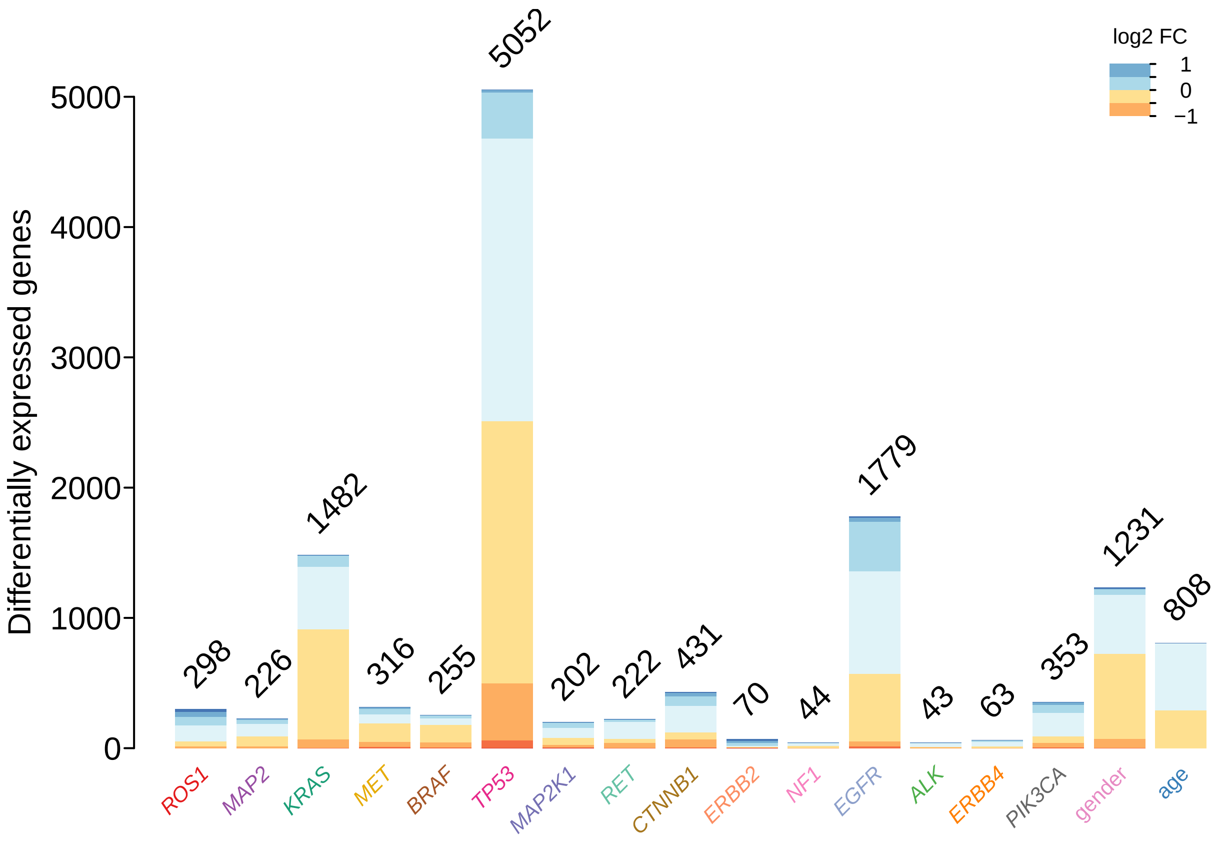

5 Test for Mutations interactions

Here we want to compare how mutational co-occurrence and the sets of transcriptional changes are interlinked

```r
genomicData = design[,colnames(design)[2:(ncol(design)-2)]]
interactions <- interactionsGenes <- sapply(1:ncol(genomicData), function(i)
  sapply(1:ncol(genomicData), function(j) {
```

```r
    f<- try(fisher.test(genomicData[,i], genomicData[,j]), silent=TRUE);
    if(class(f)=="try-error")
      0
    else
        ifelse(f$estimate>1, -log10(f$p.val),log10(f$p.val))
    } ))
oddsRatio <- oddsGenes <- sapply(1:ncol(genomicData), function(i)
  sapply(1:ncol(genomicData), function(j) {
    f<- try(fisher.test(genomicData[,i] + .5, genomicData[,j] +.5), silent=TRUE);
    if(class(f)=="try-error")
      f=NA
    else
      f$estimate
    } ))
w <- p.adjust(glm$F.p.value,"BH")<0.05
oddsExpression <- sapply(1:ncol(genomicData), function(i)
  sapply(1:ncol(genomicData), function(j) {
    f<- try(fisher.test(abs(testResults[w,i]), abs(testResults[w,j])), silent=TRUE);
    if(class(f)=="try-error")
      f=NA
    else
      f$estimate
    } ))
interactionsExpression <- sapply(1:ncol(genomicData), function(i)
  sapply(1:ncol(genomicData), function(j) {
    f<- try(fisher.test(abs(testResults[w,i]), abs(testResults[w,j])), silent=TRUE);
    if(class(f)=="try-error")
      0
    else
      ifelse(f$estimate>1, -log10(f$p.val),log10(f$p.val))
    } ))
oddsRatio[lower.tri(oddsRatio)] <- oddsExpression[lower.tri(oddsExpression)]
interactions[lower.tri(interactions)] <- interactionsExpression[lower.tri(interactions)]

diag(interactions) <- NA
diag(oddsRatio) <- NA
colnames(oddsRatio) <- rownames(oddsRatio) <- colnames(interactions) <-
  rownames(interactions) <- colnames(genomicData)
oddsRatio[10^-abs(interactions) > 0.05] = 1
oddsRatio[oddsRatio<1e-3] = 1e-4
oddsRatio[oddsRatio>1e3] = 1e4
logOdds=log10(oddsRatio)

reorder <- function(M, o){
  u <- M
  u[lower.tri(u)] <- t(M)[lower.tri(M)]
  u <- u[o,o]
  l <- M
  l[upper.tri(u)] <- t(M)[upper.tri(M)]
  l <- l[o,o]
  R <- u
  R[lower.tri(R)] <- l[lower.tri(R)]
  return(R)
```

```
}

#plot
par(bty="n", mgp = c(2,.5,0), mar=c(4,4,2,4)+.1, las=2, tcl=-.33)
m <- nrow(oddsRatio)
n <- ncol(oddsRatio)
o = c(1,11,7,3,4,9,6,10,2,5,8,12:m)#h$order#c(h$order,(length(h$order) +1):ncol(interactions))
r <- reorder(log10(oddsRatio),o)
r[lower.tri(r)] <- NA
image(x=1:n, y=1:m, r, col=brewer.pal(9,"PiYG"), breaks = c(-4:0-.Machine$double.eps,0:4),
      xaxt="n", yaxt="n", xlab="",ylab="", xlim=c(0, n+4), ylim=c(0, n+4))
r <- reorder(log10(oddsRatio),o)
r[upper.tri(r)] <- NA
image(x=1:n, y=1:m, r, col=brewer.pal(9,"RdBu"), breaks = c(-4:0-.Machine$double.eps,0:4), add=TRUE)
mtext(side=2, at=1:n, colnames(oddsRatio)[o],
      font=ifelse(grepl('[[:lower:]]',colnames(oddsRatio)[o]),1,3),
      col=colMutations[1:16][o])
rotatedLabel(x0=1:n, y0=rep(0.5, n), colnames(oddsRatio)[o],
             font=ifelse(grepl('[[:lower:]]',colnames(oddsRatio)[o]),1,3), srt=45,
             cex=.9,
             col=colMutations[1:16][o])
abline(h=0:n+.5, col="white", lwd=.5)
abline(v=0:n+.5, col="white", lwd=.5)
text(x=n/2, y=m+.5, "Genetic interactions", pos=3)
text(x=n+1, y=m/2, "Overlap of expression targets", pos=3, srt=270)
q <- p.adjust(10^-abs(reorder(interactions,o)), method="BH")
p <- p.adjust(10^-abs(reorder(interactions,o)), method="holm")
w = arrayInd(which(q < .1), rep(m,2))
points(w, pch=".", col="white", cex=1.5)
w = arrayInd(which(p < .05), rep(m,2))
points(w, pch="*", col="white")
image(y = 1:8 +6, x=rep(n,2)+c(2,2.5)+1, z=matrix(c(1:8), nrow=1), col=brewer.pal(8,"PiYG"), add=TRUE)
image(y = 1:8 +6, x=rep(n,2)+c(2.5,3)+1, z=matrix(c(1:8), nrow=1), col=brewer.pal(8,"RdBu"), add=TRUE)
axis(side = 4, at = seq(1,7) + 6.5,  tcl=-.15, label=10^seq(-3,3), las=1, lwd=.5)
mtext(side=4, at=10, "Odds ratio", las=3, line=3)
par(xpd=NA)
text(x=n+2.2, y=15, "Correlated", pos=4)
text(x=n+2.2, y=6-.2, "Exclusive", pos=4)
points(x=rep(n,2)+3.5, y=1:2, pch=c("*","."))
image(x=rep(n,2)+c(2,3)+1, y=(3:4) -0.5, z=matrix(1), col=brewer.pal(3,"BrBG"), add=TRUE)
mtext(side=4, at=1:3, c("P < 0.05", "Q < 0.1","Not sig." ), line=0.2)
```

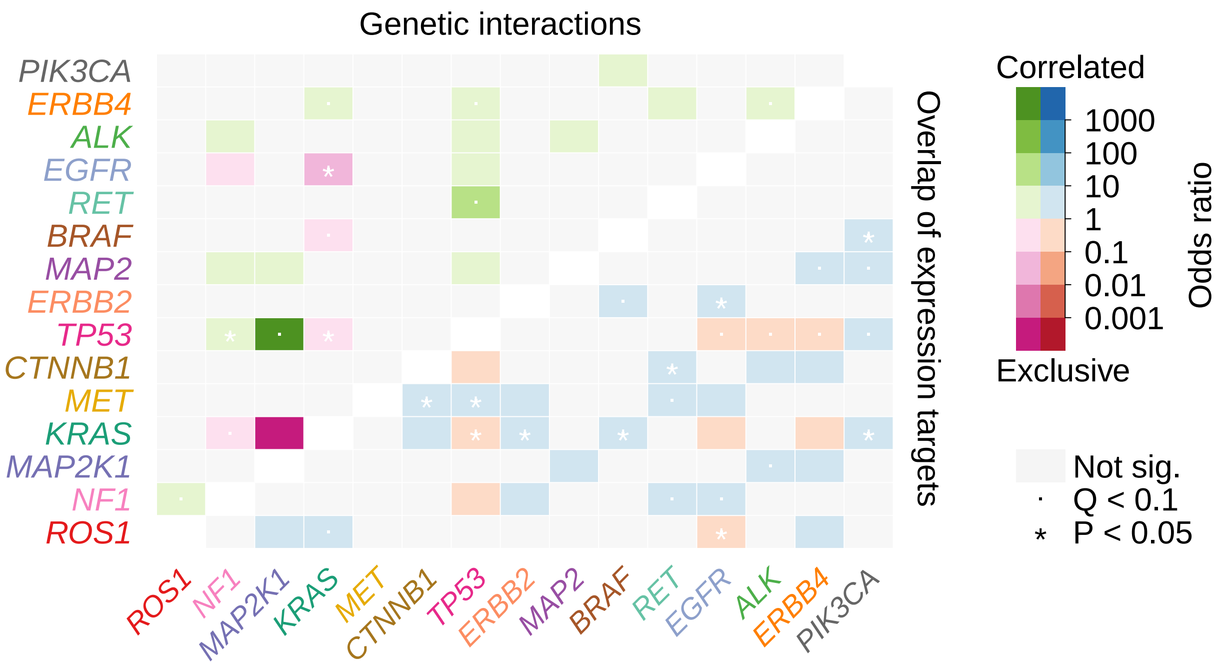

6 Principal components analysis

6.1. Explained variance by principal components

```
pca <- prcomp(t(geneExpr))
par(bty="n", mgp = c(2.5,.5,0), mar=c(3,4,1,2)+.1, tcl=-.25, las=1)
plot(pca$sdev^2/sum(pca$sdev^2), type="h", col=set1[1], xlab="",
     ylab=expression(paste("Explained variance ", Rgenetics^2)) , ylim=c(0,0.1), yaxs="i")
mtext(side=1, "Principal component", line=2)
c <- cumsum(pca$sdev^2)/sum(pca$sdev^2)* pca$sdev[1]^2/sum(pca$sdev^2)
lines(c  , type="s")
axis(4, at = pretty(c(0,1))* pca$sdev[1]^2/sum(pca$sdev^2), labels=pretty(c(0,1)))
legend("bottomright", col=c(set1[1],"black"), lty=1, c("Per PC","Cumulative"), bty="n")
lines(c(ncol(geneExpr),20,20),c(c[20],c[20],0), lty=3)
```

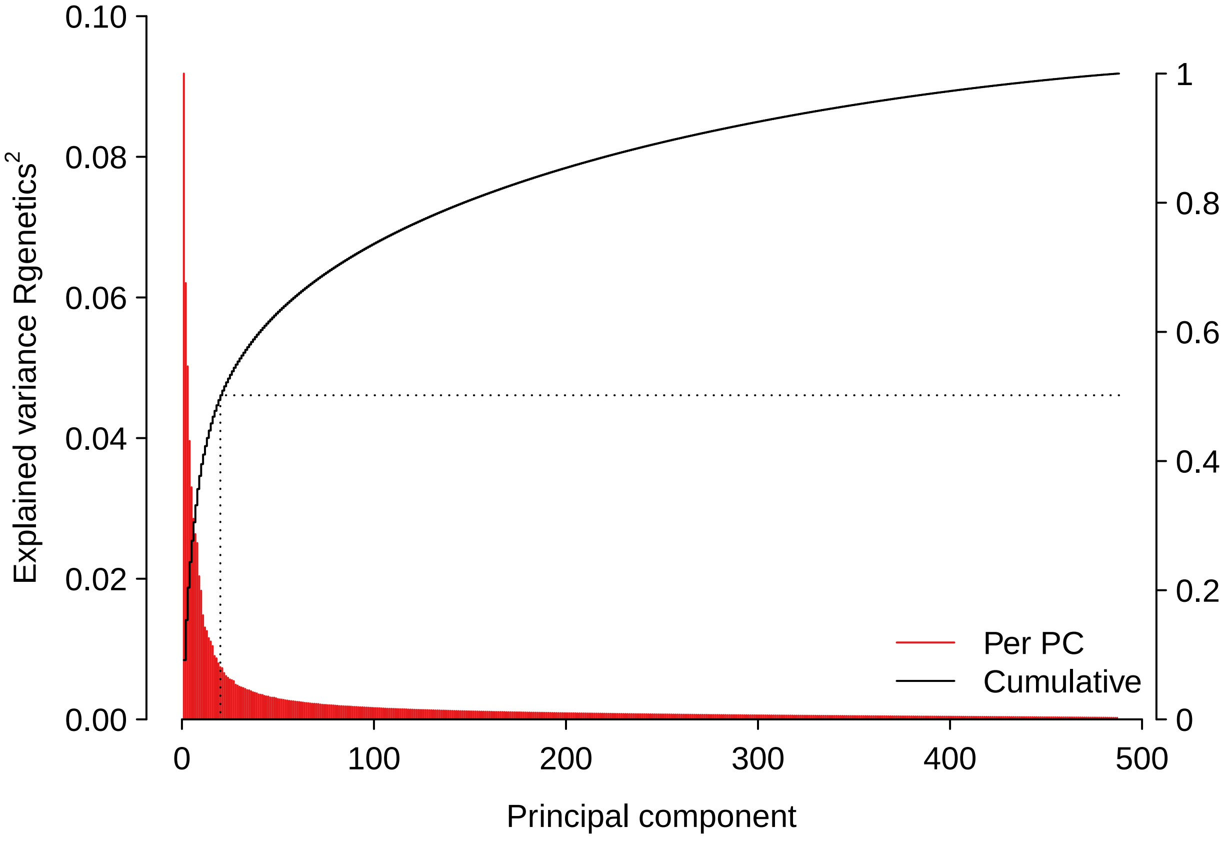

6.2 The first two PCs overlaid with the mutation

```
par(bty="n", mgp = c(0,0.5,0), mar=c(1,1,1.5,0)+.1, las=1, tcl=-.25,
    font.main=3, mfrow=c(4,5), xpd=NA)
i<-0
pointsize=0.75
for (geneId in colnames(design)[2:ncol(design)]){
  i<-i+1
  plot(pca$x[rownames(design),], cex=0.5,
       pch=NA,
       xlab=ifelse(i==1,"PC1",""), ylab=ifelse(i==1,"PC2",""),main=geneId,
       font.main=ifelse(grepl("[[:lower:]]",geneId),1,3),
       cex.main=1.33, cex.lab=1.2,xaxt="n", yaxt="n", ylim=c(-55,55))
  if(geneId != "age")
    w <- rownames(design)[design[,geneId] == 1]
  else
    w <- rownames(design)[which(design[,geneId] > median(design[,geneId], na.rm=TRUE))]
  points(pca$x[!rownames(pca$x) %in% w,],
         pch=ifelse(is.na(design[!rownames(pca$x) %in% w,geneId]),1,19),
         cex=pointsize, col="grey",
         lwd=0.5)
  points(pca$x[w,], pch=16, cex=pointsize, col=colMutations[i], lwd=0.05)
  u <- matrix(par("usr"), ncol=2)
  if(i==1){
    arrows(u[1,1],u[1,2], u[2,1],u[1,2],length=0.02)
```

```
    arrows(u[1,1],u[1,2], u[1,1],u[2,2],length=0.02)
  }
  text(u[2],u[4]*.8, labels=paste("n=",length(w), sep=""), bty="n", cex=1.2, pos=2)
}
plot.new()
legend("center",c("Mutant","Female","Age>median"), pch=c(19,19,19),
       col=c("black",colMutations[c("gender","age")]), bty="n",
       pt.cex=c(rep(0.8,3)),cex=1.2, pt.lwd=0.5)
```

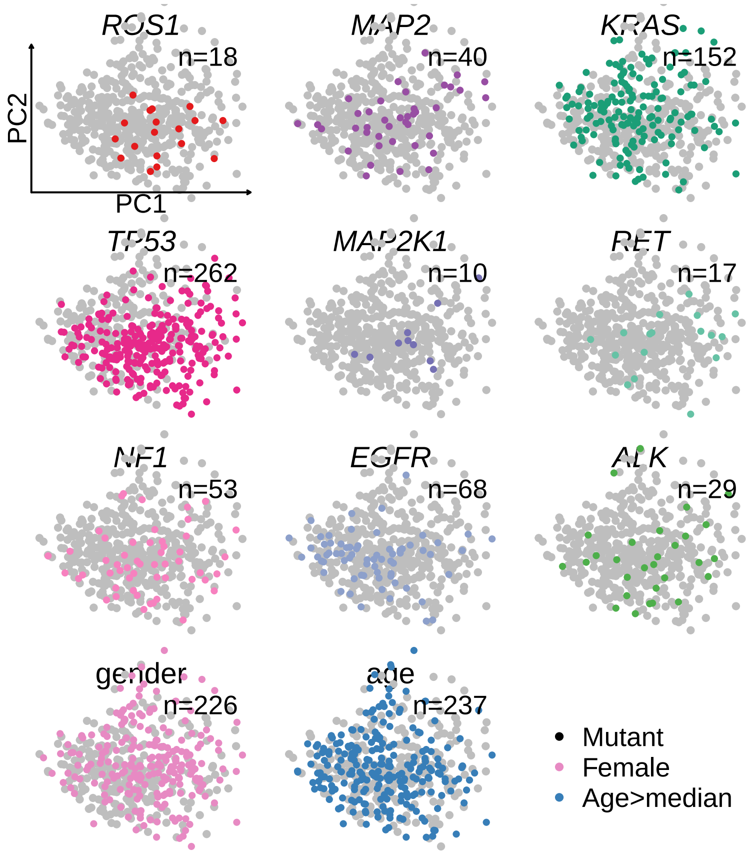

6.3 Enrichment of GO Terms in PC1&2 and write out

```
annot <- select(org.Hs.eg.db, rownames(glm), c("SYMBOL","GENENAME","CHR","CHRLOC"))
annot1=annot[!duplicated(annot$ENTREZID),1:5]
pcarot=signif(cbind(pca$rotation, 3))
pcarot1=pcarot[!duplicated(rownames(pcarot)),]
t <- cbind(annot1, pcarot1)
write.table(t[order(t[,"PC1"]),1:25], file=paste(Sys.Date(),"-PCA-rotation.txt",sep=""),
            sep="\t", row.names = FALSE, quote=FALSE)

#Enrichment of GO Terms in PC1&2
k = AnnotationDbi::as.list(org.Hs.egGO2EG)
k = k[sapply(k, length)>=10]
c <- pca$rotation[,1:2] *  sqrt(glm$s2.post)
n <- rownames(pca$rotation)
t = mclapply(k, function(p){
```

```r
  ids = n %in% p
  sapply(1:ncol(c), function(j){
    if(sum(ids)>2){
      t <- t.test(c[ids,j], c[!ids,j], alternative="two.sided")
      v <- var.test(c[ids,j], c[!ids,j], alternative="greater")
      c(t$p.value, -diff(t$estimate),
        v$p.value, v$estimate)
    }else
      rep(NA,4)
  })
}, mc.cores=1)
GO.pca = matrix(unlist(t), byrow = TRUE, nrow=length(t),
                dimnames=list(names(t),as.vector(outer(c("pval.t","shift","pval.F","var.odds"),
                                                       paste(colnames(c), sep=""), paste))))

Term(names(sort(GO.pca[p.adjust(GO.pca[,1],"BH")<0.1,2])[1:20]))
```

```
##                                                          GO:0003341
##                                                   "cilium movement"
##                                                          GO:0030881
##                                      "beta-2-microglobulin binding"
##                                                          GO:0036159
##                                         "inner dynein arm assembly"
##                                                          GO:0007585
##                                      "respiratory gaseous exchange"
##                                                          GO:0036158
##                                         "outer dynein arm assembly"
##                                                          GO:0005858
##                                           "axonemal dynein complex"
##                                                          GO:0042613
##                                      "MHC class II protein complex"
##                                                          GO:0019370
##                                  "leukotriene biosynthetic process"
##                                                          GO:0032395
##                                    "MHC class II receptor activity"
##                                                          GO:0031362
##                "anchored component of external side of plasma membrane"
##                                                          GO:0035082
##                                                 "axoneme assembly"
##                                                          GO:0005581
##                                                   "collagen trimer"
##                                                          GO:0060285
##                                    "cilium-dependent cell motility"
##                                                          GO:0030449
##                               "regulation of complement activation"
##                                                          GO:0070330
##                                               "aromatase activity"
##                                                          GO:0006958
##                          "complement activation, classical pathway"
##                                                          GO:0006956
##                                            "complement activation"
##                                                          GO:0086012
## "membrane depolarization during cardiac muscle cell action potential"
##                                                          GO:0005044
```

```
##                                          "scavenger receptor activity"
##                                                            GO:0071880
##  "adenylate cyclase-activating adrenergic receptor signaling pathway"
```

```r
Term(names(sort(GO.pca[p.adjust(GO.pca[,1],"BH")<0.1,2], decreasing = TRUE)[1:20]))
```

```
##                                            GO:0008608
## "attachment of spindle microtubules to kinetochore"
##                                            GO:0000780
##  "condensed nuclear chromosome, centromeric region"
##                                            GO:0000070
##              "mitotic sister chromatid segregation"
##                                            GO:0051310
##                       "metaphase plate congression"
##                                            GO:0007051
##                              "spindle organization"
##                                            GO:0043142
##     "single-stranded DNA-dependent ATPase activity"
##                                            GO:0003688
##                     "DNA replication origin binding"
##                                            GO:0050905
##                             "neuromuscular process"
##                                            GO:0006268
##         "DNA unwinding involved in DNA replication"
##                                            GO:0051382
##                              "kinetochore assembly"
##                                            GO:0051233
##                                   "spindle midzone"
##                                            GO:0006270
##                        "DNA replication initiation"
##                                                  <NA>
##                                                    NA
##                                            GO:0007019
##                      "microtubule depolymerization"
##                                            GO:0010369
##                                      "chromocenter"
##                                            GO:0007094
##               "mitotic spindle assembly checkpoint"
##                                            GO:0000775
##                    "chromosome, centromeric region"
##                                            GO:0009922
##                      "fatty acid elongase activity"
##                                            GO:0007062
##                         "sister chromatid cohesion"
##                                            GO:0007076
##                   "mitotic chromosome condensation"
```

```r
Term(names(sort(GO.pca[p.adjust(GO.pca[,5],"BH")<0.1,6])[1:20]))
```

```
##                                            GO:0032395
##                      "MHC class II receptor activity"
##                                            GO:0035987
##                     "endodermal cell differentiation"
##                                            GO:0048247
##                              "lymphocyte chemotaxis"
```

```
##                                                  GO:0030199
##                                "collagen fibril organization"
##                                                  GO:0042613
##                                "MHC class II protein complex"
##                                                  GO:0008009
##                                          "chemokine activity"
##                                                  GO:0019864
##                                                 "IgG binding"
##                                                  GO:0048020
##                              "CCR chemokine receptor binding"
##                                                  GO:0030574
##                                  "collagen catabolic process"
##                                                  GO:0002523
##     "leukocyte migration involved in inflammatory response"
##                                                  GO:0070098
##                        "chemokine-mediated signaling pathway"
##                                                  GO:0032700
##          "negative regulation of interleukin-17 production"
##                                                  GO:0031424
##                                             "keratinization"
##                                                  GO:0050840
##                                "extracellular matrix binding"
##                                                  GO:0045086
## "positive regulation of interleukin-2 biosynthetic process"
##                                                  GO:0010759
##              "positive regulation of macrophage chemotaxis"
##                                                  GO:2000406
##                   "positive regulation of T cell migration"
##                                                  GO:0018149
##                                     "peptide cross-linking"
##                                                  GO:0050718
##       "positive regulation of interleukin-1 beta secretion"
##                                                  GO:0090026
##                "positive regulation of monocyte chemotaxis"
```

```
write.table(data.frame(GO.id=rownames(GO.pca),
                       GO.Term = Term(names(k)) ,
                       n.genes = sapply(k, length),
                       GO.pca),
            file=paste(Sys.Date(),"-PCA-GO.txt", sep=""), sep="\t", row.names=FALSE, quote=FALSE)
```

7 Clinical regression

7.1 pre-processing of Clinical data

```
cli_N=cli[rownames(cli) %in% rownames(design),]
cli_N1=as.data.frame(cli_N[,c("Pathology_Updated","pathologic_stage",
                              "tobacco_smoking_history","gender",
                              "age_at_initial_pathologic_diagnosis")])
cli_N1$tobacco_smoking_history=ifelse(cli_N1$tobacco_smoking_history>1,1,0)
#0 is  Non-smoker,1 is smoker
Trait=cli_N1
Trait1=matrix(c(rep(0,nrow(cli_N1)*length(na.omit(unique(Trait[,1]))))),nrow = nrow(Trait))
rownames(Trait1)=rownames(Trait)
```

```
colnames(Trait1)=as.character(na.omit(unique(Trait[,1])))
for (i in 1:ncol(Trait1)) {
  Trait1[Trait[,1]==colnames(Trait1)[i],i]=1
  Trait1[is.na(Trait[,1]),i]=NA
  }
Pathology=Trait1[,colSums(Trait1,na.rm = T)>5]
Pathology=Pathology[,c(5,6,1,2,3,4)]

cli_N=cli[rownames(cli) %in% rownames(design),]
cli_N1=as.data.frame(cli_N[,c("Pathology_Updated","pathologic_stage",
                              "tobacco_smoking_history","gender",
                              "age_at_initial_pathologic_diagnosis")])
cli_N1$tobacco_smoking_history=ifelse(cli_N1$tobacco_smoking_history>1,1,0)
#0 is  Non-smoker,1 is smoker
Trait=cli_N1
Trait[,1][Trait[,1]=="Colloid adenoca"|Trait[,1]=="NSCLC, favor Adeno"|
            Trait[,1]=="Adenocarcinoma, NOS"]=NA
Trait1=matrix(c(rep(0,nrow(cli_N1)*length(na.omit(unique(Trait[,1]))))),nrow = nrow(Trait))
rownames(Trait1)=rownames(Trait)
colnames(Trait1)=as.character(na.omit(unique(Trait[,1])))
for (i in 1:ncol(Trait1)) {
  Trait1[Trait[,1]==colnames(Trait1)[i],i]=1
  Trait1[is.na(Trait[,1]),i]=NA
}
Pathology=Trait1[,colSums(Trait1,na.rm = T)>5]
Pathology=Pathology[,c(5,6,1,2,3,4)]
#table(Pathology[,1])

Trait=cli_N1
Trait$pathologic_stage[Trait$pathologic_stage=="Stage IA"|
                         Trait$pathologic_stage=="Stage IB"]="Stage I"
Trait$pathologic_stage[Trait$pathologic_stage=="Stage IIA"|
                         Trait$pathologic_stage=="Stage IIB"]="Stage II"
Trait$pathologic_stage[Trait$pathologic_stage=="Stage IIIA"|
                         Trait$pathologic_stage=="Stage IIIB"]="Stage III"
Trait1=matrix(c(rep(0,nrow(cli_N1)*length(na.omit(unique(Trait[,2]))))),nrow = nrow(Trait))
rownames(Trait1)=rownames(Trait)
colnames(Trait1)=as.character(na.omit(unique(Trait[,2])))
#NA  is set to 1
for (i in 1:ncol(Trait1)) {
  Trait1[Trait[,2]==colnames(Trait1)[i],i]=1
  Trait1[is.na(Trait[,2]),i]=NA
}
stage=as.data.frame(Trait1[,colSums(Trait1,na.rm = T)>5])
stage[which(stage$`[Discrepancy]`==1),]=NA
stage=stage[,c(2,4,3,1)]
Y=cbind(stage,Pathology,cli_N1[,3:ncol(cli_N1)])
#age
class(Y$age_at_initial_pathologic_diagnosis)
```

```
## [1] "integer"
```

```
colnames(Y)[ncol(Y)]="age"
#gender
```

```
Y$gender[Y$gender=="MALE"]=as.numeric(1)
Y$gender[Y$gender=="FEMALE"]=as.numeric(0)
Y$gender=as.numeric(Y$gender)
geneExpr=geneExpr[,rownames(Y)]
pca <- prcomp(t(geneExpr))
subs=design[rownames(Y),2:(ncol(design)-2)]
```

7.2 Define variable categories

```
Z <-  list()
Z$expression = scale(pca$x[row.names(pca$x) %in% rownames(Y),1:20])
Z$genetics = scale(subs+0, scale=FALSE)
mat=match(rownames(Z$genetics),rownames(Z$expression))
Z$expression=Z$expression[mat,]
mat1=match(rownames(Z$expression),rownames(Y))
Y=Y[mat1,]
Z$geneticsExpression = cbind(Z$genetics,Z$expression)
Z$Pathology = scale(Pathology)
Z$demographics = scale(design[,c("gender", "age")])
Z$clinical = cbind(Z$Pathology,Z$demographics)
Z$expressionClinical = cbind(Z$expression, Z$clinical)
Z$geneticsClinical = cbind(Z$genetics, Z$clinical)
Z$geneticsExpressionClinical = cbind(Z$geneticsExpression, Z$clinical)
Z$all = cbind(Z$genetics, Z$expression, Z$clinical)

#Save the working environment
#save(list =ls(all=TRUE), file="LUAD.RData")
```

7.3 Compute LASSO regression and plot

```
load(file="LUAD.RData")
X <- scale(Z$geneticsExpression, scale=TRUE)
for(j in 1:ncol(X))
  X[is.na(X[,j]),j] <- mean(X[,j], na.rm=TRUE)
set.seed(40)
Y=data.frame(Y)
clinModels = lapply(Y, function(y){
  if (class(y) %in% c("numeric","integer")){
    if(all(y %in% c(0,1,NA)))
      cv.glmnet(X[!is.na(y),], na.omit(y), family = "binomial", alpha=1, standardize=FALSE, nfolds=5)
    else if(all(y %in% c(0,20,NA)))
      cv.glmnet(X[!is.na(y),], na.omit(y), family = "poisson", alpha=1, standardize=FALSE, nfolds=5)
    else
      cv.glmnet(X[!is.na(y),], na.omit(y), family = "gaussian", alpha=1, standardize=FALSE, nfolds=5)
  }
  else if (class(y)=="factor")
    cv.glmnet(X[!is.na(y),], na.omit(y), family="multinomial",  alpha=1, standardize=FALSE, nfolds=5)
})
#plot
i = 1
n <- colnames(Z$geneticsExpression)
annot <- 1 + grepl("^[A-Z]",n) + grepl("PC",n)
```

```r
names(annot) <- n
for(m in clinModels){
  #pdf(paste0("lasso",names(clinModels)[i],".pdf"),width = 10)
  par(bty="n", mgp = c(2.5,.5,0), mar=c(5,5,2,5)+.1, las=2, tcl=-.25)
  plotcvnet(m, Z$geneticsExpression, main=names(clinModels)[i],  col0="black",
            cex=1, simple.annot = annot, col=set1[c(3,2,4)])
  i = i+1
  legend("topright", col=c(set1[c(1,3)],"black")[c(1,3,2)],
         c(expression(paste("Explained variance ",Rgenetics^2)),
           expression(paste("Lasso penalty ",lambda)),
           expression(paste("Model coefficient ", beta))),
         box.lty=0, bg="#FFFFFF33", pch=c(NA,NA,19),
         lty=c(1,1,NA), cex=.8, pt.cex = 1)
  #dev.off()
  }
```

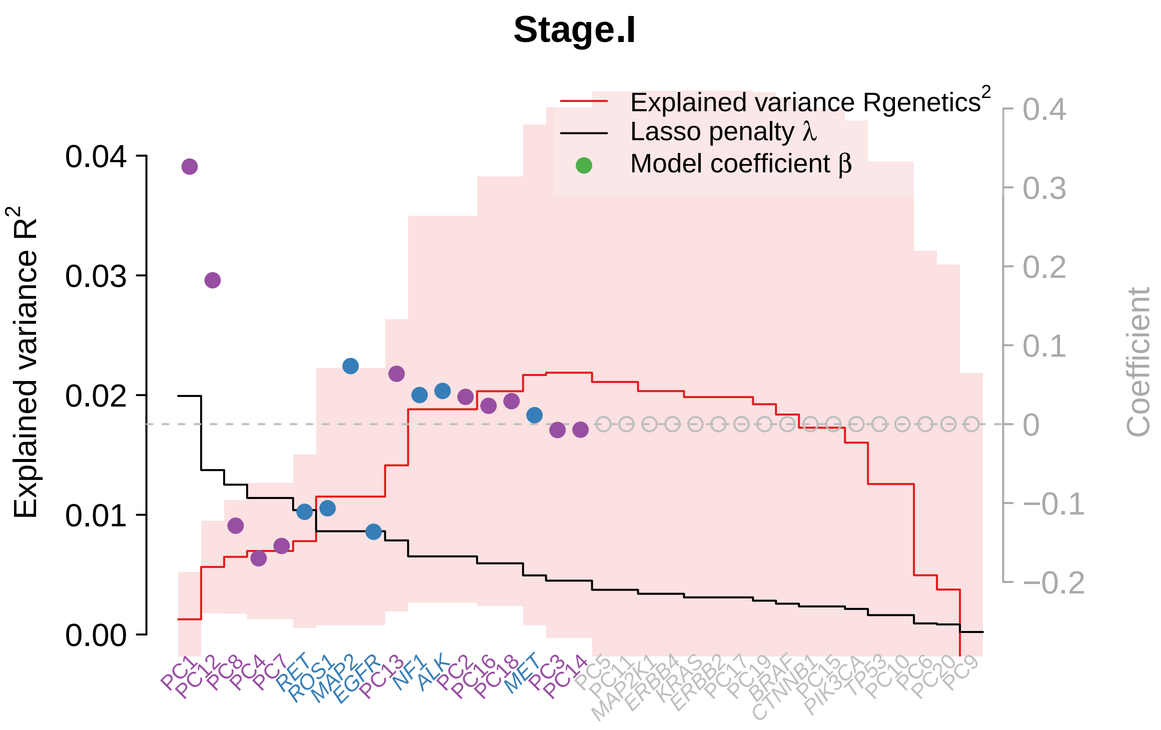

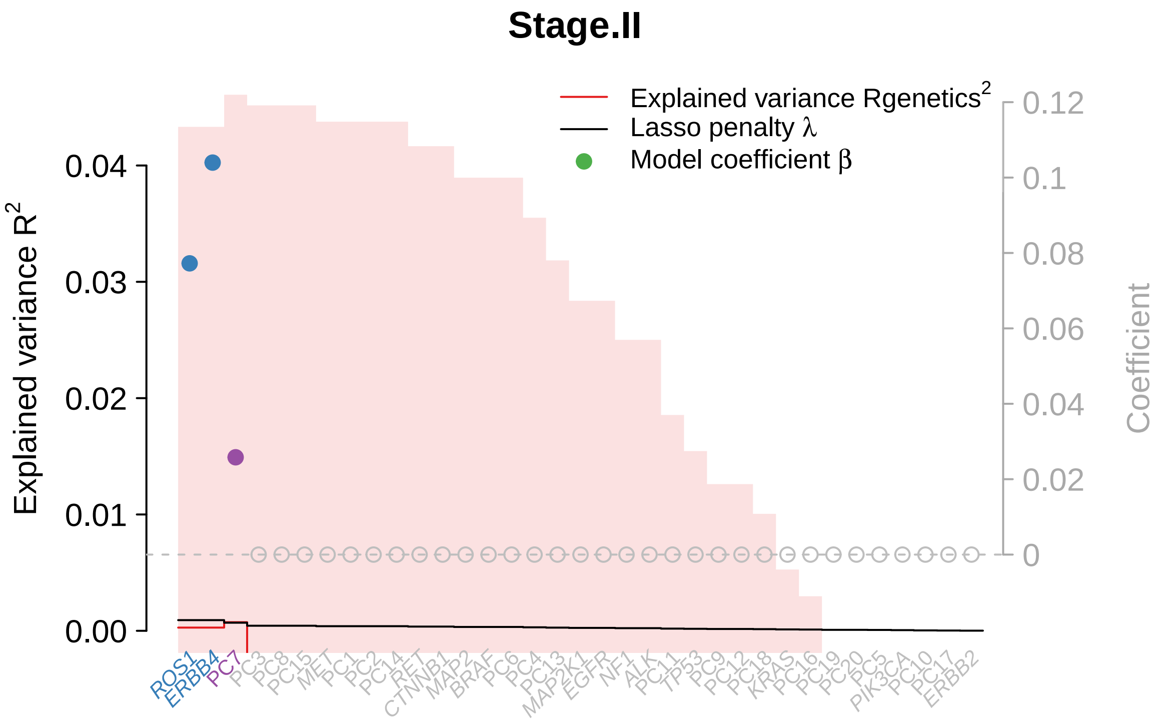

Stage.II
Explained variance Rgenetics2
Lasso penalty λ
Model coefficient β
Explained variance R2
Coefficient
0.04
0.03
0.02
0.01
0.00
0.12
0.1
0.08
0.06
0.04
0.02
0
ROS1
ERBB4
PC7
PC3
PC8
PC15
MET
PC1
PC2
PC14
RET
CTNNB1
MAP2
BRAF
PC6
PC4
PC13
MAP2K1
EGFR
NF1
ALK
PC11
TP53
PC9
PC12
PC18
KRAS
PC16
PC19
PC20
PC5
PIK3CA
PC10
PC17
ERBB2

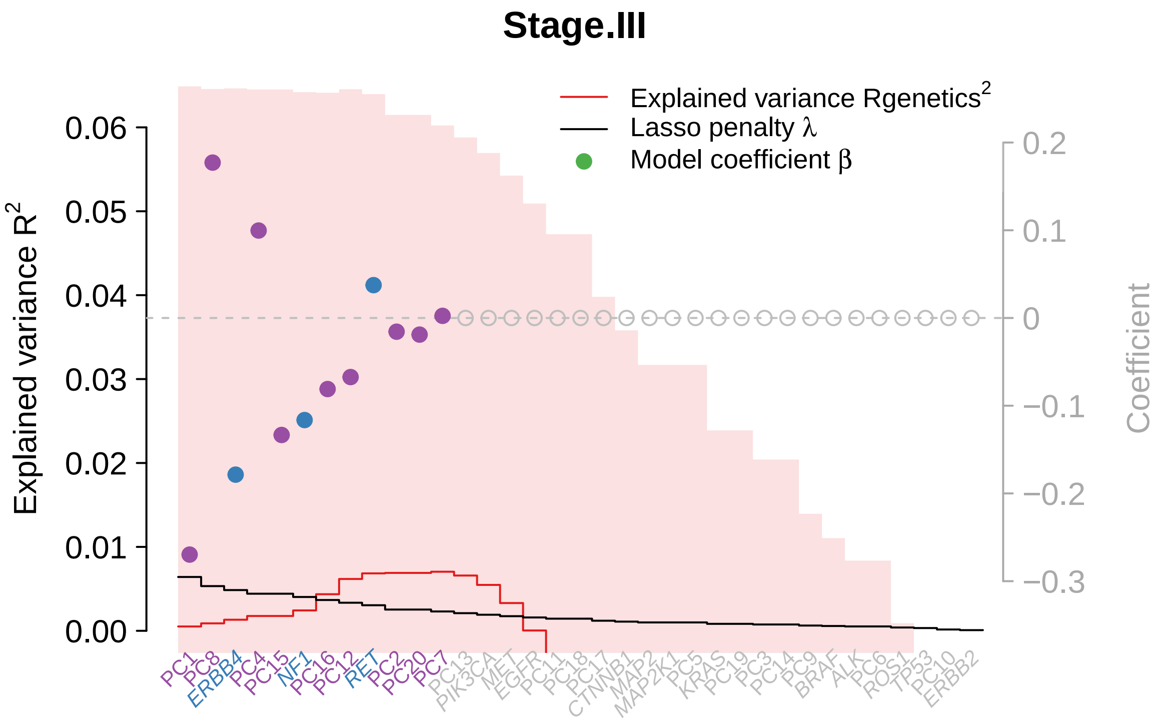

Stage.III
Explained variance Rgenetics^2
Lasso penalty λ
Model coefficient β
Explained variance R^2
Coefficient
0.06
0.05
0.04
0.03
0.02
0.01
0.00
0.2
0.1
0
−0.1
−0.2
−0.3
PC1
PC8
ERBB4
PC4
PC15
NF1
PC16
PC12
RET
PC2
PC20
PC7
PC13
PIK3CA
MET
EGFR
PC11
PC18
PC17
CTNNB1
MAP2
MAP2K1
PC5
KRAS
PC19
PC3
PC14
PC9
BRAF
ALK
PC6
ROS1
TP53
PC10
ERBB2

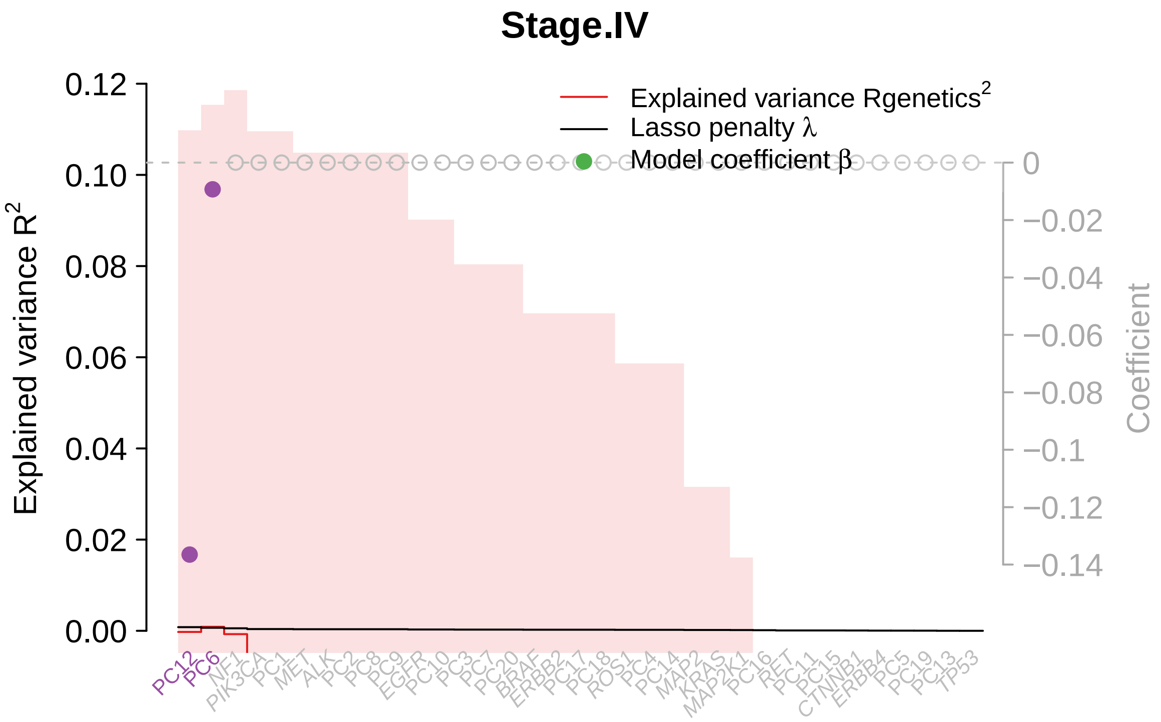
Stage.IV
Explained variance Rgenetics^2
Lasso penalty λ
Model coefficient β
Explained variance R^2
Coefficient
0.12
0.10
0.08
0.06
0.04
0.02
0.00
0
−0.02
−0.04
−0.06
−0.08
−0.1
−0.12
−0.14
PC12
PC6
NF1
PIK3CA
PC1
MET
ALK
PC2
PC8
PC9
EGFR
PC10
PC3
PC7
PC20
BRAF
ERBB2
PC17
PC18
ROS1
PC4
PC14
MAP2
KRAS
MAP2K1
PC16
RET
PC11
PC15
CTNNB1
ERBB4
PC5
PC19
PC13
TP53

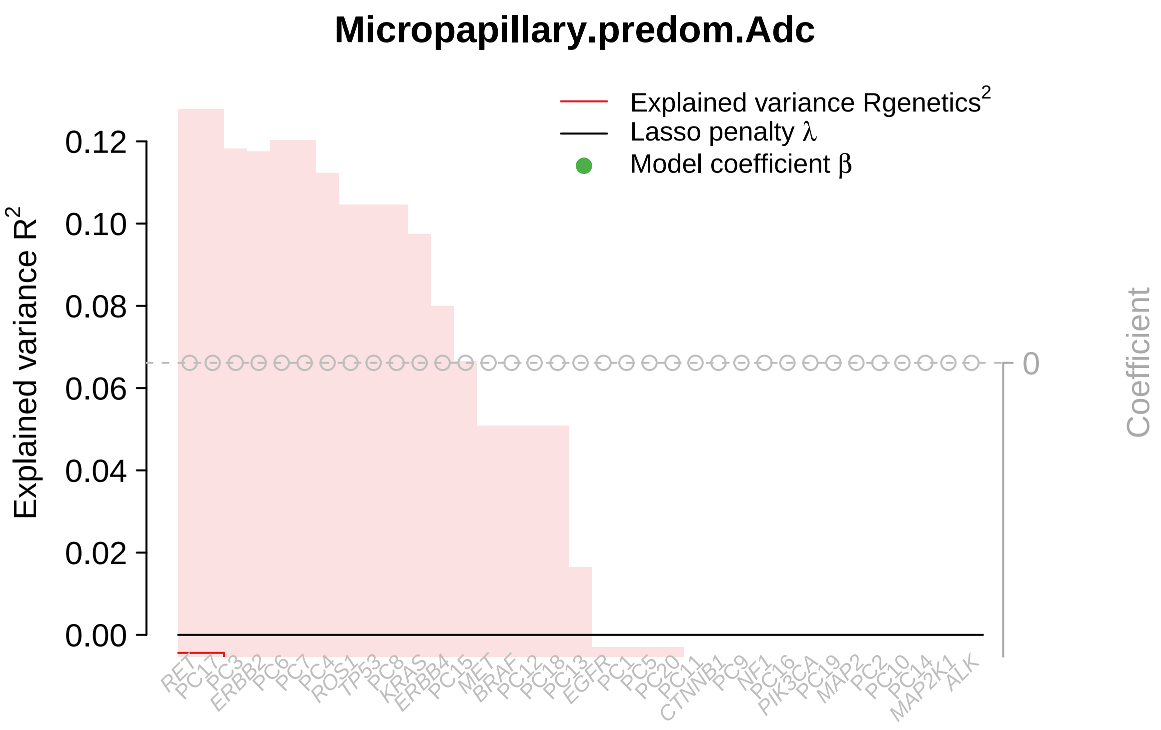

Micropapillary.predom.Adc
Explained variance Rgenetics^2
Lasso penalty λ
Model coefficient β
Explained variance R^2
0.12
0.10
0.08
0.06
0.04
0.02
0.00
0
Coefficient
RET
PC17
PC3
ERBB2
PC6
PC7
PC4
ROS1
TP53
PC8
KRAS
ERBB4
PC15
MET
BRAF
PC12
PC18
PC13
EGFR
PC1
PC5
PC20
PC11
CTNNB1
PC9
NF1
PC16
PIK3CA
PC19
MAP2
PC2
PC10
PC14
MAP2K1
ALK

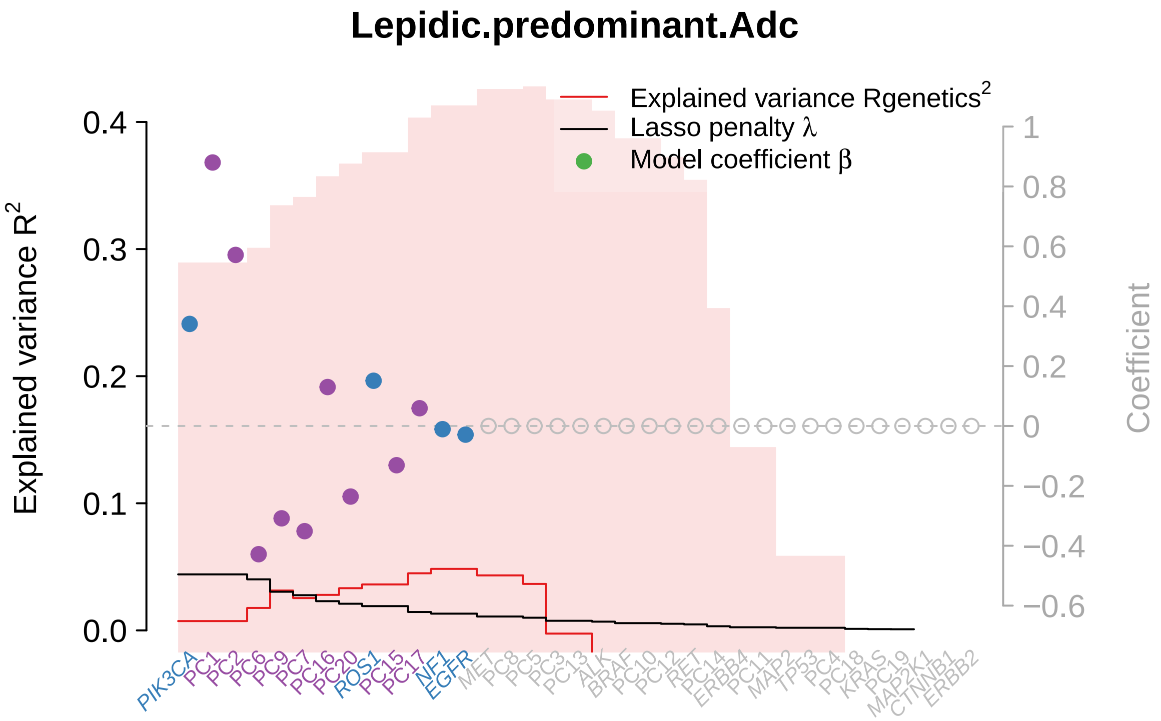

Lepidic.predominant.Adc
Explained variance Rgenetics2
Lasso penalty λ
Model coefficient β
Explained variance R2
Coefficient
0.4
0.3
0.2
0.1
0.0
1
0.8
0.6
0.4
0.2
0
−0.2
−0.4
−0.6
PIK3CA
PC1
PC2
PC6
PC9
PC7
PC16
PC20
ROS1
PC15
PC17
NF1
EGFR
MET
PC8
PC5
PC3
PC13
ALK
BRAF
PC10
PC12
RET
PC14
ERBB4
PC11
MAP2
TP53
PC4
PC18
KRAS
PC19
MAP2K1
CTNNB1
ERBB2

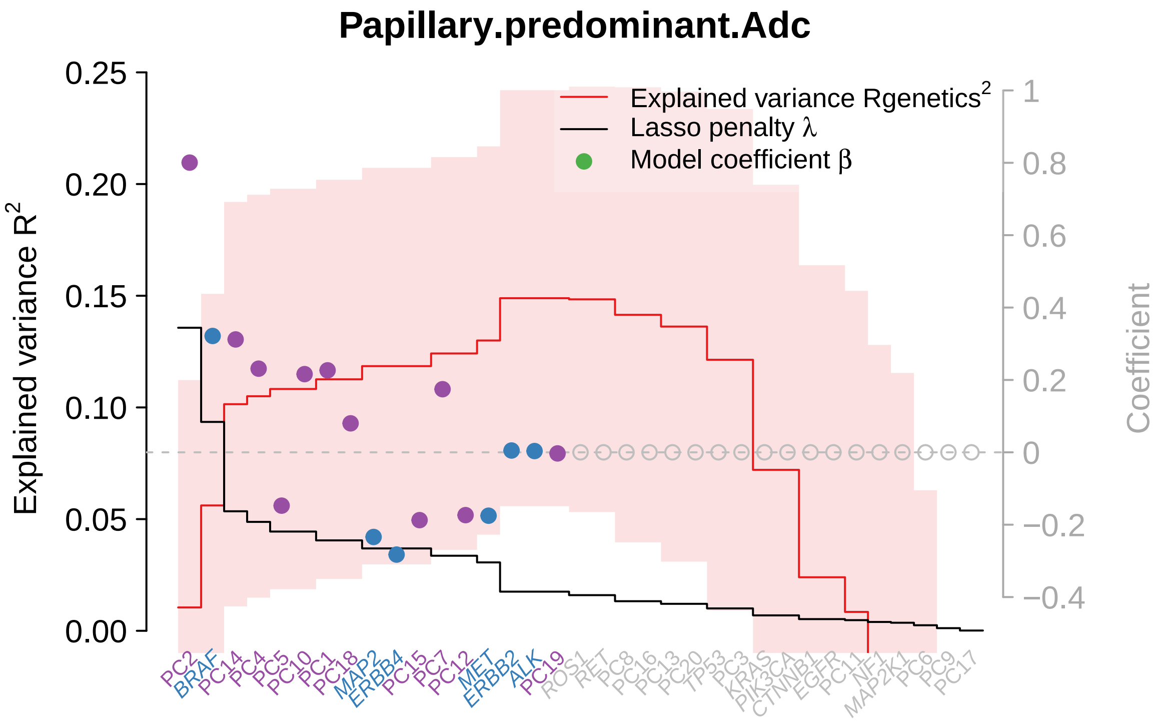

Papillary.predominant.Adc
Explained variance R2
Coefficient
Explained variance Rgenetics2
Lasso penalty λ
Model coefficient β
0.25
0.20
0.15
0.10
0.05
0.00
1
0.8
0.6
0.4
0.2
0
−0.2
−0.4
PC2
BRAF
PC14
PC4
PC5
PC10
PC1
PC18
MAP2
ERBB4
PC15
PC7
PC12
MET
ERBB2
ALK
PC19
ROS1
RET
PC8
PC16
PC13
PC20
TP53
PC3
KRAS
PIK3CA
CTNNB1
EGFR
PC11
NF1
MAP2K1
PC6
PC9
PC17

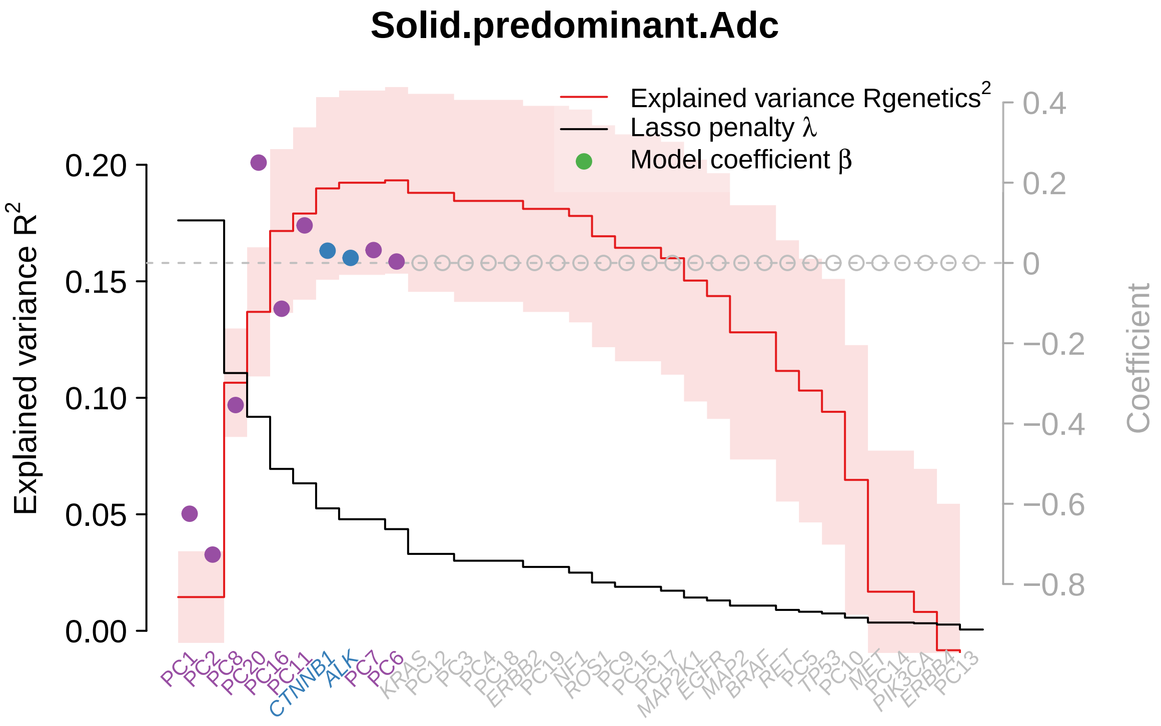

Solid.predominant.Adc
Explained variance Rgenetics²
Lasso penalty λ
Model coefficient β
Explained variance R²
Coefficient
0.20
0.15
0.10
0.05
0.00
0.4
0.2
0
−0.2
−0.4
−0.6
−0.8
PC1
PC2
PC8
PC20
PC16
PC11
CTNNB1
ALK
PC7
PC6
KRAS
PC12
PC3
PC4
PC18
ERBB2
PC19
NF1
ROS1
PC9
PC15
PC17
MAP2K1
EGFR
MAP2
BRAF
RET
PC5
TP53
PC10
MET
PC14
PIK3CA
ERBB4
PC13

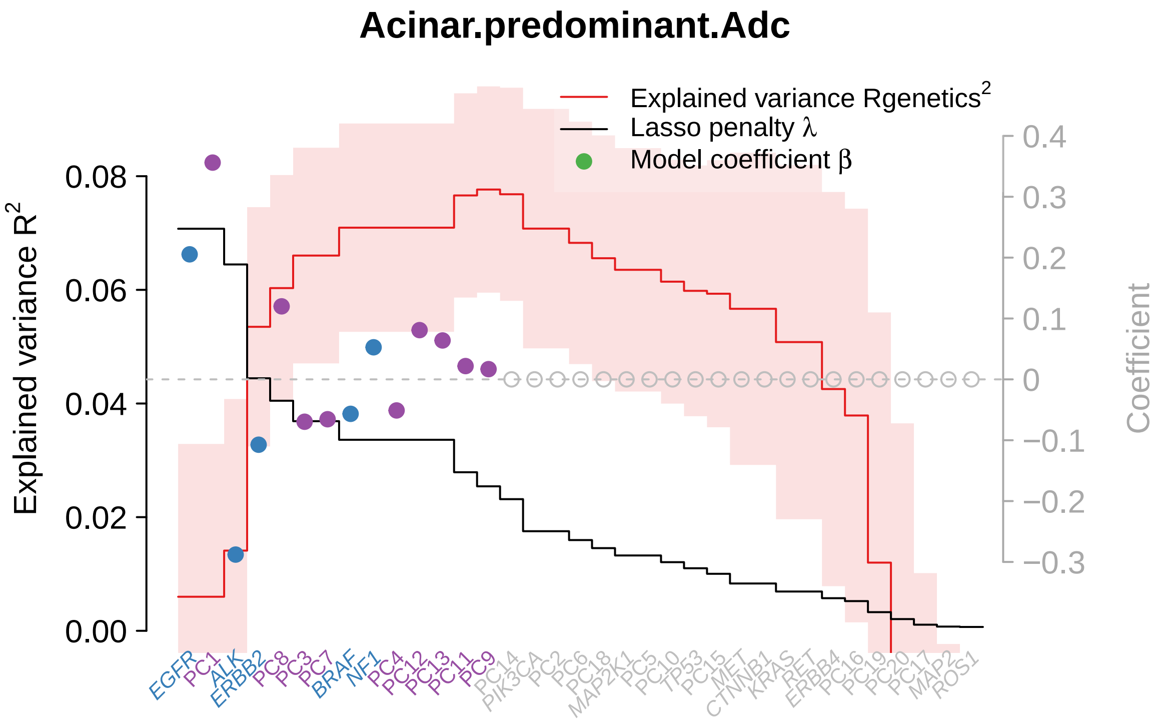

Acinar.predominant.Adc
Explained variance Rgenetics^2
Lasso penalty λ
Model coefficient β
Explained variance R^2
Coefficient
0.00
0.02
0.04
0.06
0.08
0.4
0.3
0.2
0.1
0
−0.1
−0.2
−0.3
EGFR
PC1
ALK
ERBB2
PC8
PC3
PC7
BRAF
NF1
PC4
PC12
PC13
PC11
PC9
PC14
PIK3CA
PC2
PC6
PC18
MAP2K1
PC5
PC10
TP53
PC15
MET
CTNNB1
KRAS
RET
ERBB4
PC16
PC19
PC20
PC17
MAP2
ROS1

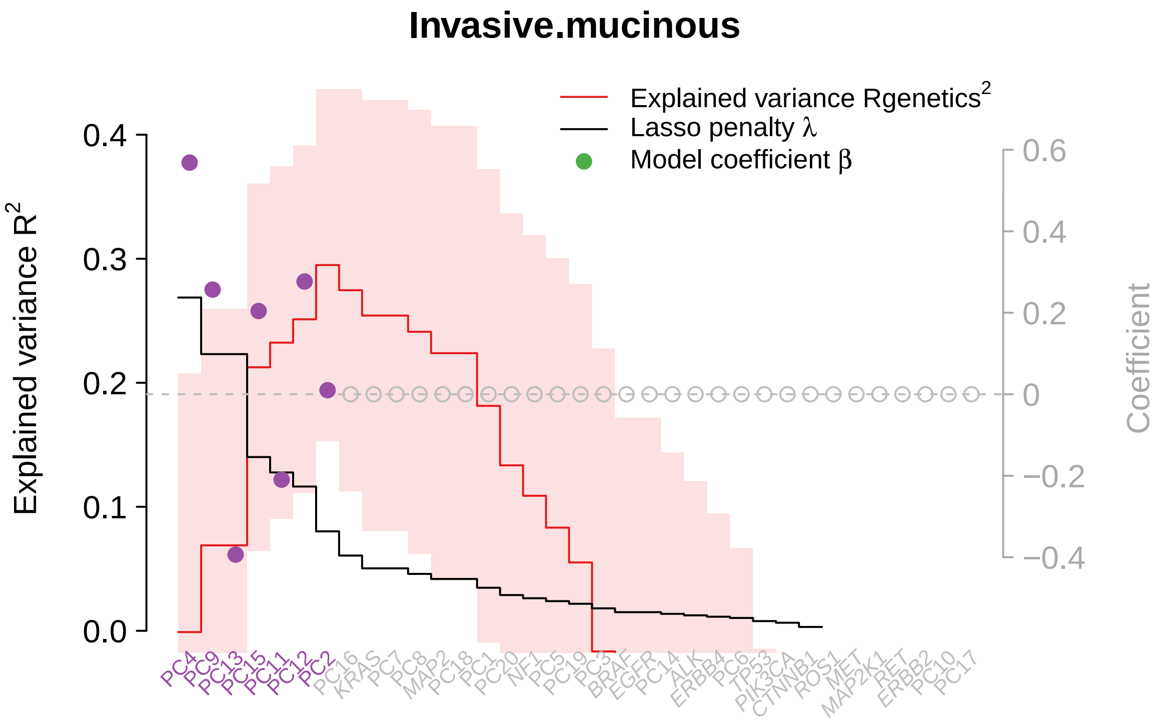
Invasive.mucinous
Explained variance Rgenetics^2
Lasso penalty λ
Model coefficient β
Explained variance R^2
Coefficient
0.0
0.1
0.2
0.3
0.4
0.6
0.4
0.2
0
−0.2
−0.4
PC4
PC9
PC13
PC15
PC11
PC12
PC2
PC16
KRAS
PC7
PC8
MAP2
PC18
PC1
PC20
NF1
PC5
PC19
PC3
BRAF
EGFR
PC14
ALK
ERBB4
PC6
TP53
PIK3CA
CTNNB1
ROS1
MET
MAP2K1
RET
ERBB2
PC10
PC17

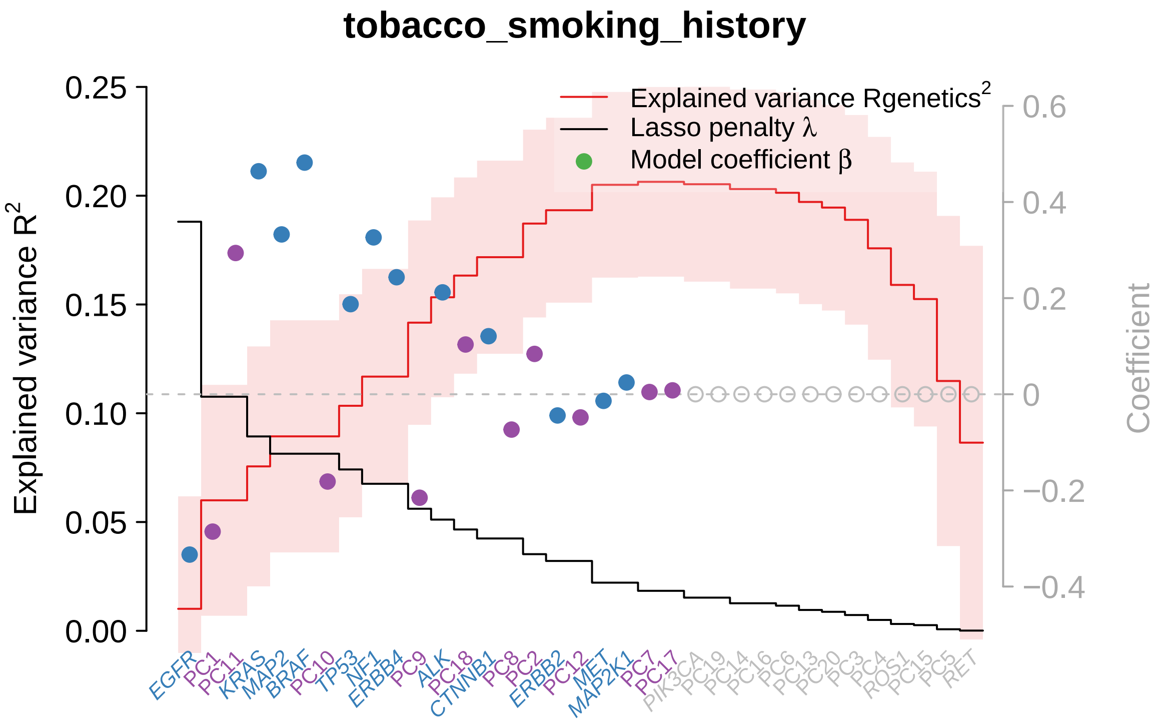

tobacco_smoking_history
Explained variance Rgenetics^2
Lasso penalty λ
Model coefficient β
Explained variance R^2
Coefficient
0.25
0.20
0.15
0.10
0.05
0.00
0.6
0.4
0.2
0
−0.2
−0.4
EGFR PC1 PC11 KRAS MAP2 BRAF PC10 TP53 NF1 ERBB4 PC9 ALK PC18 CTNNB1 PC8 PC2 ERBB2 PC12 MET MAP2K1 PC7 PC17 PIK3CA PC19 PC14 PC16 PC6 PC13 PC20 PC3 PC4 ROS1 PC15 PC5 RET

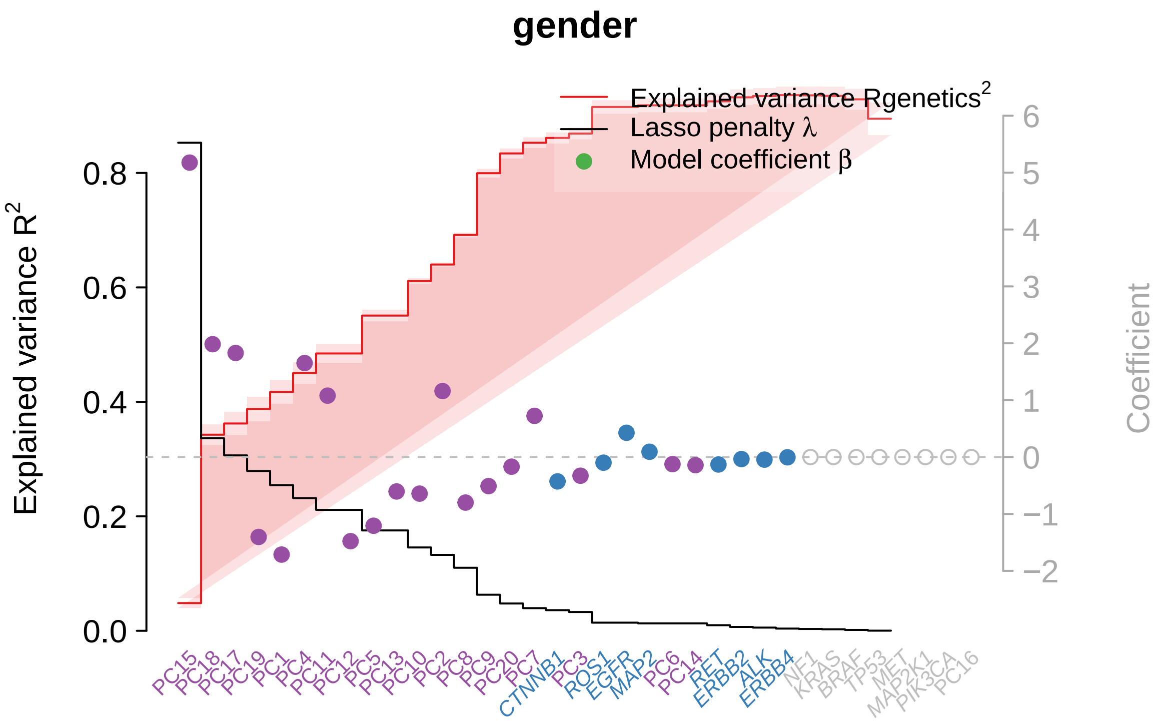
gender
Explained variance Rgenetics^2
Lasso penalty λ
Model coefficient β
Explained variance R^2
0.0
0.2
0.4
0.6
0.8
Coefficient
6
5
4
3
2
1
0
−1
−2
PC15
PC18
PC17
PC19
PC1
PC4
PC11
PC12
PC5
PC13
PC10
PC2
PC8
PC9
PC20
PC7
CTNNB1
PC3
ROS1
EGFR
MAP2
PC6
PC14
RET
ERBB2
ALK
ERBB4
NF1
KRAS
BRAF
TP53
MET
MAP2K1
PIK3CA
PC16

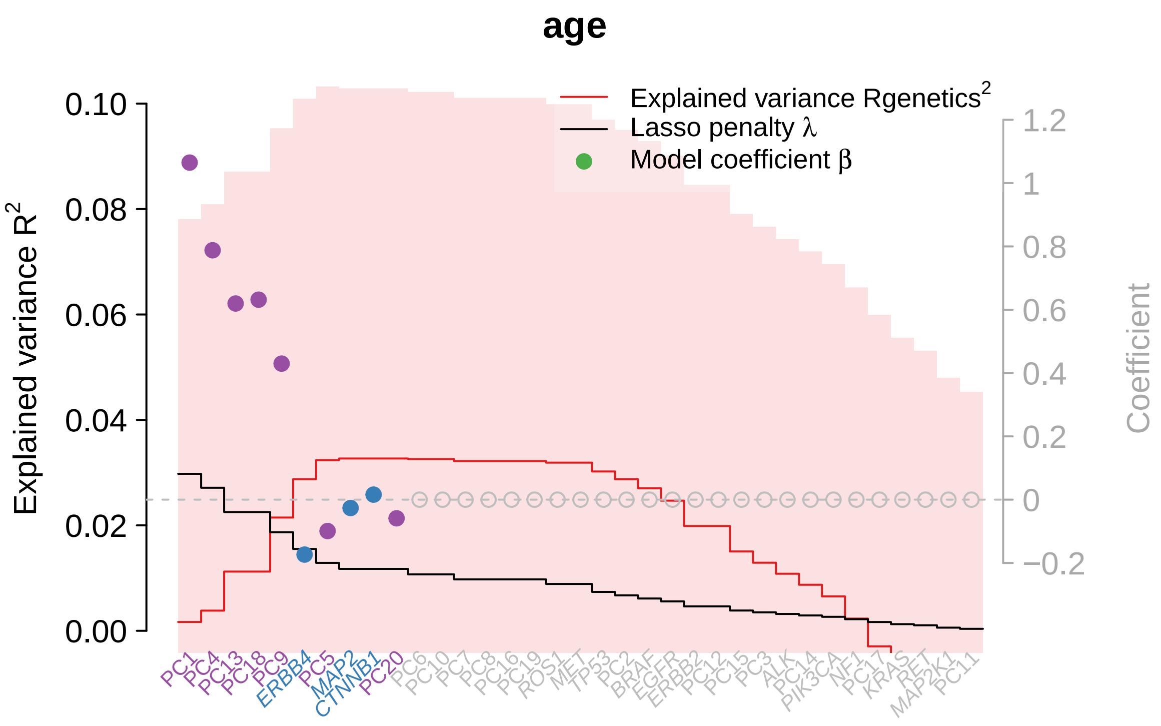

7.4 Summary of the model for histologic subtypes, pathological stages, and demographics.

```
j <- 0
z <- sapply(clinModels,function(x){
  j <<- j+1
  w <- which.min(x$cvm)
  c <- x$glmnet.fit$beta[,w]
  yj <- sapply(c("genetics","cytogenetics","expression"), function(i){
    w <- colnames(Z[[i]])
    X[,w] %*% c[w]
  })
  cj <- rowSums(cov(yj))
  y <- Y[,j] - x$glmnet.fit$a0[w]
  covj <- colMeans((y-mean(y))*(yj - rep(colMeans(yj), each=nrow(yj))))
  r2 <- cj
  R2 <- 1 - x$cvm[w]/x$cvm[1]
  c(c, NA,  r2/sum(r2), R2=R2)
})
m <- nrow(z) #ncol(Z$geneticsCytogeneticsExpression)
z[1:m,] <- pmin(z[1:m,],1)
z[1:m,] <- pmax(z[1:m,],-.999)
z[z==0] <- NA

r <- sapply(clinModels, function(x)
  rank(apply(x$glmnet.fit$beta,1, function(y)
```

```
    which(y!=0)[1]), ties="min"))
s <- sapply(clinModels, function(x) {
  w = which.min(x$cvm)
  w <- rev(which(x$cvm[1:w] > x$cvup[w]))[1] +1
  if(!is.na(w))
    sum(x$glmnet.fit$beta[,w]!=0)
  else
    0
})

p <- sapply(clinModels, function(x) {w <- which.min(x$cvm); (1 - x$cvup[w]/x$cvm[1]) > 0 })
#R2 <- z[nrow(z) - 3:0,]
#R2[is.na(R2)] <- 0
z <- z[-(nrow(z) - 0:4),]
z <- z[,ncol(z):1]
r <- r[,ncol(r):1]
layout(matrix(c(1,2),1,2), c(6.5,.75), 2, T)
par(bty="n", mgp = c(3,.5,0), mar=c(4,11,2,0)+.1, las=1, tcl=-.25, cex=1)
w <- TRUE
image(y=1:ncol(z)-.5, x=1:nrow(z), z[,w], breaks=c(-2,seq(-1,1,l=51)),
      col=c("grey",colorRampPalette(brewer.pal(9,"RdYlBu"))(50)),
      xaxt="n", yaxt="n", xlab="", ylab="", ylim=c(0,14))
rotatedLabel(y0=rep(0.5,nrow(z)), labels=sub("_","/",rownames(z)), x0=1:nrow(z),
             font=c(rep(3,18),rep(1,nrow(z)-18)),
             col = set1[c(3,2,4)][annot], cex=0.9)
abline(v=c(15.5), lwd=0.3)
mtext(side=2, line=.2, text=colnames(z), las=2, at=1:ncol(z)-.5)
text(y=rep(1:ncol(z)-.5, each=nrow(r)), x=rep(1:nrow(r), ncol(z)),
     r[,w] * (0!=(z[1:nrow(r),w])), cex=0.66,
     font=ifelse(r <= rep(s[ncol(r):1], each=nrow(r)), 2,1))
points(y=rep(1:ncol(z)-.5, each=nrow(r)), x=rep(1:nrow(r), ncol(z)),
       pch=ifelse(is.na(z) | z==0, ".",NA))
mtext(side=1, at=8, "Genetics", col=set1[2], line=2.5 )
mtext(side=1, at=28, "Expression", col=set1[4], line=2.5 )
mtext(side=3, "Model coefficients", at = 12, line=-0.4)
clip(-10,30,0,16)
image(y=dim(z)[2] + c(0.5,1.5), x=1+ 1:7 , matrix(seq(-0.99,1,l=7), ncol=1),
      breaks=c(-2,seq(-1,1,l=51)),
      col=c("grey",colorRampPalette(brewer.pal(9,"RdYlBu"))(50)),
        xaxt="n", yaxt="n", xlab="", ylab="", add=TRUE)
text(y=dim(z)[2]+1, x=c(1,9), c(-1,1))
points(y=11,x=5, pch=".")
rect(19.5,10.5+3,20.5,11.5+3, lwd=0.5)
text(20,11+3,1, cex=0.66)
text(21, 11+3, "LASSO rank", pos=4)
```

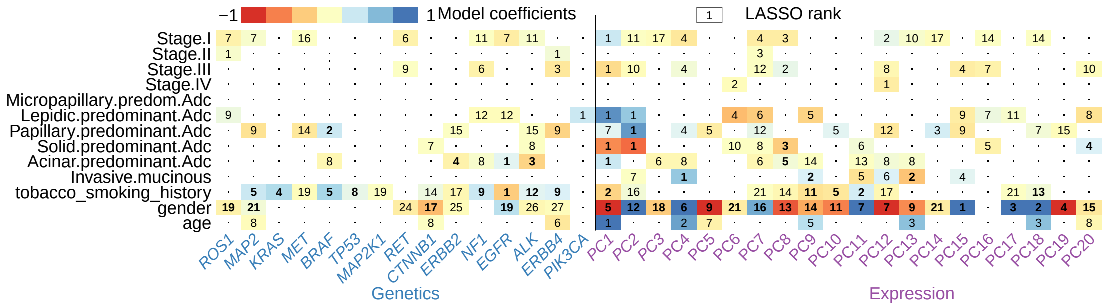

8 Survival analysis

OS(overall survival)

```
cli=cli[row.names(Y),]
amlFreeSurvival <- Surv(time=cli$OS.time, event=cli$OS)
amlFreeSurvival[,1] <- amlFreeSurvival[,1] /30 # Convert to months

ecoxph <- function(X, surv, tol=1e-3, max.iter=50){
  if(class(X)=="data.frame")
    X = as.matrix(X)
  beta0 = rep(0,ncol(X))
  beta1 = rep(1,ncol(X))
  sigma2 = 1
  iter = 1
  while(max(abs(beta1-beta0))>tol& iter < max.iter){
    fit = coxph(surv ~ ridge(X, theta=1/sigma2, scale=FALSE))
    sigma2 = (1 + sum((fit$coefficients-mean(fit$coefficients))^2))/(ncol(X))
    beta0 = beta1
    beta1 = fit$coefficients
    #cat(beta1,"\n")
    #cat(sigma,"\n")
    iter = iter+1
  }
  fit$sigma2 = sigma2
  names(fit$coefficients) = colnames(X)
  return(fit)
}

for(i in names(Z)){
  for(j in 1:ncol(Z[[i]])){
    Z[[i]][is.na(Z[[i]][,j]),j] <- mean(Z[[i]][,j], na.rm=TRUE)
  }
}

#Use five-fold cross validation
set.seed(112)
concordanceCV = data.frame()
```

```r
cv_ix = sample(1:5, length(rownames(Y)), replace=TRUE)
for(i in unique(cv_ix)){
  c = lapply(Z, function(x) ecoxph(x[cv_ix!=i,], amlFreeSurvival[cv_ix!=i] ))
  p = mapply(function(x,y) as.matrix(x[cv_ix==i,]) %*% coef(y), Z, c)
  concordanceCV = rbind(concordanceCV, apply(-p,2, rcorr.cens, amlFreeSurvival[cv_ix==i])[1,])
}

colnames(concordanceCV) = names(Z)
colMeans(concordanceCV)
```

```
##                  expression                    genetics
##                   0.6684888                   0.5279056
##          geneticsExpression                demographics
##                   0.6651879                   0.5379271
##                   Pathology                    clinical
##                   0.5525810                   0.5662948
##          expressionClinical            geneticsClinical
##                   0.6721067                   0.5652394
## geneticsExpressionClinical                         all
##                   0.6698046                   0.6698046
```

```r
concordanceCV
```

```
##   expression  genetics geneticsExpression demographics Pathology  clinical
## 1  0.5815739 0.4600128          0.5777351    0.4955214 0.5595010 0.5626999
## 2  0.7101158 0.4803724          0.6864620    0.5908405 0.6077001 0.6396578
## 3  0.6532385 0.4684044          0.6532385    0.5363349 0.5770142 0.5825434
## 4  0.6834634 0.6071682          0.6891412    0.5638751 0.4858055 0.5134847
## 5  0.7140523 0.6235703          0.7193627    0.5030637 0.5328840 0.5330882
##   expressionClinical geneticsClinical geneticsExpressionClinical       all
## 1          0.5834933        0.5102367                  0.5796545 0.5796545
## 2          0.7282335        0.5377453                  0.7010569 0.7010569
## 3          0.6698262        0.5936019                  0.6753555 0.6753555
## 4          0.6763662        0.5812633                  0.6784954 0.6784954
## 5          0.7026144        0.6033497                  0.7144608 0.7144608
```

```r
stage <- cli[row.names(Y),"pathologic_stage"]
stage[stage=="Stage IA"|stage=="Stage IB"|stage=="Stage I"]=1
stage[stage=="Stage IIA"|stage=="Stage IIB"|stage=="Stage IIIA"]=1
stage[stage=="Stage IIIB"]=2
stage[stage=="Stage IV"]=2
stage[stage=="[Discrepancy]"]=NA
stage=as.numeric(stage)
hIpss <- rcorr.cens(-stage,amlFreeSurvival)[1]
#plot
par(bty="n", mgp = c(2,.33,0), mar=c(5,3,1,1)+3.9, las=1, tcl=-.25, xpd=NA)
h <- concordanceCV[,c("genetics","expression","Pathology","demographics","all")]
v <- sapply(h, sd)/sqrt(5)
colnames(h) <- ""
b <- barplot(c(colMeans(h),hIpss), border=NA, col=paste(c(set1[c(2,3,4,1,5,7)],"#BBBBBB"),"88", sep=""),
             las=2, ylim=c(0,0.7), ylab="Harrel's C", names.arg=rep("",6))
points(rep(b[-6], each=5) + seq(-.1,.1,l=5), unlist(h), pch=16, cex=.5, col="darkgrey")
segments(x0=b[-6], y0=colMeans(h)-v, y1=colMeans(h)+v)
rotatedLabel(b, rep(0,6), c("Genetics","Expression","Pathology","Demographics","All","Stage"))
```

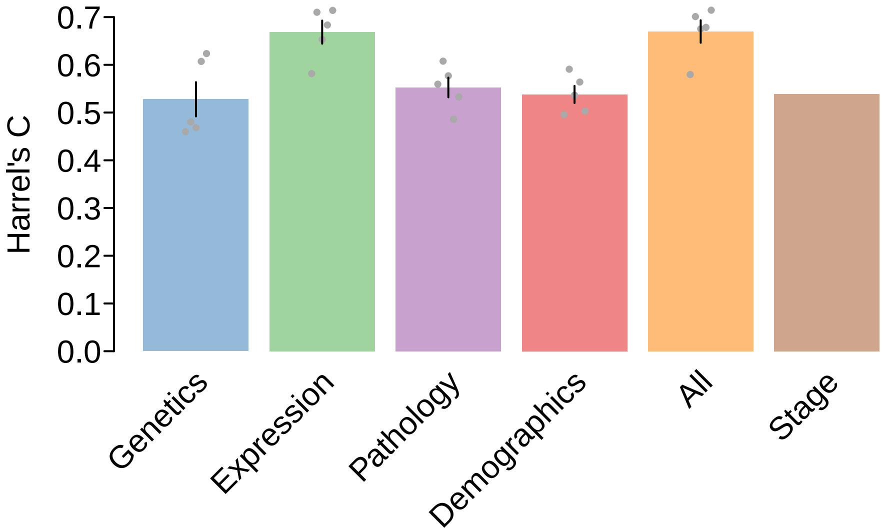

Risk contributions(OS)

Fit single model

```
X <-  data.frame(
  Genetics = Z$genetics,
  Expression= Z$expression,
  Pathology = Z$Pathology,
  Demographics =  Z$demographics
)

groups <- sub("\\..+","",colnames(X))
model <- ecoxph(X, amlFreeSurvival)
index <- TRUE
r <- sapply(unique(groups[index]), function(x) {
  ix <- groups[index] == x
  as.matrix(X[,index][,ix, drop=FALSE]) %*% coef(model)[ix] #+ fullModel$sumX[,x] * fullModel$mu[x]
})

c <- cov(r, use="complete")
x <-  colSums(c)/sum(c)#diag(c / sum(diag(c))) #
x <- x - sum(x[x < 0])
col0 <- c(paste(set1, "88", sep="")[c(3,2,4,1,5)], "grey")
pie(x, col=col0, border=NA, labels = paste(names(x), " (",round(100*x),"%)", sep=""))
polygon(cos(seq(0,2*pi,l=100))*.5, sin(seq(0,2*pi,l=100))*.5, col="white", border=NA)
```

```
C <- rcorr.cens(-rowSums(r), amlFreeSurvival)[1]
polygon(c(sin(seq(0,2*pi *C,l=100) +(1-C)*pi)*.4,0), c(cos(seq(0,2*pi *C,l=100)+(1-C)*pi)*.4,0),
        col="grey", border=NA)
text(0,-0.1, paste("C=",round(C,2), sep=""), col="white", pos=1)
```

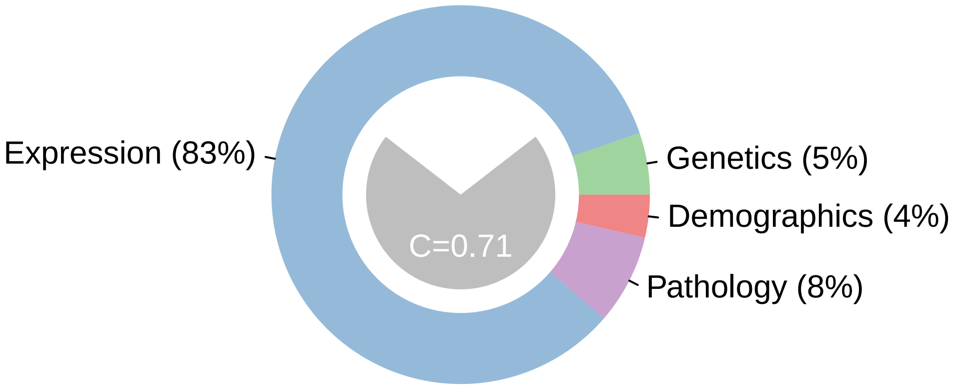

Five-fold cross validation

```
C <- NULL
y <- NULL
set.seed(112)
cv_ix = sample(1:5,length(rownames(Y)), replace=TRUE)
index <- TRUE
for(i in 1:5){
  fit = ecoxph(X[cv_ix!=i,index],amlFreeSurvival[cv_ix!=i], tol=1e-6)
  c = coef(fit)
  C = c(C,rcorr.cens(-as.matrix(X[cv_ix==i,index][,groups!="Nuisance"]) %*% c[groups!="Nuisance"] ,
                    amlFreeSurvival[cv_ix==i])[1])
  r <- sapply(unique(groups[index]), function(x) {
    ix <- groups[index] == x
    as.matrix(X[cv_ix!=i,index][,ix, drop=FALSE]) %*% coef(fit)[ix]
    #+ fullModel$sumX[,x] * fullModel$mu[x]
  })

  c <- cov(r, use="complete")
  x <-  colSums(c)/sum(c)#diag(c / sum(diag(c))) #
  y <- rbind(y,x)
```

```
}

meanC <- mean(C)
x <- colMeans(y)
x <- colSums(c)/sum(c)
x <- x - sum(x[x<0])
pi <- base::pi


par(bty="n", mgp = c(2,.33,0), mar=c(1,3,1,3)+.1, las=2, tcl=-.25)
pie(x, col=col0, border=NA, labels = paste(names(x), " (",round(100*x),"%)", sep=""),
    radius=.8, init.angle=179)
title(main="Survival risk contributions", font.main=1, cex.main=1)
polygon(cos(seq(0,2*pi,l=100))*.5, sin(seq(0,2*pi,l=100))*.5, col="white", border=NA)
polygon(c(sin(seq(0,2*pi *meanC,l=100) +(1-meanC)*pi)*.4,0),
        c(cos(seq(0,2*pi *meanC,l=100)+(1-meanC)*pi)*.4,0),
        col="grey", border=NA)
text(0,0, paste("C=",round(meanC,2), sep=""), col="white", pos=1)
```

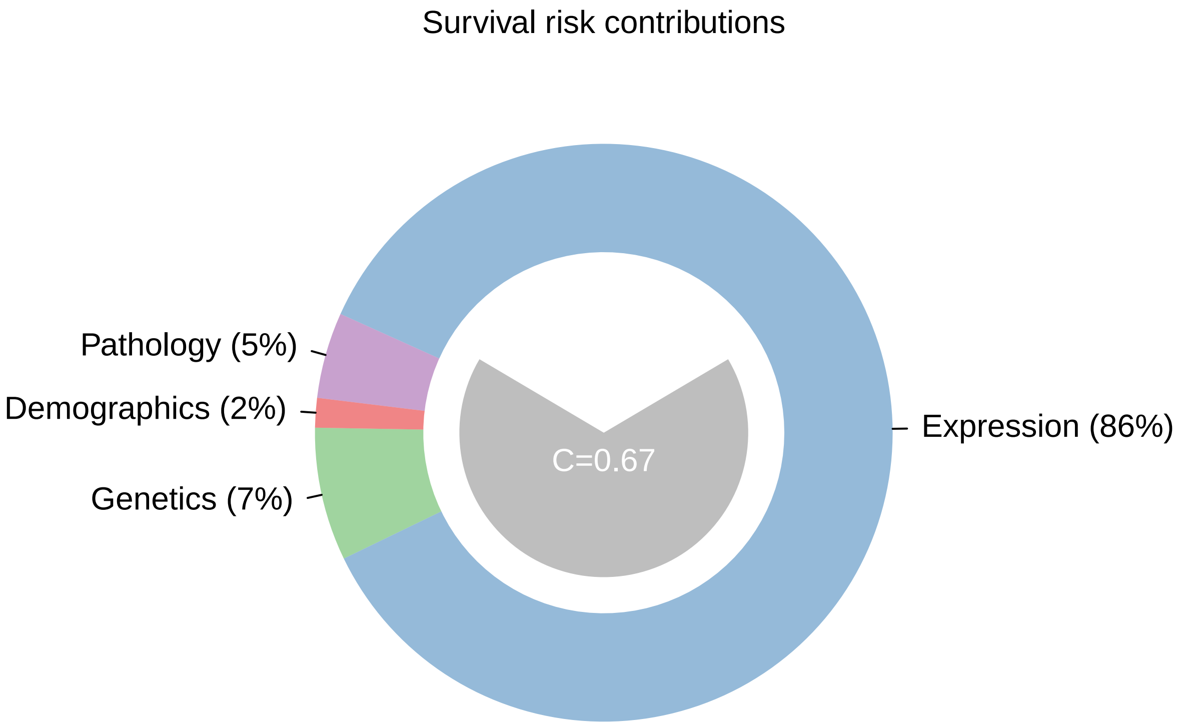

Kaplan-Meier plots of risk terciles

```
j <- 0
for(x in Z){
  j <- j +1
  #pdf(paste0("survival",capitalize(names(Z)[j]),".pdf"))
```

```
par(bty="n", mgp = c(1.9,.33,0), mar=c(2.3,3,2,2.1)+.1, las=1, tcl=-.25)
c = sapply(1:5, function(i) coef(ecoxph(x[cv_ix!=i,],amlFreeSurvival[cv_ix!=i])))
r = as.matrix(x) %*% rowMeans(c)
plot(survfit(amlFreeSurvival ~ cut(r, quantile(r+runif(length(r), -0.01,0.01),
                                       seq(0,1,l=4), na.rm=TRUE), right=FALSE)),
     col = set1[3:1], xlab="", ylab="Recurrence-free Survival")
title(xlab="Months", line=1.5)
mtext(capitalize(names(Z)[j]), font=2, at=100, side=3, line=0.5)
par(xpd=TRUE)
legend(x=200, y=1.0, bty="n", "Risk", cex=0.9, text.font=2)
legend(x=200, y=0.9, bty="n", text.col = set1[3:1], c("Low","Intermediate","High"), cex=0.9)
text(x=0, y=0.05, paste("C =", round(colMeans(concordanceCV)[j],2)), cex=1, pos=4)
#dev.off()
}
```

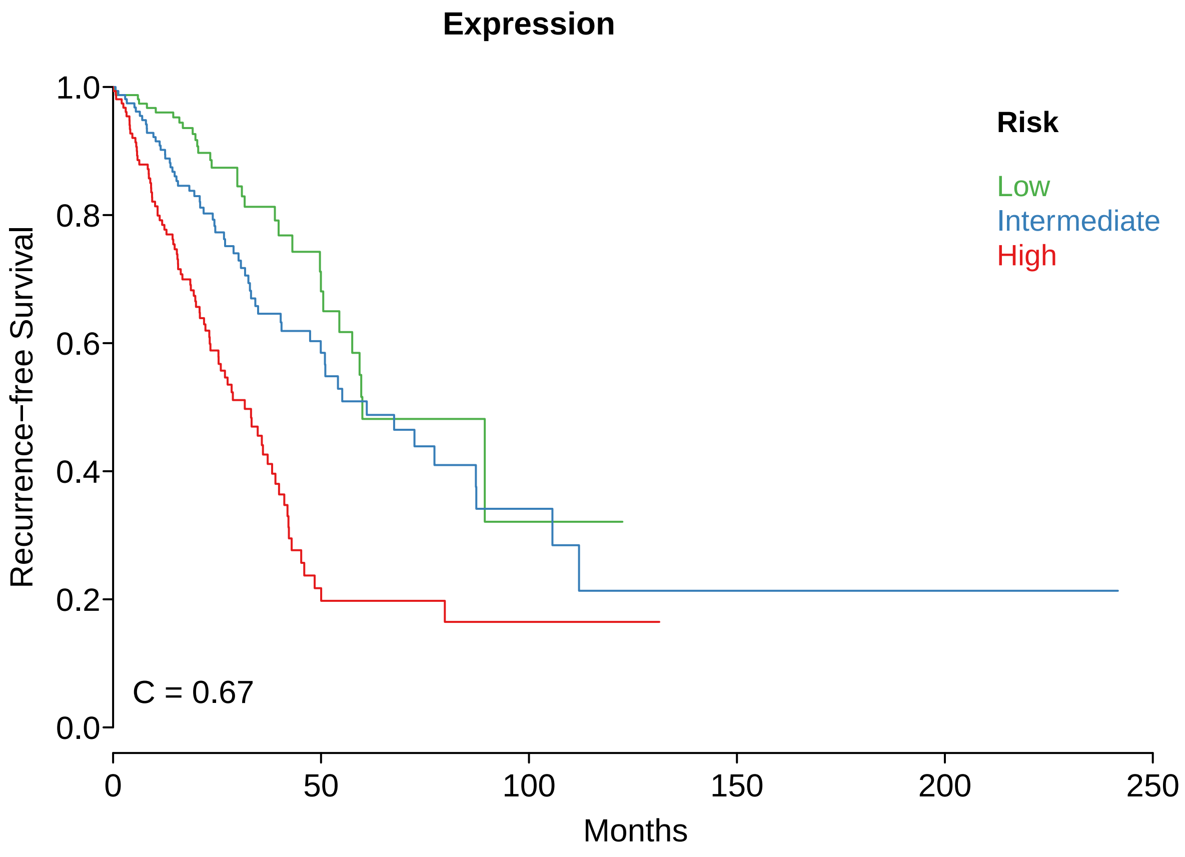

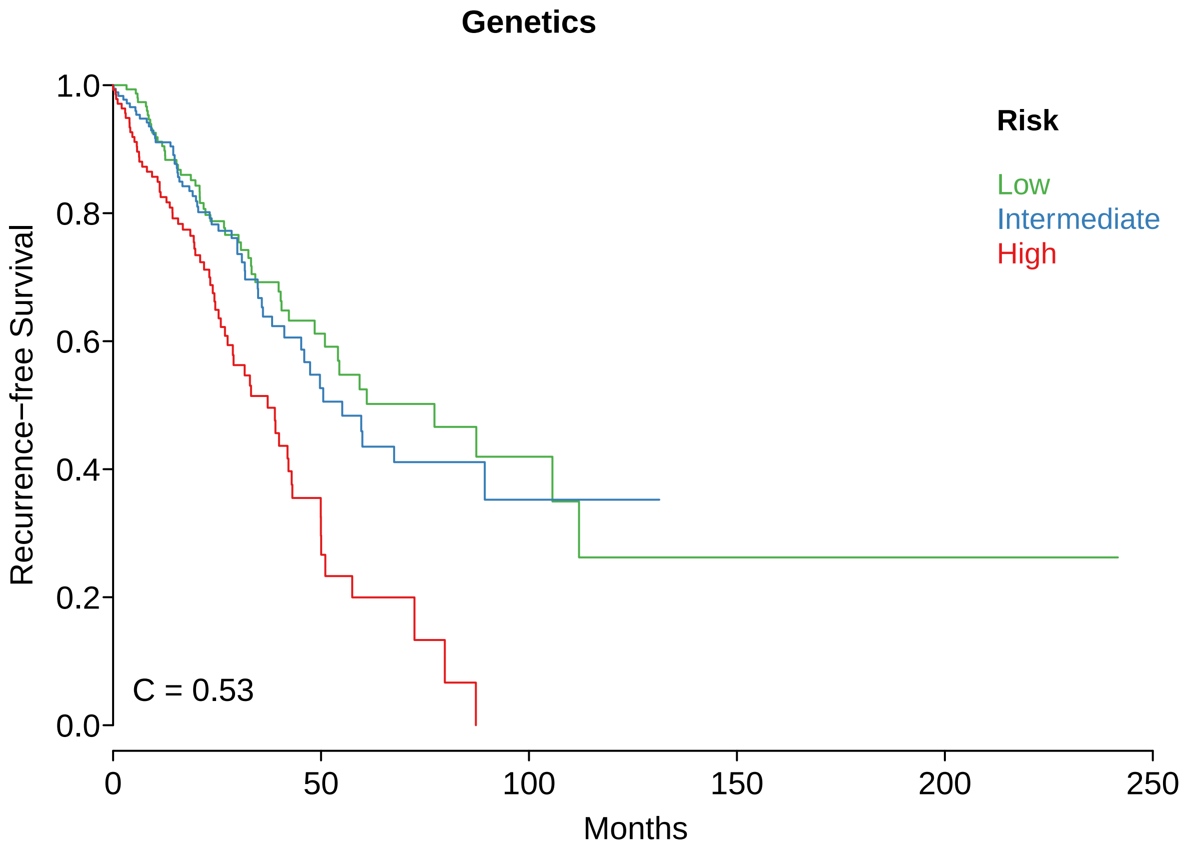

Genetics
Risk
Low
Intermediate
High
Recurrence−free Survival
1.0
0.8
0.6
0.4
0.2
0.0
C = 0.53
0
50
100
150
200
250
Months

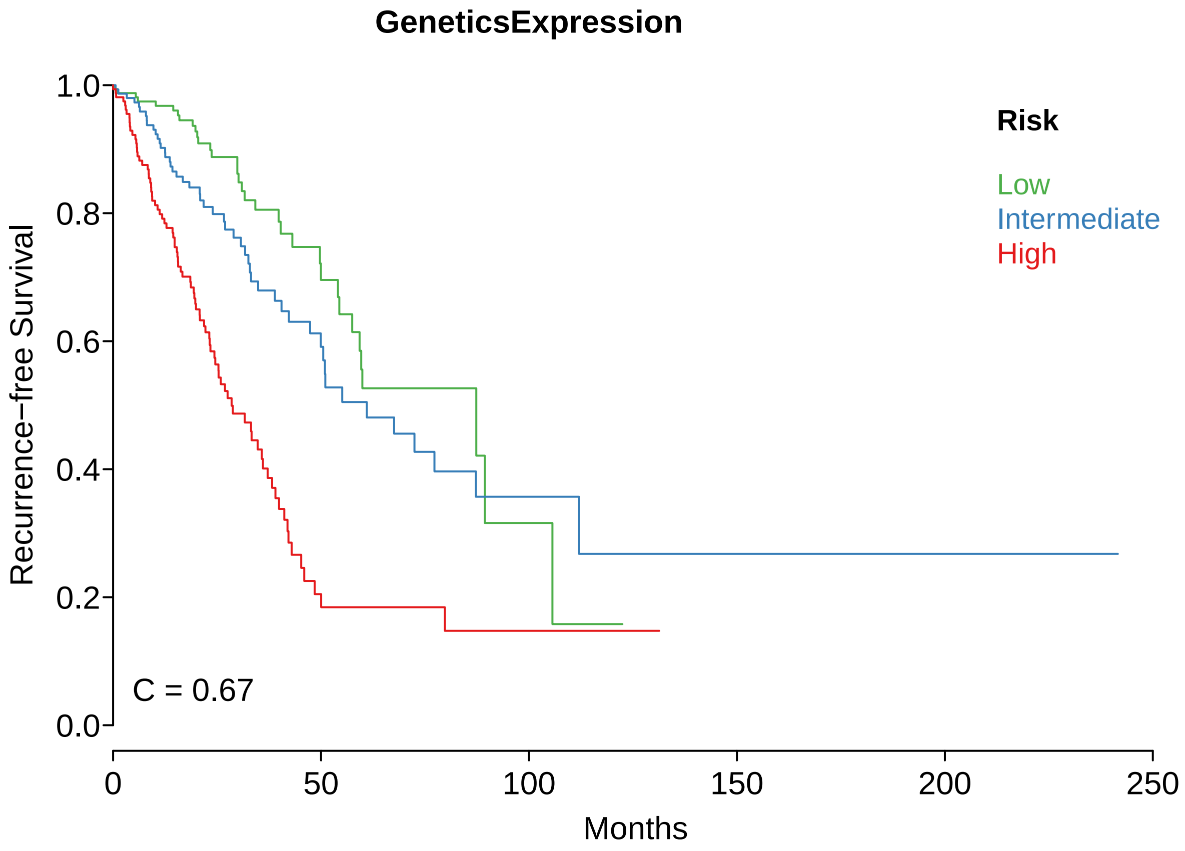
GeneticsExpression
Recurrence−free Survival
1.0
0.8
0.6
0.4
0.2
0.0
C = 0.67
0
50
100
150
200
250
Months
Risk
Low
Intermediate
High

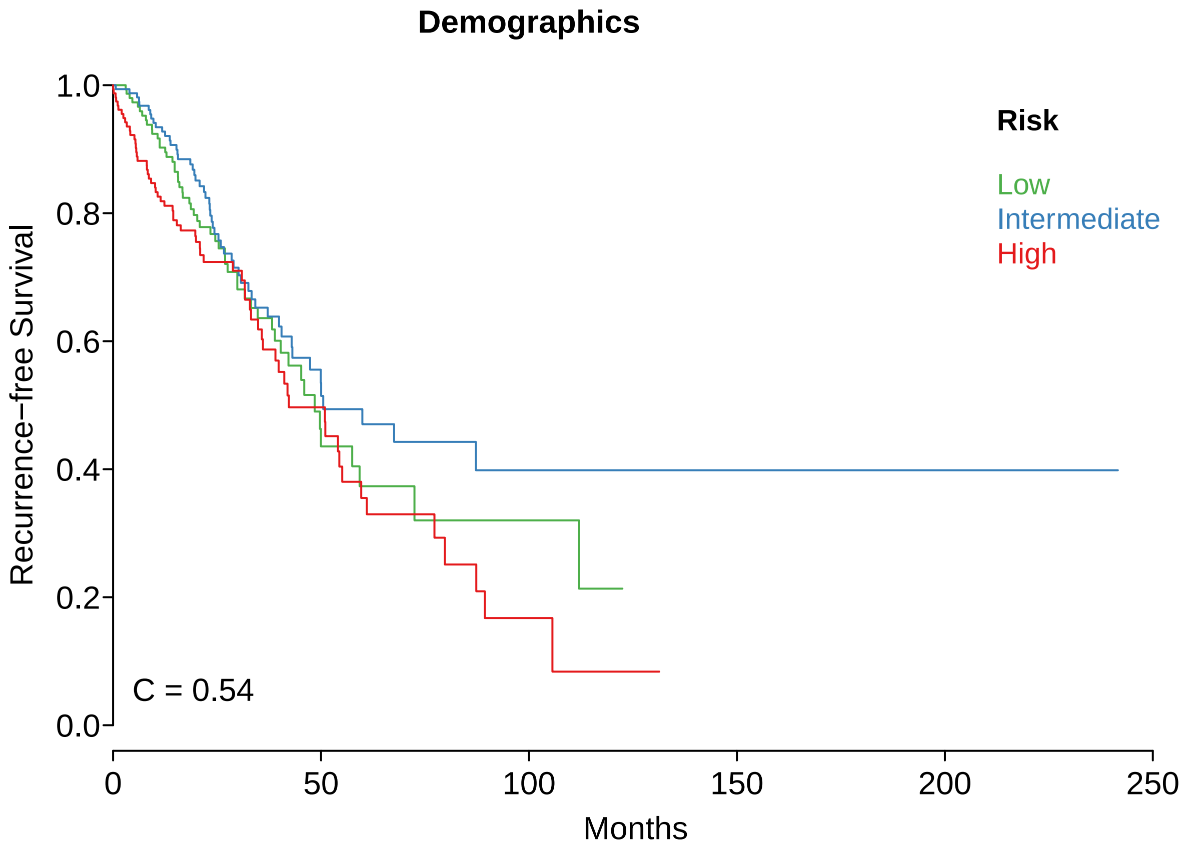
Demographics
Risk
Low
Intermediate
High
1.0
0.8
0.6
0.4
0.2
0.0
Recurrence−free Survival
C = 0.54
0
50
100
150
200
250
Months

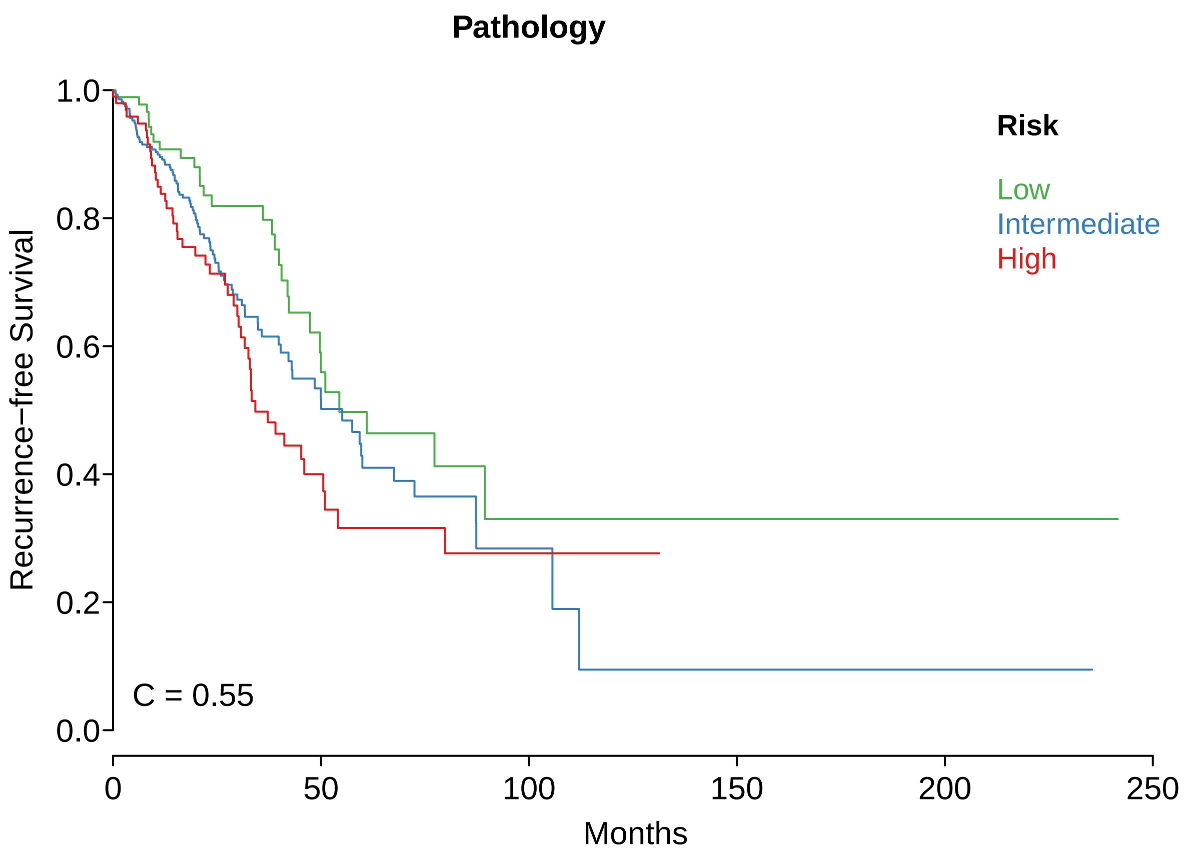
Pathology
Risk
Low
Intermediate
High
Recurrence−free Survival
1.0
0.8
0.6
0.4
0.2
0.0
C = 0.55
0
50
100
150
200
250
Months

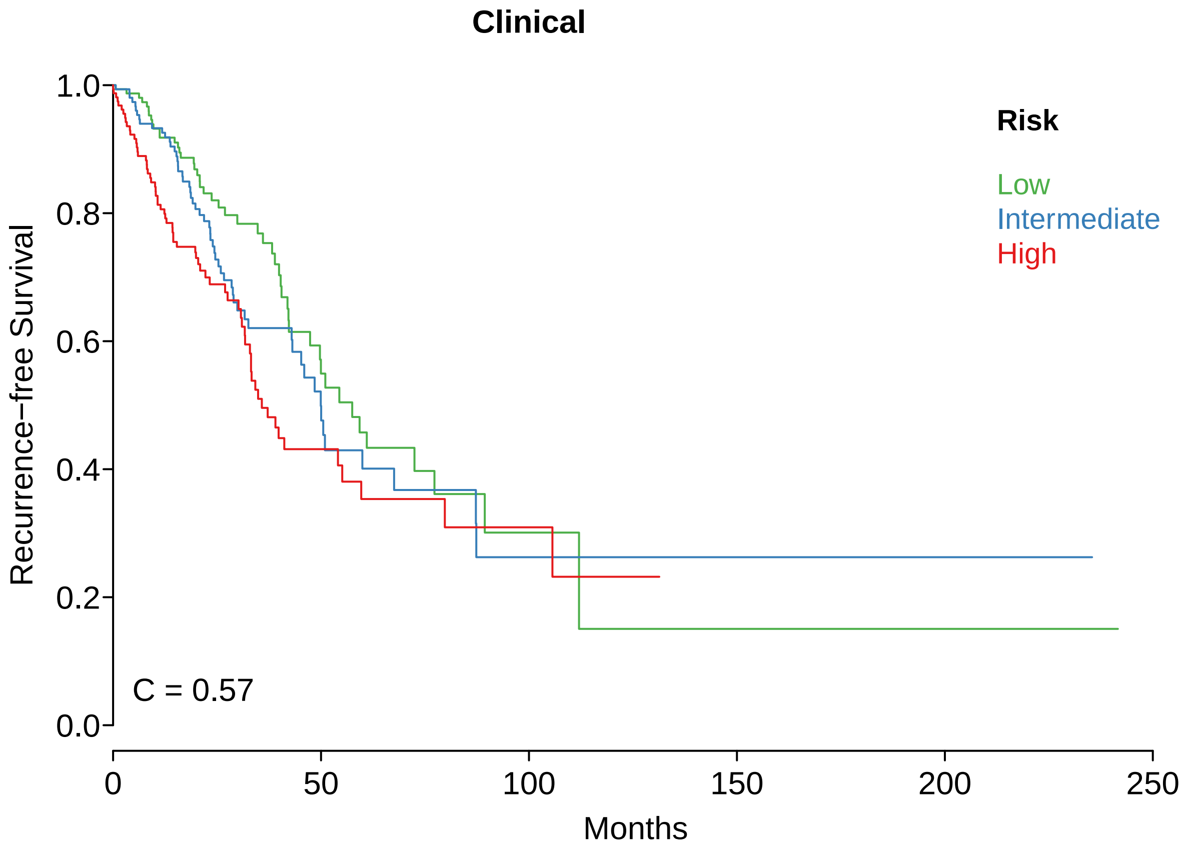
Clinical
Risk
Low
Intermediate
High
Recurrence−free Survival
1.0
0.8
0.6
0.4
0.2
0.0
C = 0.57
0
50
100
150
200
250
Months

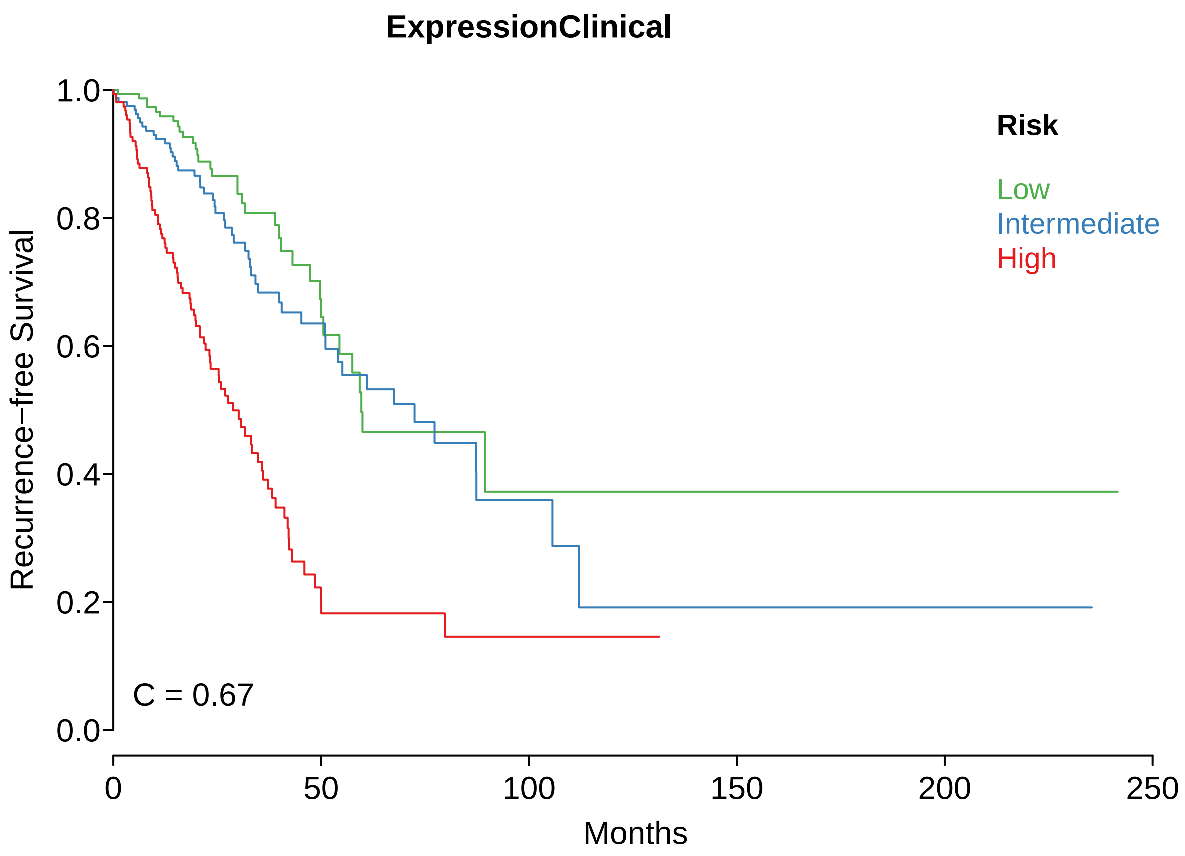
ExpressionClinical
Recurrence−free Survival
1.0
0.8
0.6
0.4
0.2
0.0
C = 0.67
0
50
100
150
200
250
Months
Risk
Low
Intermediate
High

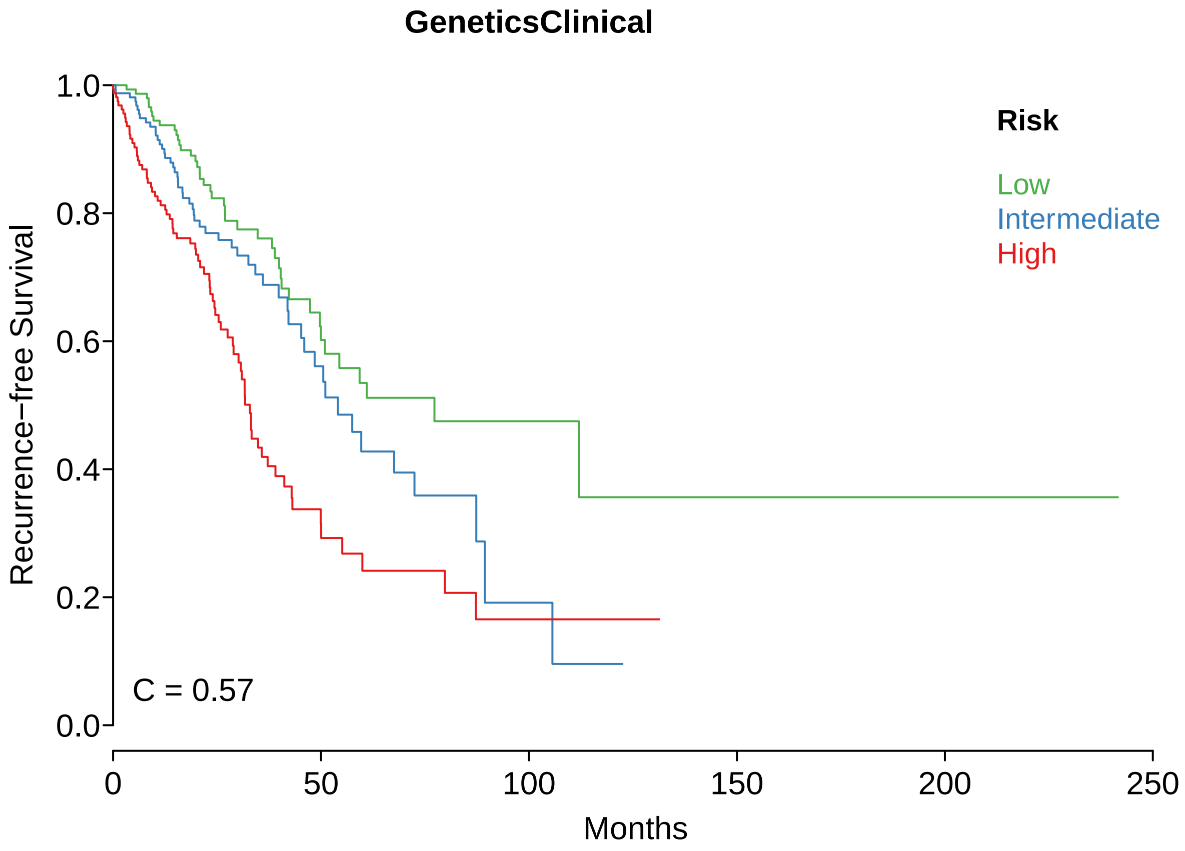

GeneticsClinical
Risk
Low
Intermediate
High
Recurrence−free Survival
1.0
0.8
0.6
0.4
0.2
0.0
C = 0.57
0
50
100
150
200
250
Months

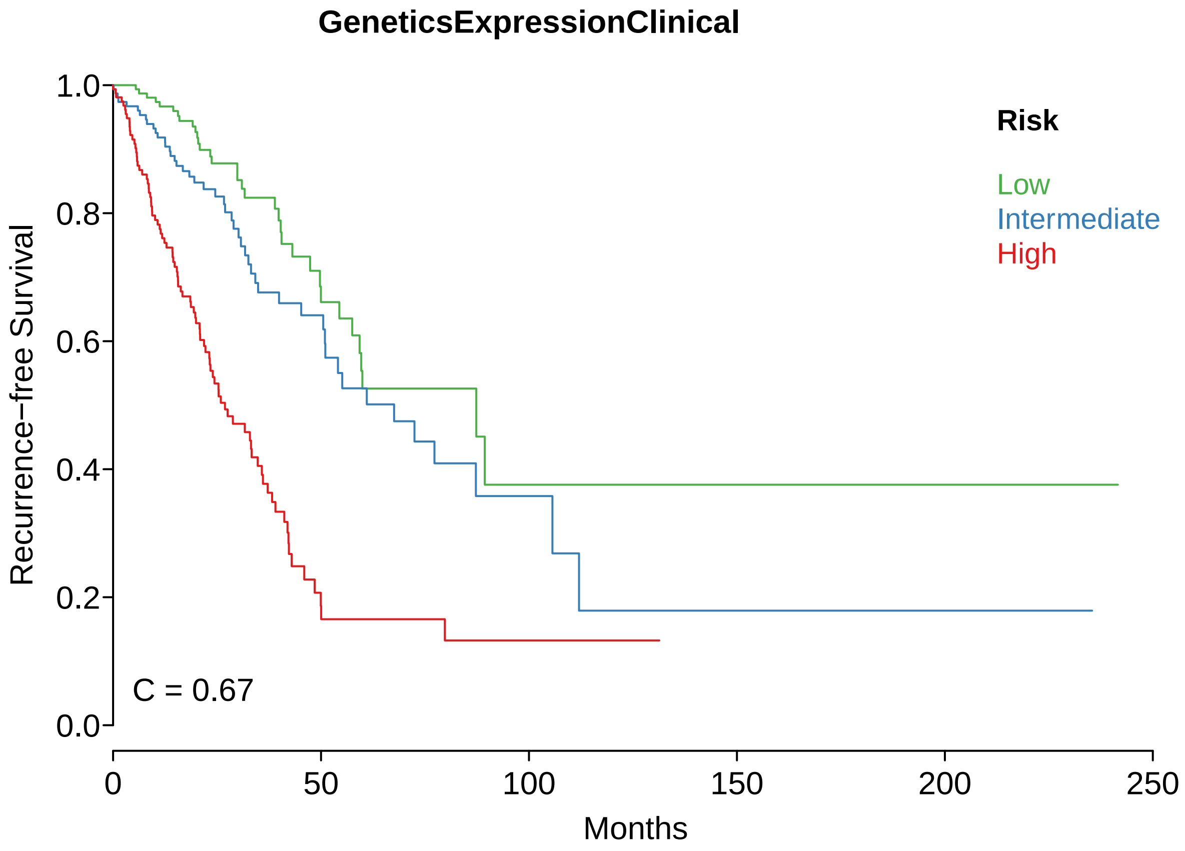
GeneticsExpressionClinical
Recurrence−free Survival
1.0
0.8
0.6
0.4
0.2
0.0
0
50
100
150
200
250
Months
Risk
Low
Intermediate
High
C = 0.67

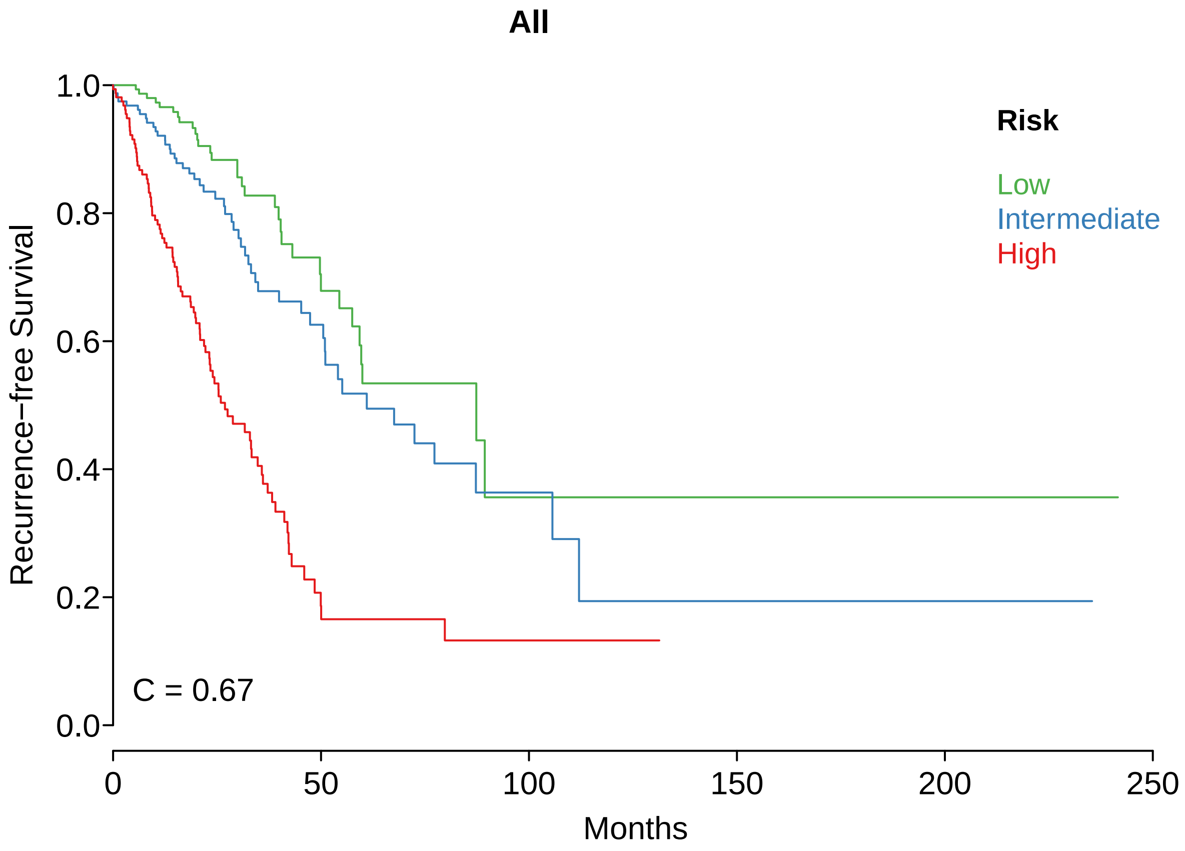

RFS(recurrence-free survival)

```
cli=cli[row.names(Y),]
cli$RFS.time[which(cli$primary_therapy_outcome_success!="Complete Remission/Response")]=NA
cli$RFS[which(cli$primary_therapy_outcome_success!="Complete Remission/Response")]=NA
amlFreeSurvival <- Surv(time=cli$RFS.time, event=cli$RFS)
amlFreeSurvival[,1] <- amlFreeSurvival[,1] /30

set.seed(110)
concordanceCV = data.frame()
cv_ix = sample(1:5, length(rownames(Y)), replace=TRUE)
for(i in unique(cv_ix)){
  c = lapply(Z, function(x) ecoxph(x[cv_ix!=i,], amlFreeSurvival[cv_ix!=i] ))
  p = mapply(function(x,y) as.matrix(x[cv_ix==i,]) %*% coef(y), Z, c)
  concordanceCV = rbind(concordanceCV, apply(-p,2, rcorr.cens, amlFreeSurvival[cv_ix==i])[1,])
}
colnames(concordanceCV) = names(Z)
colMeans(concordanceCV)
```

```
##                expression                  genetics
##                 0.5759332                 0.5474168
##        geneticsExpression              demographics
##                 0.5822082                 0.5137313
##                 Pathology                  clinical
##                 0.4923636                 0.5148987
##        expressionClinical          geneticsClinical
```

```
##                  0.5688040                    0.5426211
## geneticsExpressionClinical                          all
##                  0.5724474                    0.5724474
```

```
hIpss <- rcorr.cens(-stage,amlFreeSurvival)[1]

par(bty="n", mgp = c(2,.33,0), mar=c(5,3,1,1)+3.9, las=1, tcl=-.25, xpd=NA)
h <- concordanceCV[,c("genetics","expression","Pathology","demographics","all")]
v <- sapply(h, sd)/sqrt(5)
colnames(h) <- ""
b <- barplot(c(colMeans(h),hIpss), border=NA,
             col=paste(c(set1[c(2,3,4,1,5,7)],"#BBBBBB"),"88", sep=""),
             las=2, ylim=c(0,0.7), ylab="Harrel's C", names.arg=rep("",6))
points(rep(b[-6], each=5) + seq(-.1,.1,l=5), unlist(h), pch=16, cex=.5, col="darkgrey")
segments(x0=b[-6], y0=colMeans(h)-v, y1=colMeans(h)+v)
rotatedLabel(b, rep(0,6), c("Genetics","Expression","Pathology","Demographics","All","Stage"))
```

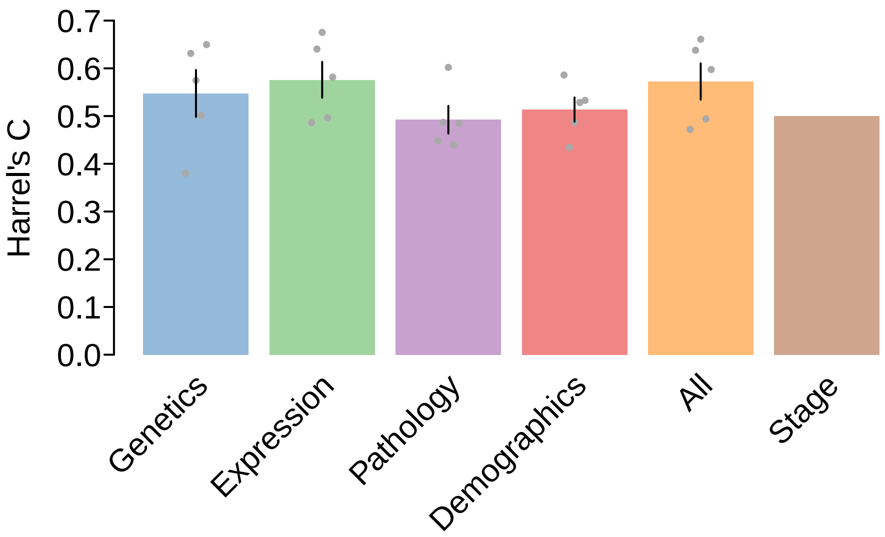

Risk contributions(RFS)

Fit single model

```
groups <- sub("\\..+","",colnames(X))
model <- ecoxph(X, amlFreeSurvival)
index <- TRUE
r <- sapply(unique(groups[index]), function(x) {
  ix <- groups[index] == x
  as.matrix(X[,index][,ix, drop=FALSE]) %*% coef(model)[ix]
```

```
  #+ fullModel$sumX[,x] * fullModel$mu[x]
})

c <- cov(r, use="complete")
x <-  colSums(c)/sum(c)#diag(c / sum(diag(c))) #
x <- x - sum(x[x < 0])
col0 <- c(paste(set1, "88", sep="")[c(3,2,4,1,5)], "grey")
pie(x, col=col0, border=NA, labels = paste(names(x), " (",round(100*x),"%)", sep=""))
polygon(cos(seq(0,2*pi,l=100))*.5, sin(seq(0,2*pi,l=100))*.5, col="white", border=NA)

C <- rcorr.cens(-rowSums(r), amlFreeSurvival)[1]
polygon(c(sin(seq(0,2*pi *C,l=100) +(1-C)*pi)*.4,0),
        c(cos(seq(0,2*pi *C,l=100)+(1-C)*pi)*.4,0),
        col="grey", border=NA)
text(0,-0.1, paste("C=",round(C,2), sep=""), col="white", pos=1)
```

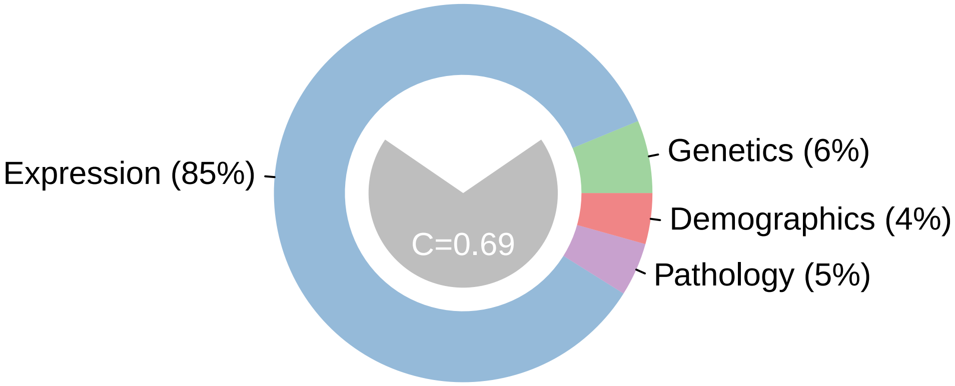

Five-fold cross validation

```
C <- NULL
y <- NULL
set.seed(110)
cv_ix = sample(1:5,length(rownames(Y)), replace=TRUE)
index <- TRUE
for(i in 1:5){
  fit = ecoxph(X[cv_ix!=i,index],amlFreeSurvival[cv_ix!=i], tol=1e-6)
  c = coef(fit)
```

```
  C = c(C,rcorr.cens(-as.matrix(X[cv_ix==i,index][,groups!="Nuisance"]) %*% c[groups!="Nuisance"] ,
                     amlFreeSurvival[cv_ix==i])[1])
  r <- sapply(unique(groups[index]), function(x) {
    ix <- groups[index] == x
    as.matrix(X[cv_ix!=i,index][,ix, drop=FALSE]) %*% coef(fit)[ix]
    #+ fullModel$sumX[,x] * fullModel$mu[x]
  })
  c <- cov(r, use="complete")
  x <-  colSums(c)/sum(c)#diag(c / sum(diag(c))) #
  y <- rbind(y,x)
}
meanC <- mean(C)
x <- colMeans(y)
x <- colSums(c)/sum(c)
x <- x - sum(x[x<0])
pi <- base::pi

par(bty="n", mgp = c(2,.33,0), mar=c(1,3,1,3)+.1, las=2, tcl=-.25)
pie(x, col=col0, border=NA, labels = paste(names(x), " (",round(100*x),"%)", sep=""),
    radius=.8, init.angle=179)
title(main="Survival risk contributions", font.main=1, cex.main=1)
polygon(cos(seq(0,2*pi,l=100))*.5, sin(seq(0,2*pi,l=100))*.5, col="white", border=NA)
polygon(c(sin(seq(0,2*pi *meanC,l=100) +(1-meanC)*pi)*.4,0),
        c(cos(seq(0,2*pi *meanC,l=100)+(1-meanC)*pi)*.4,0),
        col="grey", border=NA)
text(0,0, paste("C=",round(meanC,2), sep=""), col="white", pos=1)
```

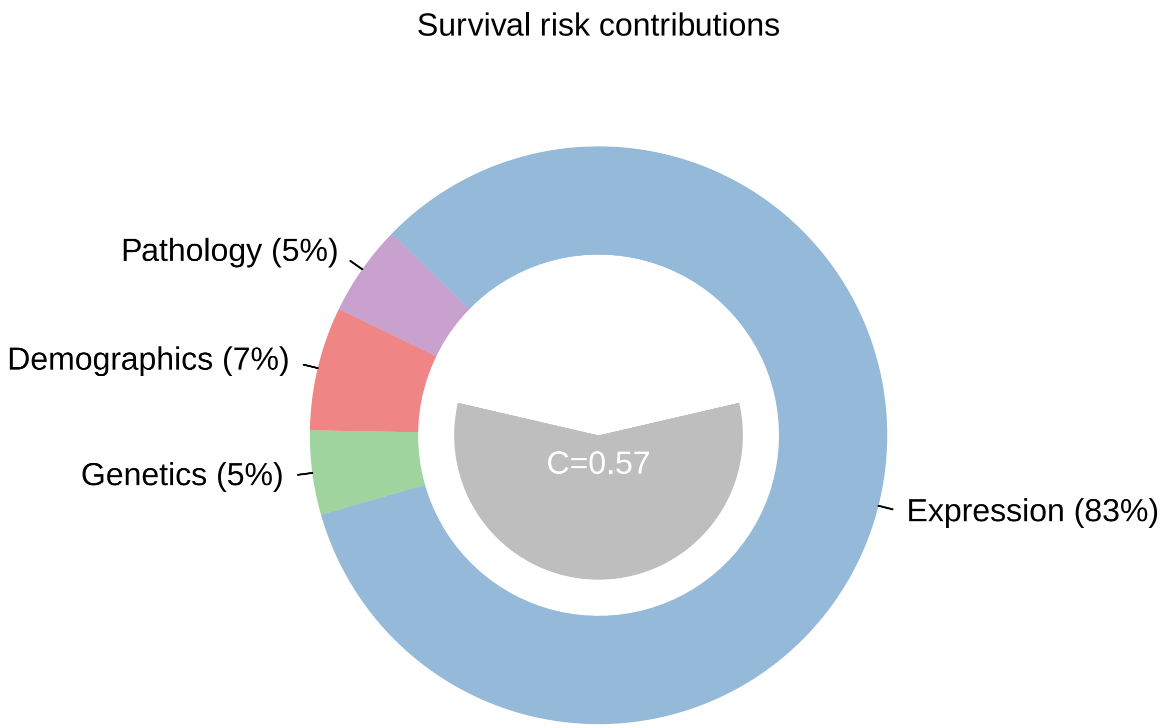

Session

```r
sessionInfo()
```

```
## R version 3.4.4 (2018-03-15)
## Platform: x86_64-w64-mingw32/x64 (64-bit)
## Running under: Windows 10 x64 (build 17134)
##
## Matrix products: default
##
## locale:
## [1] LC_COLLATE=Chinese (Simplified)_China.936
## [2] LC_CTYPE=Chinese (Simplified)_China.936
## [3] LC_MONETARY=Chinese (Simplified)_China.936
## [4] LC_NUMERIC=C
## [5] LC_TIME=Chinese (Simplified)_China.936
##
## attached base packages:
##  [1] grid      parallel  stats4    stats     graphics  grDevices utils
##  [8] datasets  methods   base
##
## other attached packages:
##  [1] GO.db_3.5.0            randomForestSRC_2.6.1  glmnet_2.0-16
##  [4] foreach_1.4.4          Matrix_1.2-14          biomaRt_2.34.2
##  [7] rtracklayer_1.38.3     GenomicFeatures_1.30.3 GenomicRanges_1.30.3
## [10] GenomeInfoDb_1.14.0    VennDiagram_1.6.20     futile.logger_1.4.3
```

```
## [13] gcrma_2.46.0           affy_1.52.0           Hmisc_4.1-1
## [16] ggplot2_3.1.0          Formula_1.2-3         lattice_0.20-35
## [19] xtable_1.8-2           mg14_0.0.5            CoxHD_0.0.61
## [22] survival_2.42-3        cgdsr_1.2.10          RColorBrewer_1.1-2
## [25] hgu133plus2.db_3.2.3   org.Hs.eg.db_3.4.0    AnnotationDbi_1.40.0
## [28] IRanges_2.12.0         S4Vectors_0.16.0      Biobase_2.38.0
## [31] BiocGenerics_0.24.0    limma_3.30.13         reshape2_1.4.3
## [34] knitr_1.22
##
## loaded via a namespace (and not attached):
##  [1] minqa_1.2.4                colorspace_1.3-2
##  [3] rprojroot_1.3-2            htmlTable_1.12
##  [5] XVector_0.18.0             base64enc_0.1-3
##  [7] rstudioapi_0.7             mice_3.1.0
##  [9] affyio_1.44.0              bit64_0.9-7
## [11] mvtnorm_1.0-8              codetools_0.2-15
## [13] splines_3.4.4              R.methodsS3_1.7.1
## [15] mnormt_1.5-5               nloptr_1.0.4
## [17] Rsamtools_1.30.0           broom_0.4.4
## [19] cluster_2.0.7-1            R.oo_1.22.0
## [21] httr_1.3.1                 compiler_3.4.4
## [23] backports_1.1.2            assertthat_0.2.0
## [25] lazyeval_0.2.1             formatR_1.5
## [27] prettyunits_1.0.2          acepack_1.4.1
## [29] htmltools_0.3.6            tools_3.4.4
## [31] bindrcpp_0.2.2             gtable_0.2.0
## [33] glue_1.2.0                 GenomeInfoDbData_1.0.0
## [35] dplyr_0.7.5                Rcpp_0.12.17
## [37] Biostrings_2.46.0          preprocessCore_1.40.0
## [39] nlme_3.1-137               iterators_1.0.9
## [41] psych_1.8.4                xfun_0.5
## [43] stringr_1.4.0              lme4_1.1-17
## [45] XML_3.98-1.11              pan_1.6
## [47] zlibbioc_1.24.0            MASS_7.3-50
## [49] scales_0.5.0               BiocInstaller_1.28.0
## [51] hms_0.4.2                  SummarizedExperiment_1.8.1
## [53] lambda.r_1.2.3             yaml_2.2.0
## [55] memoise_1.1.0              gridExtra_2.3
## [57] rpart_4.1-13               latticeExtra_0.6-28
## [59] stringi_1.1.7              RSQLite_2.1.1
## [61] RMySQL_0.10.15             checkmate_1.8.5
## [63] BiocParallel_1.12.0        matrixStats_0.53.1
## [65] rlang_0.2.1                pkgconfig_2.0.1
## [67] bitops_1.0-6               evaluate_0.13
## [69] purrr_0.2.5                bindr_0.1.1
## [71] GenomicAlignments_1.14.2   htmlwidgets_1.2
## [73] bit_1.1-14                 tidyselect_0.2.4
## [75] plyr_1.8.4                 magrittr_1.5
## [77] R6_2.2.2                   mitml_0.3-6
## [79] DelayedArray_0.4.1         DBI_1.0.0
## [81] pillar_1.2.3               foreign_0.8-70
## [83] withr_2.1.2                RCurl_1.95-4.10
## [85] nnet_7.3-12                tibble_1.4.2
## [87] crayon_1.3.4               futile.options_1.0.1
```

```
## [89] jomo_2.6-2                 rmarkdown_1.8
## [91] progress_1.2.0             data.table_1.11.4
## [93] blob_1.1.1                 digest_0.6.15
## [95] tidyr_0.8.1                munsell_0.5.0
```
